# *ROCS*: a Reproducibility Index and Confidence Score for Interaction Proteomics Studies

**DOI:** 10.1186/1471-2105-13-128

**Published:** 2012-06-08

**Authors:** Jean-Eudes Dazard, Sudipto Saha, Rob M Ewing

**Affiliations:** 1 Division of Bioinformatics, Center for Proteomics and Bioinformatics, Case Western Reserve University, 10900 Euclid Avenue, Cleveland, OH, 44106, USA

**Keywords:** Experimental Reproducibility, Indicator Prey Proteins, Confidence Score, Protein-Protein Interaction, Affinity-Purification Mass-Spectrometry

## Abstract

**Background:**

Affinity-Purification Mass-Spectrometry (AP-MS) provides a powerful means of identifying protein complexes and interactions. Several important challenges exist in interpreting the results of AP-MS experiments. First, the reproducibility of AP-MS experimental replicates can be low, due both to technical variability and the dynamic nature of protein interactions in the cell. Second, the identification of true protein-protein interactions in AP-MS experiments is subject to inaccuracy due to high false negative and false positive rates. Several experimental approaches can be used to mitigate these drawbacks, including the use of replicated and control experiments and relative quantification to sensitively distinguish true interacting proteins from false ones.

**Methods:**

To address the issues of reproducibility and accuracy of protein-protein interactions, we introduce a two-step method, called ROCS, which makes use of *Indicator Prey Proteins* to select reproducible AP-MS experiments, and of *Confidence Scores* to select specific protein-protein interactions*.* The *Indicator Prey Proteins* account for measures of protein identifiability as well as protein reproducibility, effectively allowing removal of outlier experiments that contribute noise and affect downstream inferences. The filtered set of experiments is then used in the Protein-Protein Interaction (PPI) scoring step. Prey protein scoring is done by computing a *Confidence Score*, which accounts for the probability of occurrence of prey proteins in the bait experiments relative to the control experiment, where the significance cutoff parameter is estimated by simultaneously controlling false positives and false negatives against metrics of false discovery rate and biological coherence respectively. In summary, the *ROCS* method relies on automatic objective criterions for parameter estimation and error-controlled procedures.

**Results:**

We illustrate the performance of our method by applying it to five previously published AP-MS experiments, each containing well characterized protein interactions, allowing for systematic benchmarking of *ROCS*. We show that our method may be used on its own to make accurate identification of specific, biologically relevant protein-protein interactions, or in combination with other AP-MS scoring methods to significantly improve inferences.

**Conclusions:**

Our method addresses important issues encountered in AP-MS datasets, making *ROCS* a very promising tool for this purpose, either on its own or in conjunction with other methods. We anticipate that our methodology may be used more generally in proteomics studies and databases, where experimental reproducibility issues arise. The method is implemented in the R language, and is available as an R package called “*ROCS*”, freely available from the CRAN repository http://cran.r-project.org/.

## Background

Affinity-purification mass spectrometry (AP-MS) is a powerful tool for identification of protein complexes and interactions and enables the larger scale analysis of protein networks and cellular processes [1,2]. AP-MS combines the specificity of antibody-based purification of proteins and protein complexes with the sensitivity of mass-spectrometry for identification and quantification, and it has been widely applied to diverse biological systems [2-4]. Although several AP-MS methodology variants have been developed, typical high-throughput work-flows express recombinant epitope-tagged “bait” proteins in cultured cells, recover protein complexes through affinity-purification against the epitope tag, and then identify proteins using LC-MS/MS [5].

Principal challenges in interpreting AP-MS data are the presence of false-positive interacting proteins, misidentification at the MS database search level, and the variability of replicated experiments [6,7]. False-positives in AP-MS data can be classified as external protein contaminants introduced into the samples during sample processing (often common contaminants of mass-spectrometry proteomics experiments such as hair keratins), and contaminants due to the affinity-purification process itself [8]. In the latter case, proteins that are affinity-purified but that are not specific for the bait of interest are hereafter referred to as non-specific prey proteins. These proteins may be purified through their affinity to solid matrices, antibodies or epitope tags employed in the experiment. Carefully designed control experiments may be used to identify non-specific prey proteins, such as cells expressing the epitope tag alone, the parent cells, or cells transfected with the recombinant bait protein but not induced. A second frequent challenge of AP-MS data is interpreting results with low reproducibility. Low reproducibility may result from biological variability of the cells or tissues or technical variability from the affinity-purification or mass-spectrometry. Replicate AP-MS experiments may identify different sets and numbers of proteins. However, selecting only highly reproducible proteins from replicate AP-MS experiments may be too conservative, and novel bait-associated proteins that occur in only a fraction of the replicates may be discarded. The approach described here *carefully* removes outlier experiments in AP-MS data *before* applying final statistical procedures aiming at removing contaminants, i.e. non-specific prey proteins.

Alongside the development of experimental methods to improve AP-MS experiments, several studies have developed computational tools for processing AP-MS data that address the problems of non-specific proteins and providing protein-protein interaction (PPI) confidence scores. Different scoring methods have been developed for prioritizing specific prey proteins in AP-MS data, using quantitative information that typically include a measure of frequency and/or abundance, such as spectral counts [9], and scores from search-engines such as *MASCOT*[10]. Methods such as the Socio-Affinity Index (*SAI*) [11], Normalized Spectral Abundance Factor (*NSAF*) [12], *ComPASS* (*D*-score) [13], *SAINT*[14] and *Decontaminator*[15] have been designed to filter contaminants and assign confidence scores to PPIs. Most of these methods take into consideration the total number of replicated experiments in their scoring systems, although in general without considering the quality control of the replicated experiments. There are statistical methods available to assess reproducibility of large scale LC-MS experiments including the coefficient of variation (CV) and ANOVA [16]. Alternatively, distance measures such as Euclidean distance have been used to measure reproducibility in large scale LC-MS experiments [17]. A recent study from the National Cancer Institute supported Clinical Proteomics Technologies assessment for Cancer (CPTAC) network evaluated reproducibility in inter-laboratory LC-MS/MS proteomics studies [18]. This study concluded that reproducibility is higher for proteins than for peptides, and those factors such as trypsin specificity, peptide ion intensity, and the nature of the corresponding proteins influenced reproducibility in peptide identifications. Although these are useful indicators for all proteomics studies, AP-MS studies, because of their additional sample processing, have additional parameters and complexities such as the presence of non-specific prey proteins that merit special attention, and for which standard statistical measures may not be appropriate.

Current small and medium-scale AP-MS studies typically pair bait experiments with control experiments, in which the bait protein is not expressed. Interacting proteins are then identified as proteins specific to bait experiments or through quantitation, using label-free or isotope-based methods to identify proteins more abundant in bait experiments than control [19]. With larger-scale AP-MS experiments, it becomes possible to define a profile of background, non-specific prey proteins, through for example analysis of a large set of bait AP-MS experiments [13], or a set of control AP-MS experiments [20]. As the capability to perform large-scale AP-MS experiments becomes more widespread, and AP-MS methodologies become more standardized, we anticipate the development of AP-MS dataset repositories, that can be used to define the background profile for specific AP-MS biological systems and AP-MS experiments. The computational problem that we address in this paper is then the problem of distinguishing specific prey proteins in AP-MS data from the background of non-specific preys.

We propose a comprehensive method for selecting reproducible AP-MS replicated experiments and subsequently for identifying bait-specific preys when experimental replicates and control experiments are available. We focus on the analysis of reproducibility in AP-MS experiments, and the improved sensitivity and specificity of prey protein identification that can be achieved by rigorous application of quality control to AP-MS data. We emphasize that an accurate identification of the sought-after bait-specific prey proteins can only be achieved by carefully trading off the sensitivity/specificity through a combined analysis of False Positive and False Negative bait-prey PPIs, and that this is highly dependent on which set of prey proteins is used beforehand to make inferences, i.e. whether one uses the entire prey protein space vs. a selected subspace of prey proteins.

Unlike existing scoring methods, our method *ROCS* first curates the set of experiments used in the scoring, effectively removing replicates that are outliers and therefore eliminating noise that would affect adequate further analysis. First, our method introduces the concept of *Indicator Prey Proteins* that can be used to identify reproducible (or outlier) AP-MS experiments. Second, the method defines the concept of *Confidence Score* to select specific preys by simultaneously controlling the False Discovery Rate (*FDR*) and a measure of biological coherence against biological annotations such as Gene Ontology (*GO*). We show that improved accuracy of predicted interacting proteins can be achieved, where accuracy is to be understood here in the usual classification sense, that is, as the degree to which each new prey protein is correctly classified as a specific or non-specific interacting protein. We use as a test case a subset of systematically generated AP-MS data from a previous large-scale AP-MS study of the human interactome [20] to show that our method may be used on its own or in conjunction with other AP-MS scoring schemes to improve the accuracy of PPI inferences. Our method is also applicable to smaller AP-MS datasets. Indeed, we show that our method is scalable to AP-MS experiments with varying numbers of replicates, and that we are able to determine the minimum number of replicates that an AP-MS study should have in order to make reliable inferences. We illustrate the performance of the method on five separate small-scale AP-MS experiments for which we could successfully identify the most replicated experiments and rank the bait-specific prey proteins. As AP-MS data continues to be acquired and deposited in publicly available repositories (e.g. IntAct molecular interaction database [21]), we anticipate that our method will be applicable to larger sets of AP-MS data as well as other types of proteomics data and databases in general. Finally, the method is available as an R package called “*ROCS*”, freely available from the CRAN repository http://cran.r-project.org/.

## Methods

### Underlying premise

We consider a large scale Affinity Purification-Mass Spectrometry study (bait or control experiments) consisting of a set of *K* uniquely identified *Experimental Replicates*$\left\{E_{1},\ldots ,E_{K}\right\}$. See Additional file 1: Supplemental Methods for more details on the initial input dataset structure [Additional file 1: Supplemental Methods]. Our premise is that prior identification of a subset of most reproducible *Experimental Replicates* greatly improves the differentiation of signal from noise and the ability to identify true specific protein-protein interactions. We show that this is achieved by first finding a set of highly reproducible proteins/peptides.

Probability-based peptide identification statistics such as *MASCOT* peptide identification score [10] is one of the objective measures available for feature (peptide/protein) *identifiability* (i.e. *goodness of identification*). Although high-scoring features are intuitively correlated to higher *experimental reproducibility*, we argue that this is not necessarily the case. In fact, we observed that although measures of feature abundance and feature *reliability*, such as spectral count and search-engine score, are broadly correlated, these measures are not necessarily correlated with feature experimental reproducibility [Additional file 2: Figure S1. This may not come as a surprise since feature *reliability* and experimental feature *reproducibility* are conceptually distinct, and the reason for using *reliability* as a surrogate measure of *reproducibility* has rather been a practical one alone. In addition, how the feature identification score *threshold* (above which features are selected) is chosen in practice, is currently not justified.

We hypothesized that a combined measure of feature *reliability* with reproducibility would not necessarily be *monotonic* with respect to the reliability score threshold used for filtering in the features. By combining the information contained within a measure of feature *reliability*, such as a search-engine feature identification score, with a standardized measure of experimental *reproducibility*, such as the feature frequency of occurrence across experimental replicates, we show that an optimal feature *reliability* score *threshold* can be determined to filter the features in. Moreover, we propose an automatic and objective way of finding this optimal *reliability* score *threshold*, above which one should select the peptides and corresponding proteins. We named these specific proteins/peptides thereby selected as *Indicator Prey Proteins*. They appear in the most replicated AP-MS experiments, thus allowing differentiation of the reproducible experimental replicates from the outliers. The resulting filtered dataset is then used for specificity analysis and removal of non-specific prey proteins.

Also, at least for the application proposed here, probability-based peptide *reliability* statistics such as *MASCOT* peptide identification score [10], and peptide *PROPHET* probability [22] appear somewhat interchangeable, [Additional file 2: Figure S2. In the remainder of the study, we used *MASCOT* peptide identification score as our primary score, although a probability measure is fully compatible with our method and may be used instead.

### Reproducibility index and indicator prey proteins

For a given bait experiment, we consider the initial set of *N Prey Proteins*$\left\{N_{1},\ldots ,N_{N}\right\}$ that can be uniquely identified by their IPI accession number across all their peptides and *Experimental Replicates*$\left\{E_{1},\ldots ,E_{K}\right\}$. We denote this initial procedural stage by “*N*”, standing for “Naïve”. Next, we consider a subset $\left\{P_{1},\ldots ,P_{P}\right\}\subseteq \left\{N_{1},\ldots ,N_{N}\right\}$ of uniquely identified *P Prefiltered Prey Proteins* for which their corresponding *MASCOT* peptide identification scores are greater than the *MASCOT Score Threshold* (*MST*) as described in details in Additional file 1 Supplemental Methods.

To further refine our set of *Prefiltered Prey Proteins* and identify the reproducible experiments, we introduce a *Reproducibility Index* (*RI*), motivated as follows. On the one hand, including lower *MASCOT* scores proteins would include many unreliable proteins and thus degrade reproducibility across *Experimental Replicates*$\left\{E_{1},\ldots ,E_{K}\right\}$. On the other hand, including higher *MASCOT* score-only proteins would include reliable-only proteins across all *Experimental Replicates*$\left\{E_{1},\ldots ,E_{K}\right\}$, that is, fewer proteins in number. The idea is to account for measures of protein *reliability* (from *MASCOT* scores) as well as protein reproducibility (from the frequency of occurrence across experimental replicates). Hence, our *Reproducibility Index*, designed as a normalized (dimensionless) measure of reproducibility, and defined as the average frequency of occurrences of *Prefiltered Prey Proteins* across all *Experimental Replicates* {*E*_*1*_*,…,E*_*K*_} and across their corresponding peptides whose *MASCOT* scores are greater than a given threshold, denoted *s*. It is formally defined for fixed *K* as:

(1) $$RI\left(s\right)=\frac{1}{K\cdot P\left(s\right)}\overset{P\left(s\right)}{\underset{j=1}{\sum }}\overset{K}{\underset{k=1}{\sum }}I\left(P_{j}\in E_{k}\wedge \text{Score}\left(P_{j}\right)\geq s\right)$$

where $P_{j}\in E_{k}$ denotes a unique occurrence of the *Prefiltered Prey Protein P*_*j*_ in *Experimental Replicate E*_*k*_ for *k* ∈{1, … ,*K*}, and where *P*(*s*) denotes the number of *Prefiltered Prey Proteins* for which their corresponding peptide scores are greater than a given peptide score threshold, denoted *s*. I(.) denotes the indicator function throughout the article. Note that *RI* ∈ [0,1] and that higher *RI* represents greater glob reproducibility across *Experimental Replicates*.

It follows from the above that the *Reproducibility Index* (RI) is expected to vary as a function of the *MASCOT* score, when used as a threshold. So, we define the *Reproducibility Index Threshold* (*RIT*) as the peptide *MASCOT* score threshold maximizing the *Reproducibility Index* (*RI*), i.e. formally $RIT=\underset{s\in \left[MST,+\infty \right)}{\arg\max}RI\left(s\right)$. The *Reproducibility Index Threshold* (*RIT*) is used for subseting a set of *Q* uniquely identified proteins from the *Prefiltered Prey Proteins*, which we termed *Indicator Prey Proteins*, denoted by $\left\{Q_{1},\ldots ,Q_{Q}\right\}$, and for which their corresponding peptide *MASCOT* scores are greater than *RIT*:

(2) $$\;\left\{Q_{1},\ldots ,Q_{Q}\right\}=\left\{P_{j},j\in \left\{1,\ldots ,P\right\}:Score(P_{j})\geq RIT\right\}$$

Next, for each *Indicator Prey Protein*, we define its *marginal* inclusion probability, denoted *p*_M_(*j*) for *j* ∈$\left\{1,\ldots ,Q\right\}$, across all *Experimental Replicates*$\left\{E_{1},\ldots ,E_{K}\right\}$. One may now define a subset of *Indicator Prey Proteins* for which their *marginal* inclusion probability is greater than a given marginal inclusion probability threshold ${\tilde{p}}_{\text{min}}$, as well as a corresponding *joint* inclusion probability $p_{\text{J}}({\overset{\sim }{p}}_{\text{min}})$ across all *Experimental Replicates*$\left\{E_{1},\ldots ,E_{K}\right\}.$ Details on definitions and estimates are provided in Additional file 1: Supplemental Methods. Hence, by fixing a marginal inclusion probability threshold ${\tilde{p}}_{\text{min}}$, a subset of highly reproducible *Indicator Prey Proteins* can be identified for which their *marginal* inclusion probability is greater than the ${\tilde{p}}_{\text{min}}$ threshold and their *joint* inclusion probability is relatively high. How this threshold is chosen in any bait or control experiment is described in the subsequent subsection “Setting a Marginal Inclusion Probability Threshold”.

### Identification of reproducible experimental replicates and reproducible prey proteins

One may determine a subset of $\left\{E_{1},\ldots ,E_{K}\right\}$ for which all *Indicator Prey Proteinsjointly* appear in each individual *Experimental Replicate E*_*k*_ for *k* ∈ {1, … , *K*}. We claim that these are the most reproducible experiments, which we term *Reproducible Experimental Replicates*. We denote this subset of *L* experiments by $\left\{F_{1},\ldots ,F_{L}\right\}\subseteq \left\{E_{1},\ldots ,E_{K}\right\}$ where the dependency notation with the marginal inclusion probability threshold ${\tilde{p}}_{\text{min}}$ has been dropped for simplification. For a given ${\tilde{p}}_{\text{min}}$, this cardinal can be estimated as:

(3) $$\hat{L}=\overset{K}{\underset{k=1}{\sum }}I\left(\left\{Q_{1},\ldots ,Q_{Q}\right\},\in ,E_{k}\right)\text{for}{\tilde{p}}_{\text{min}}\in \left[0,1\right]$$

From the reduced sets of *Reproducible Experimental Replicates*$\left\{F_{1},\ldots ,F_{L}\right\}$, one may now select the corresponding subset $\left\{R_{1},\ldots ,R_{R}\right\}\subseteq \left\{P_{1},\ldots ,P_{P}\right\}$ of cardinal *R* of uniquely identified and most reproducible *Prefiltered Prey Proteins*, which appear at least once in *Reproducible Experimental Replicates*$\left\{F_{1},\ldots ,F_{L}\right\}$. In keeping with previous notations and simplifications, we further term this subset by *Reproducible Prey Proteins* and denote it by $\left\{R_{1},\ldots ,R_{R}\right\}$. We denote this procedural stage by “*R*”, standing for “Reproducible”.

### Setting a marginal inclusion probability threshold

The choice of the marginal inclusion probability threshold ${\tilde{p}}_{\text{min}}$ in any bait or control experiment depends on the goal and is guided by some simple considerations. First, one can set this threshold to higher probability levels in order to accommodate larger sets of *Indicator Prey Proteins* as well as *Reproducible Experimental Replicates*. Conversely, this threshold can be set to lower probability levels in order to remove outlier experiments as thoroughly as possible. So, the setting of this threshold controls the level of experimental reproducibility and is a matter of tradeoff between specificity and sensitivity and the goals of the experiment.

Second, in every experiment the marginal inclusion probability threshold ${\tilde{p}}_{\text{min}}$ should be lower bounded so as to get at least a strictly positive number of *Reproducible Experimental Replicates*, that is *L* > 0, where for simplification reasons dependency with respect to ${\tilde{p}}_{\text{min}}$ is dropped, but understood. So, the interval for the marginal inclusion probability threshold ${\tilde{p}}_{\text{min}}$ should always be as follows:

(4) $$\underset{{\tilde{p}}_{\text{min}}}{\arg\text{min}\left\{L>0\right\}}\leq {\tilde{p}}_{\text{min}}\leq 1$$

In practice, we noted that the choice of the marginal inclusion probability threshold has relatively little influence as long as it remains within admissible boundaries (see discussion in Results section and Additional file 2: Figure S6.

### Confidence score and identification of specific prey proteins

The goal of an AP-MS experiment is typically to identify the set of prey proteins known as bait-specific prey proteins. Our approach takes advantage of the previously determined *Reproducible Experimental Replicates* for a fixed marginal inclusion probability threshold ${\tilde{p}}_{\text{min}}$ in both bait and control experiments. One may now derive a new *marginal* inclusion probability for each *Reproducible Prey Protein*, but this time across the reduced set of *Reproducible Experimental Replicates*. Letting superscripts *B* and *C* correspond to the bait and the control experiment respectively and using previous notations, these new marginal inclusion probability thresholds may be defined in both bait and control experiments as ${p^{\prime }}_{\text{M}}^{B}(j)=\mathrm{Pr}\left(R_{j}^{B}\in \left\{F_{1}^{B},\ldots ,F_{L^{B}}^{B}\right\}\right)$ for *j ∈ *{1, …, *R*^*B*^} and ${p^{\prime }}_{\text{M}}^{C}(j)=\mathrm{Pr}\left(R_{j}^{C}\in \left\{F_{1}^{C},\ldots ,F_{L^{C}}^{C}\right\}\right)$ for *j ∈ *{1, …, *R*^*C*^}. Their estimates are given in details in Supplemental Methods [Additional file 1: Supplemental Methods].

Next, we introduce a (non-dimensional) score of individual bait-prey interaction specificity, which we term the *Confidence Score* for the *j*-th prey protein in $\left\{R_{1}^{B},\ldots ,R_{R^{B}}^{B}\right\}$, and for fixed ${\overset{˜}{p}}_{\text{min}}^{B}$ and ${\overset{˜}{p}}_{\text{min}}^{C}$, denoted by $C_{S}(j)$, as follows:

(5) $$C_{s}\left(j\right)=\frac{{{\overset{ˆ}{p}}^{\prime }}_{M}^{B}(j)-{{\overset{ˆ}{p}}^{\prime }}_{M}^{C}(j)}{{{\overset{ˆ}{p}}^{\prime }}_{M}^{B}(j)-{{\overset{ˆ}{p}}^{\prime }}_{M}^{C}(j)}.{{\overset{ˆ}{p}}^{\prime }}_{M}^{B}(j)\text{for}j\in \left\{1,\ldots ,R^{B}\right\}$$

As can be seen, this score is an individual bait-prey interaction specificity measure. It is a standardized ratio accounting for the probability of occurrence of each *Reproducible Prey Protein* relative to the bait and the control experiments, weighted up/down by the *marginal* inclusion probability of each *Reproducible Prey Protein* in the bait experiment alone. In other words, the *Confidence Score* accounts for measures of bait-prey specificity and bait-prey frequency altogether. Note that by definition $C_{S}(j)\in \left[-1,1\right]$, where negative and positive scores correspond to *non-specific* and *specific* interacting prey proteins respectively. In keeping with previous notations, a subset of *Specific Prey Proteins* may be found by taking the *Reproducible Prey Protein* in $\left\{R_{1}^{B},\ldots ,R_{R^{B}}^{B}\right\}$ for which the *Confidence Score* is greater than a specificity cutoff, denoted $C_{S}^{\text{cutoff}}$, which is to be estimated (see next subsection). We denote the subset of *Specific Prey Proteins* by $\left\{S_{1}^{B},\ldots ,S_{S^{B}}^{B}\right\}$ of cardinal set $S^{B}=\left|\left\{S_{1}^{B},\ldots ,S_{S^{B}}^{B}\right\}\right|$, and defined as:

$\left\{S_{1}^{B},\ldots ,S_{S^{B}}^{B}\right\}=\left\{R_{j}^{B},j\in \left\{1,\ldots ,R^{B}\right\}:C_{S}\geq C_{S}^{\text{cutoff}}\right\}$ for

(6) $$C_{S}^{\text{cutoff}}\in \left(0,1\right]$$

We denote this last procedural stage by “*S*”, standing for “Specific”. In practice the *Confidence Score* is computed after the aforementioned identification of unique *Indicator Prey Proteins* and *Reproducible Experimental Replicates*, that is, after pre-specifying marginal inclusion probability thresholds in both control and bait experiments: ${\overset{˜}{p}}_{\text{min}}^{B}$ and ${\overset{˜}{p}}_{\text{min}}^{C}$ as explained in (4). Also, in the search for bait specific Protein-Protein-Interactions (PPIs), the *Confidence Score* cutoff is to be estimated from the data. We show in the following section how to do so automatically by simultaneously controlling the False Discovery Rate (*FDR*) and the Gene Ontology (*GO*) semantic similarity of the candidate preys to the bait.

### Automatic estimation of an optimal confidence score cutoff

To objectively validate any Protein-Protein-Interaction (PPI) identification procedure in AP-MS data analysis, one needs to simultaneously assess the sensitivity/specificity of the final sets of prey proteins identified. In the case of our PPI identification procedure, an optimal *Confidence Score* cutoff $C_{S}^{\text{cutoff}}$ should be objectively estimated in order to yield optimal sensitivity-specificity trade-offs. We observed that higher $C_{S}^{\text{cutoff}}$ values yield better specificity (less False Positive) but lower sensitivity (more False Negative), and vice-versa. The decision is a matter of False Negative - False Positive trade-off.

Although an *FDR* analysis does not control by definition the overall False Positive detections, it can indirectly enable the control of the specificity inherent to such PPI procedure (*Specific Prey Proteins*). By definition, the *FDR* is the expected proportion of the number of erroneous rejected null hypotheses to the total number of rejected null hypotheses in the context of multiple hypotheses testing [23]. Here, the *FDR* corresponds to the expected fraction of falsely identified bait-prey PPIs (given that at least one PPI discovery is made) among all bait-prey PPIs discoveries. Practically, the *FDR* estimate for any given *Confidence Score* cutoff $C_{S}^{\text{cutoff}}$ is computed as: FD^RCScutoff=F^PCScutoff/F^PCScutoff+T^PCScutoff, where F^PCScutoff and T^PCScutoff represent the estimated False Positive and True Positives respectively, and F^PCScutoff+T^PCScutoff represents the estimated Total Positives i.e. all identified bait-prey PPI discoveries. Also, as usual in *FDR* analysis, the theory allows two possible goals [24]: one may fix the *Confidence Score* cutoff $\left(C_{S}^{\text{cutoff}}\right)$ beforehand, then determine the corresponding estimated FD^RCScutoff. Alternatively, one may impose the *FDR* to be bounded to some significance level $\left(F\hat{D}R\left(C_{S}^{\text{cutoff}}\right)\leq \theta \right)$ and then determine which values of the estimated *Confidence Score* cutoff are permissible to keep the *FDR* below that level. In practice, we opted for the first goal of analysis by fixing the *Confidence Score* cutoff to the value achieving the lowest estimated *FDR* (irrespective of any *FDR* bound). In the following, for simplification reasons, dependencies with respect to $C_{S}^{\text{cutoff}}$, ${\overset{˜}{p}}_{\text{min}}^{B}$ and ${\overset{˜}{p}}_{\text{min}}^{C}$, will be dropped, but understood.

To estimate the False Positives PPIs in our *ROCS* procedure and control for specificity, we adopted an approach similar to the “target decoy” approach that is widely used in database searching for estimating false positives and/or false discovery rates [25-27]. The $\hat{F}P$ estimate is calculated by averaging the number of identified bait-prey PPI due to contaminants only from the entire set of control experiments. Technically, the $\hat{F}P$ estimate is computed by applying the entire *ROCS* identification procedure to repeated (without replacement) random samples (of size $N^{B}$) of prey proteins identified from the stage “*N*” of control experiments. The $\hat{F}P$ estimate is then computed as the average number of identified bait-prey PPI above the *Confidence Score* cutoff $\left(C_{S}^{\text{cutoff}}\right)$ expected in the Monte-Carlo replicates. With Monte-Carlo replications from the control experiment, we assume that all *Confidence Score* observed above a given *Confidence Score* cutoff $\left(C_{S}^{\text{cutoff}}\right)$ should be considered as false positives. Details on how FDR estimates are computed are provided in Additional file 1: Supplemental Methods.

To indirectly control for sensitivity in our *ROCS* procedure, we used the most appropriate surrogate measure of biological relevance/coherence that is available in real datasets. Although it cannot gauge the sensitivity for any new datasets where baits and preys or their interactions are functionally uncharacterized, it remains one of the best available ways to benchmark the sensitivity of *ROCS* for all known baits-preys PPIs. This is an estimation technique that is generally missing in analytical methods of AP-MS data. Specifically, we used the Resnik measure of semantic similarity [28], one of the most common semantic similarity measures used with Gene Ontology (*GO*) [29], to assess the biological relevance between a *GO* term from the bait protein and another one for each *Specific Prey Protein*. The pairwise Resnik measure of semantic similarity is a node-based measure relying on a quantitative characterization of information called Information Content (*IC*) [30,31] that is computed between two concepts (denoted *c*_1_ and *c*_2_). The information shared by two concepts is indicated by the Information Content of the concepts that encompass them [28], formally defined as $sim_{\text{Res}}\left(c_{1},c_{2}\right)=\max_{c\in S(c_{1},c_{2})}\left[IC(c)\right]$, where $S(c_{1},c_{2})$ is the set of concepts that encompass both *c*_1_ and *c*_2_. Here, we computed the pairwise Resnik measure of semantic similarity between two Gene Ontology (*GO*) terms, one for the bait protein (denoted *c*_B_) and the other for each *Specific Prey Protein* (denoted *c*_P_), within a given ontology (MF, BP, or CC), and denoted $sim\left(c_{\text{B}},c_{\text{P}}\right)$. The pairwise Resnik measure of semantic similarity between two *GO* terms is simply the Information Content of their most informative common ancestor (MICA) in the ontology [29]. We performed *GO* semantic similarity analyses as a function of the *Confidence Score* cutoff $\left(C_{S}^{\text{cutoff}}\right)$ both for the set of *Specific Prey Proteins* (end stage “*S*”) that was found by considering either the entire set of bait *Experimental Replicates* (naive initial state “*N*”) or the selected set of bait *Reproducible Experimental Replicates* (stage “*R*”).

To assess significance in the difference of *GO* biological relevance between the two groups being compared (“*N*” and “*R*”) for every *Confidence Score* cutoff $C_{S}^{\text{cutoff}}$, we tested the null hypothesis that the median Resnik measures of semantic similarity for the two groups at a given $C_{S}^{\text{cutoff}}$, denoted $sim_{C_{S}^{\text{cutoff}}}\left(c_{\text{B}},c_{\text{P}}\right)$, do not differ statistically. We built 100(1−θ)% Confidence Intervals (CIs) of the median Resnik measure of semantic similarity for the two groups for every *Confidence Score* cutoff $C_{S}^{\text{cutoff}}$, and reject the null hypothesis at the *θ* level if their 100(1−θ)% CIs do not overlap, or if the 100(1−θ)% CI of the difference of their medians does not contain zero. The distance between the CIs of these medians was computed for every *Confidence Score* cutoff $C_{S}^{\text{cutoff}}$ as the difference between the Lower Bound (*LB*) of the 100(1−θ)% CI from the “*R*” stage and the Upper Bound (*UB*) of the 100(1−θ)% CI from the “*N*” stage, formally $d\left(C_{S}^{\text{cutoff}}\right)=LB\left[sim_{C_{S}^{\text{cutoff}}}^{R}\left(c_{\text{B}},c_{\text{P}}\right)\right]-UB\left[sim_{C_{S}^{\text{cutoff}}}^{N}\left(c_{\text{B}},c_{\text{P}}\right)\right]$. So, values of the *Confidence Score* cutoff $C_{S}^{\mathrm{cutoff}}$ for which $d\left(C_{S}^{\mathrm{cutoff}}\right)$ is positive represent significant increase (at the *θ* level) in *GO* biological relevance from stage “*N*” to “*R*”, thereby indicating corresponding choices for the *Confidence Score* cutoff $C_{S}^{\mathrm{cutoff}}$.

To get an *approximate*100(1−θ)% CI for comparing two medians (or their difference), we used McGill et al.'s approximation [32]. The 100(1−θ)% CI of the median can be approximated based on its asymptotic normality, and is said to be rather insensitive to the underlying distribution of the samples. The approximate 100(1−θ)% CI of the median *M* extends to $\hat{M}\pm \left(c/1.08\right)\cdot I\hat{Q}R/\sqrt{n}$, where $\hat{M}$ is the median estimate and $I\hat{Q}R$ is the interquartile range estimate and *n* is the sample size (in a boxplot, this is given by the extent of notches) [32]. To estimate these parameters, *B* Monte-Carlo replicates were performed by repeated random sampling without replacement of a sample of prey proteins from the entire set of bait experiments (“*N*”), of size $S^{B}$, i.e. equal to that of the set of *Specific Prey Proteins* (“*S*”). Finally, *c* is chosen such that$c\in \left[1.386,1.960\right]$ for θ=0.05, depending on how similar group sample sizes and group standard deviations are [32].

Finally, to reach the optimal sensitivity/specificity trade-off for bait-prey PPIs, one finds the optimal estimate $FDR\left({\hat{C}}_{S}^{\mathrm{cutoff}}\right)$ of the *Confidence Score* cutoff achieving simultaneously the lowest estimated $FDR\left({\hat{C}}_{S}^{\mathrm{cutoff}}\right)$, and the largest increase in *GO* semantic similarity distance $d\left({\hat{C}}_{S}^{\mathrm{cutoff}}\right)$, interpreted as a significance measure of increase in Gene Ontology (*GO*) biological relevance. In practice the *Confidence Score* cutoff is to be estimated only within the positive range ${\hat{C}}_{S}^{\mathrm{cutoff}}\in \left(0,1\right)$ since only positive *Confidence Scores* are relevant to find specific PPIs, and in order to avoid the singularity ${\hat{C}}_{S}^{\mathrm{cutoff}}=1$. In addition, a range even shorter than ${\hat{C}}_{S}^{\mathrm{cutoff}}\in \left(0,1\right)$ is often good enough to find the ${\hat{C}}_{S}^{\mathrm{cutoff}}$ estimate.

### Testing stability on multi-scale sets of experimental replicates

To validate our identification procedure, we tested its performance on multiple experimental scales of AP-MS data to see how its output remains “stable” as a function of the number of *Experimental Replicates*. Here, the output was taken as the *joint inclusion probability*$p_{\text{J}}(k,{\tilde{p}}_{\text{min}})$ of *Indicator Prey Proteins* (for which their *marginal* inclusion probability is greater than a given threshold ${\tilde{p}}_{\text{min}}$), computed across all *Experimental Replicates*$\left\{E_{1},\ldots ,E_{k}\right\}$, where $k\in \left[3,K\right]$ is the experimental scale. In our case, maximum experimental scale (*K*) ranged from small (*K* = 3) to large (*K* = 200). In the following, the maximum experimental scale (*K*) and the marginal inclusion probability threshold (${\tilde{p}}_{\text{min}}$) are supposed to be fixed, so we further dropped their dependencies throughout the following formal definitions. Eventually, the test reveals the minimum experimental scale required by an AP-MS experiment in order to reliably assess the *Reproducible Experimental Replicates* (or outliers).

We sought to derive bootstrap estimates of the *joint inclusion probability* of interest by applying the idea of the bootstrap resampling technique [33] to our problem. This technique has been well recognized for instance in cluster analysis in phylogenetic studies [34]. Here, we adapted the idea of bootstrap resampling technique to account for the uncertainty of results caused by sampling error of data. To assess this uncertainty, Efron and Shimodaira recently introduced a correction called the *multiscale* bootstrap resampling method [35-37] to better agree with standard ideas of confidence levels and hypothesis testing and to account for the possible *bias* in the computation of the bootstrap probability value of a cluster. Specifically, we computed a so-called *multiscale unbiased joint inclusion probability* estimate ${ˆp}_{\text{J}}^{*b}(k)$ for each experimental scale $k\in \left[3,K\right]$ by means of $L^{*b}(k)$ bootstrapped *Reproducible Experimental Replicates*, where $b\in \left\{1,\ldots ,B_{1}\right\}$ denotes the bootstrap sample [35-37]. Finally, the entire procedure is repeated *B*_2_ times to get the corresponding mean and standard error estimates ${\overset{Â¯}{p}}_{\text{UJ}}(k)$ and $se({\overset{Â¯}{p}}_{\text{UJ}})(k)$, simply by taking the average over the *B*_2_ replicates. Details on how to compute *multiscale* bootstraps and derive the probability estimates are given in Additional file 1: Supplemental Methods.

The goal was to look at how *multiscale joint inclusion probability* estimates distribute with respect to a range of experimental scales $k\in \left[3,K\right]$, and specifically, whether there was any drop in the *uniformity* of its distribution. A drop in *uniformity* indicates a change-point, denoted ${\hat{K}}_{\text{min}}$, below which the identification procedure is not reliable any more. This corresponds to the minimum scale of *Experimental Replicates* that an AP-MS experiment should have to reliably assess which replicated experiments are reproducible.

### Workflow

**Figure 1:**
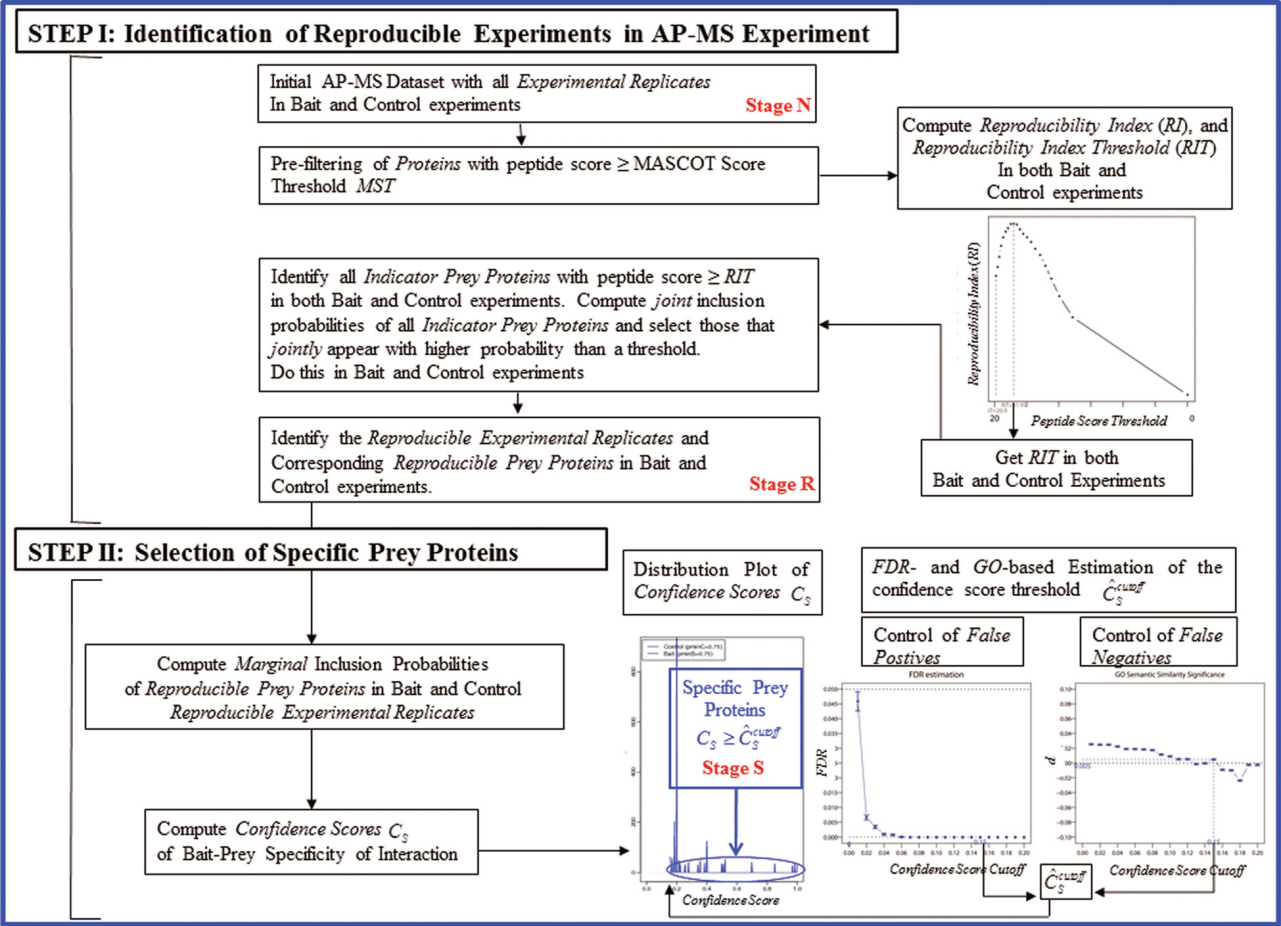
**Overview of the two-step *ROCS *workflow.**

Figure 1 gives the overall integrated workflow of our two-step *ROCS* method for analyzing AP-MS datasets [Figure 1].

## Results and discussion

We use data from a previously published human AP-MS dataset to develop our approach [20]. This dataset corresponds to AP-MS experiments using multiple different bait proteins as well as control AP-MS experiments. Hereafter, superscripts *B* and *C* correspond to the bait and the control experiments respectively. The control dataset consists of $K^{C}=200$*Experimental Replicates* with an initial number of $N^{C}=14429$ control proteins, uniquely identified by their IPI accession numbers, and their corresponding unique control peptide sequences (25114) Additional file 1: Supplemental Methods. Bait AP-MS datasets used here correspond to the following bait genes: VHL, CTNNBIP1, NME2, PPM1B and STK24 with the following initial *Experimental Replicates*: VHL $\left(K^{B}=33\right)$, CTNNBIP1 $\left(K^{B}=9\right)$, NME2 $\left(K^{B}=8\right)$, PPM1B $\left(K^{B}=6\right)$, and STK24 $\left(K^{B}=5\right)$ [Table 1. We illustrate our methodology using the CTNNBIP1 and STK24 dataset since they contain multiple well known interacting prey proteins (such as CTNNB1 and PDCD10 respectively) and *small* numbers of *Experimental Replicates* ($K^{B}=9$ and $K^{B}=5$ respectively) with which we can validate our approach in a realistic way. Complete results of all bait AP-MS datasets are provided in the Supplemental Information. In the CTNNBIP1 and STK24 AP-MS dataset, the initial number of uniquely identified prey proteins was $N^{B}=1229$ and $N^{B}=824$ with corresponding unique prey peptide sequences (1734 and 1369 respectively) [Table 1.

**Table 1:** ***ROCS *results for the number of *Experimental Replicates *and *Prey Proteins *as a function of procedural stages from the initial “Naïve” stage (“*N*”), to the “Reproducible” stage (“*R*”), and to the final “Specific” stage (“*S*”) for all the AP-MS bait experiments: VHL, CTNNBIP1, NME2, PPM1B, and STK24**

**Stages**	**Stage **“***N***”		**Stage **“***R***”		**Stage **“***S***”		
**Bait Experiment**	***Experimental Replicates*** $\left(K^{B}\right)$	***Prey Proteins*** $\left(N^{B}\right)$	***Reproducible Experimental Replicates*** L^B(p˜minB)	***Reproducible Prey Proteins*** $\left(R^{B}({\tilde{p}}_{\min}^{B})\right)$	***Reproducible Experimental Replicates*** $\left({\hat{L}}^{B}({\tilde{p}}_{\min}^{B})\right)$	***Specific Prey Proteins*** $\left(S^{B}({\tilde{p}}_{\min}^{B},{\tilde{p}}_{\min}^{C},C_{S}^{\mathrm{cutoff}})\right)$	$\left({\hat{C}}_{S}^{\mathrm{cutoff}}\right)$
STK24	5	824	3	141	3	112	(0.13)
PPM1B	6	965	3	111	3	76	(0.08)
NME2	8	1349	8	329	8	260	(0.09)
CTNNBIP1	9	1229	5	106	5	69	(0.15)
VHL	33	5398	11	323	11	43	(0.14)

Results are reported for $C_{S}>{\hat{C}}_{S}^{\mathrm{cutoff}}$ and the marginal inclusion probability thresholds ${\tilde{p}}_{\text{min}}^{C}$ and ${\tilde{p}}_{\text{min}}^{B}$ as determined in each AP-MS bait experiment.

### Determination of the reproducibility index and reproducibility index threshold in control and bait experiments

We first plotted the peptide *PROPHET* probabilities versus the peptide *MASCOT* scores in all bait experiments to objectively determine our *MASCOT Score Threshold* (*MST*) and filter out un-reliable peptides (and corresponding proteins) according to the method described in the Methods Section. We have estimated three quartile curves for the B-spline model for visual purposes, but we only used the median regression function for the computation of the *MASCOT Score Threshold*. In the instance of the CTNNBIP1 bait experiment, the median *MASCOT Score Threshold* corresponding to a 50% *Peptide Probability Threshold* was *MST* = 22.34, leaving $P^{B}=180$ uniquely identified proteins [Additional file 2: Figure S2]. Likewise, the median *MASCOT Score Threshold* in the control experiment was *MST* = 20.91, leaving $P^{C}=2400$ uniquely identified proteins. Results for all bait experiments are reported in Additional file 2: Figure S2. We also report the empirical probability density function (PDF) and cumulative density function (CDF) plots of the above peptide scores and peptide probabilities in the AP-MS control and all bait experiments [Additional file 2: Figure S3]. These plots show the locations of the *Peptide Probability Threshold*$\left(Prob^{\left(0.5\right)}=0.5\right)$ and corresponding median *MASCOT Score Thresholds* (*MST*).

**Figure 2:**
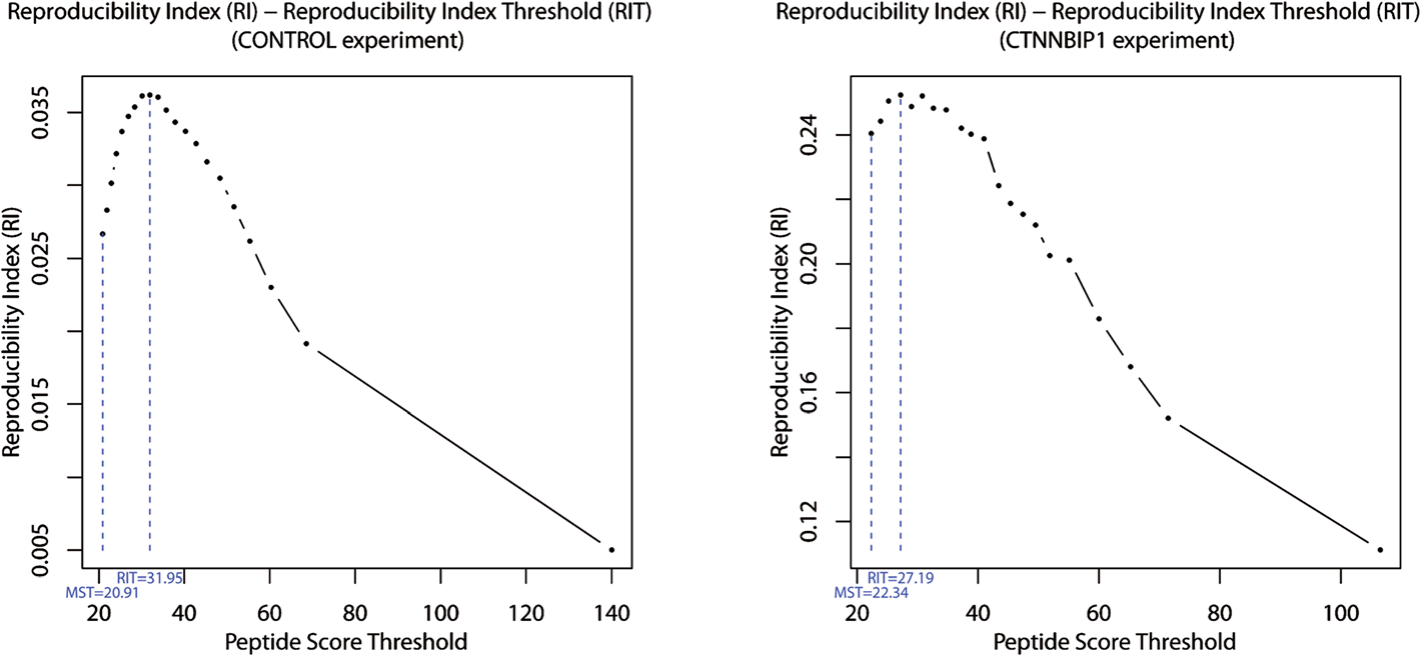
**Optimizing the determination of the peptide *MASCOT *score threshold (*s*** **∈** [***MST***, + **∞)).** The optimal peptide *MASCOT Score Threshold* (*MST*) is shown as well as the *Reproducibility Index Threshold* (*RIT*) with corresponding *Reproducibility Index* (*RI*). Left: CONTROL. Right: CTNNBIP1.

Our initial finding was that the *Reproducibility Index* (*RI*) is not monotonic as a function of the *MASCOT* score threshold. We computed our *Reproducibility Index* (*RI*) in the control and all bait experiments and plotted it against a range of peptide score thresholds $\left(s\in \left[MST,+\infty \right)\right)$. In all experiments tested the quantity of interest (*RI*) always peaks at a certain optimal value of the peptide score threshold, which we have termed *Reproducibility Index Threshold* (*RIT*) [see Methods section, Figure 2 and Additional file 2: Figure S4]. For instance, in the control and in the CTNNBIP1 bait experiments, these *Reproducibility Index Thresholds* were *RIT* = 31.95 and *RIT* = 27.19 respectively [Figure 2].

### Identification of indicator prey proteins, reproducible experimental replicates, and reproducible prey proteins in control and bait experiments

**Figure 3:**
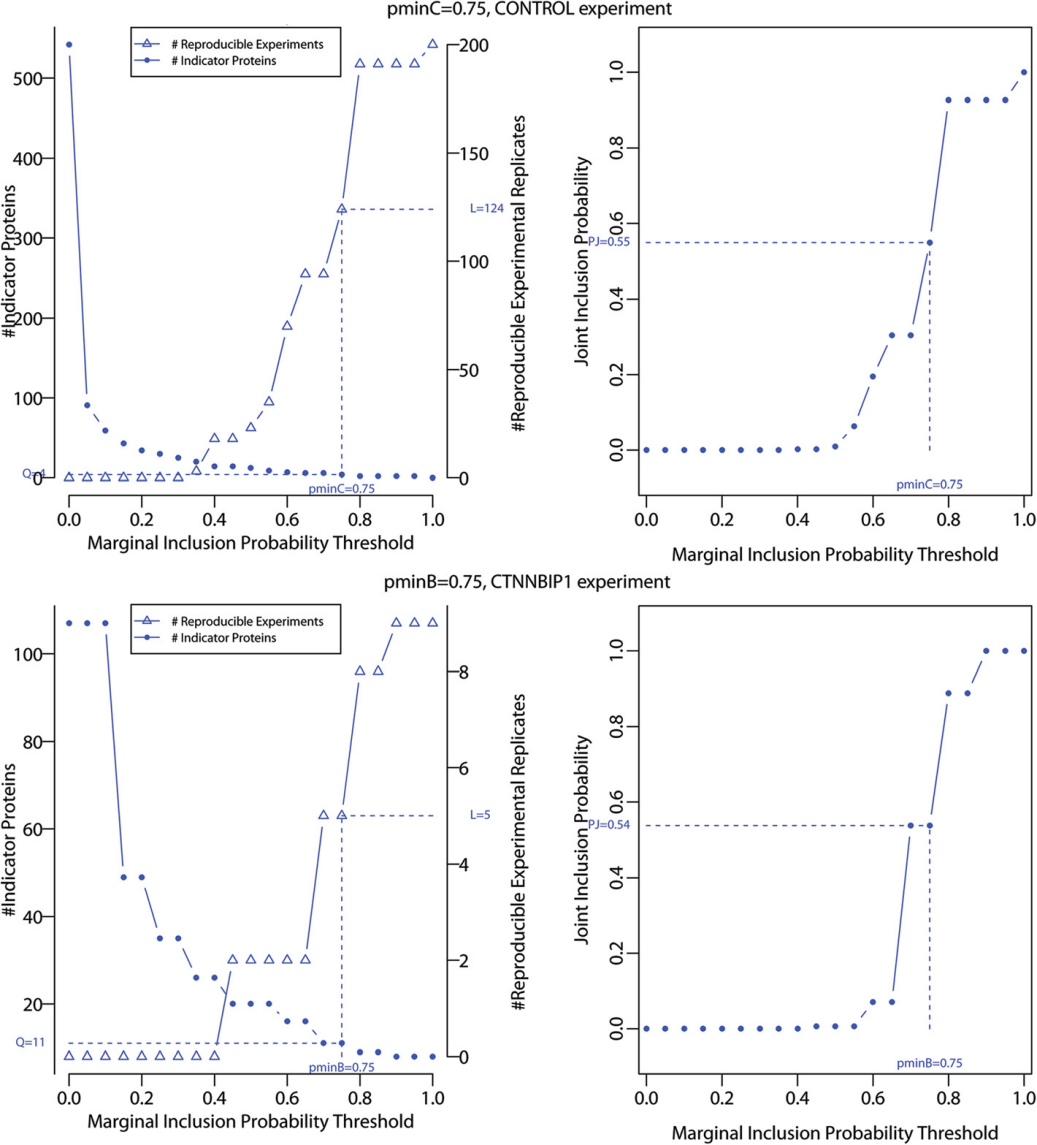
**Number of *Indicator Prey Proteins ***${\hat{Q}}^{C}(0.75)=4$**,**${\hat{Q}}^{B}(0.75)=11$**and *Reproducible Experimental Replicates ***${\hat{L}}^{C}(0.75)=124$**,**${\hat{L}}^{B}(0.75)=5$**(left) and the joint inclusion probability **${\hat{p}}_{\text{J}}^{C}(0.75)=0.55$** and **${\hat{p}}_{\text{J}}^{B}(0.75)=0.54$** (right) in the CONTROL (top) and CTNNBIP1 (bottom) AP-MS bait experiment.** The number of *Indicator Prey Proteins*, *Reproducible Experimental Replicates*, and joint inclusion probabilities are indicated for the marginal inclusion probability thresholds ${\tilde{p}}_{\text{min}}^{C}=0.75$ and ${\tilde{p}}_{\text{min}}^{B}=0.75$.

The *Reproducibility Index Thresholds* (*RIT*) in the control and all bait experiments were used with a range of marginal inclusion probability thresholds ${\tilde{p}}_{\text{min}}^{C}\in \left[0,1\right]$ and ${\tilde{p}}_{\text{min}}^{B}\in \left[0,1\right]$ to further select our so-called set of *Indicator Prey Proteins* in control and bait experiments. We carried out our estimation of the numbers ${\hat{Q}}^{C}({\tilde{p}}_{\text{min}}^{C})$ and ${\hat{Q}}^{B}({\tilde{p}}_{\text{min}}^{B})$ of *Indicator Prey Proteins* with *Reproducible Experimental Replicates*${\hat{L}}^{C}({\tilde{p}}_{\text{min}}^{C})$ and ${\hat{L}}^{B}({\tilde{p}}_{\text{min}}^{B})$ and *joint* inclusion probabilities ${\tilde{P}}_{\text{J}}^{C}({\tilde{p}}_{\text{min}}^{C})$ and ${\tilde{P}}_{\text{J}}^{B}({\tilde{p}}_{\text{min}}^{B})$ respectively in control and bait experiments, each for a given marginal inclusion probability threshold ${\tilde{p}}_{\text{min}}^{C}$ and ${\tilde{p}}_{\text{min}}^{B}$. We report the results for the control and CTNNBIP1 bait experiments in Table 2 and Figure 3 in [Table 2 and Figure 3].

**Table 2:** **Identification of *Indicator Prey Proteins ***${\hat{Q}}^{C}\left({\tilde{p}}_{\min}^{C}\right)$**, **${\hat{Q}}^{B}\left({\tilde{p}}_{\min}^{B}\right)$** and *Reproducible Experimental Replicates ***${\hat{L}}^{C}\left({\tilde{p}}_{\min}^{C}\right)$**, **${\hat{L}}^{B}\left({\tilde{p}}_{\min}^{B}\right)$**, and *joint *inclusion probabilities **${\hat{p}}_{\text{J}}^{C}\left({\tilde{p}}_{\min}^{C}\right)$**, **${\hat{p}}_{\text{J}}^{B}\left({\tilde{p}}_{\text{min}}^{B}\right)$** for a range of marginal inclusion probability thresholds **${\tilde{p}}_{\text{min}}^{C}\in \left[0,1\right]$** and **${\tilde{p}}_{\text{min}}^{B}\in \left[0,1\right]$** in the control and CTNNBIP1 bait experiments respectively**

${\tilde{p}}_{\min}^{C}$**Marginal Inclusion Probability Threshold**	${\hat{Q}}^{C}({\tilde{p}}_{\min}^{C})$ **# Selected Indicator Prey Proteins** **(% of **${\hat{Q}}^{C}$**)**	${\hat{L}}^{C}({\tilde{p}}_{\min}^{C})$ **# Reproducible Experimental Replicates** **(% of **$K^{C}=200$**)**	${\hat{p}}_{\text{J}}^{C}({\tilde{p}}_{\min}^{C})$ **Joint Inclusion Probability**	${\tilde{p}}_{\min}^{B}$ **Marginal Inclusion Probability Threshold**	${\hat{Q}}^{B}({\tilde{p}}_{\min}^{B})$ **# Selected Indicator Prey Proteins** **(% of **${\hat{Q}}^{B}$)	${\hat{L}}^{B}({\tilde{p}}_{\min}^{B})$ **Reproducible Experimental Replicates** **(% of **$K^{B}=9$)	${\hat{p}}_{\text{J}}^{B}({\tilde{p}}_{\min}^{B})$ **Joint Inclusion Probability**
1.00	0 (0.0%)	200 (100.0%)	1.000	1.00	8 (7.5%)	9 (100.0%)	1.000
0.95	2 (0.4%)	191 (95.5%)	0.926	0.95	8 (7.5%)	9 (100.0%)	1.000
0.90	2 (0.4%)	191 (95.5%)	0.926	0.90	8 (7.5%)	9 (100.0%)	1.000
0.85	2 (0.4%)	191 (95.5%)	0.926	0.85	9 (8.4%)	8 (88.9%)	0.889
0.80	2 (0.4%)	191 (95.5%)	0.926	0.80	9 (8.4%)	8 (88.9%)	0.889
*0.75*	**4 (0.7%)**	**124 (62.0%)**	**0.549**	**0.75**	**11 (10.3%)**	**5 (55.6%)**	**0.538**
0.70	6 (1.1%)	94 (47.0%)	0.305	0.70	11 (10.3%)	5 (55.6%)	0.538
0.65	6 (1.1%)	94 (47.0%)	0.305	0.65	16 (15.0%)	2 (22.2%)	0.071
0.60	7 (1.3%)	70 (35.0%)	0.195	0.60	16 (15.0%)	2 (22.2%)	0.071
0.55	9 (1.7%)	35 (17.5%)	0.063	0.55	20 (18.7%)	2 (22.2%)	0.007
0.50	12 (2.2%)	23 (11.5%)	0.009	0.50	20 (18.7%)	2 (22.2%)	0.007
0.45	14 (2.6%)	18 (9.0%)	0.002	0.45	20 (18.7%)	2 (22.2%)	0.007
0.40	14 (2.6%)	18 (9.0%)	0.002	***0.40***	***26 (24.3%)***	***0 (0.0%)***	***0.000***
*0.35*	***20 (3.7%)***	***3 (1.5%)***	***0.000***	***0.35***	***26 (24.3%)***	***0 (0.0%)***	***0.000***
*0.30*	***25 (4.6%)***	***0 (0.0%)***	***0.000***	***0.30***	***35 (32.7%)***	***0 (0.0%)***	***0.000***
*0.25*	***30 (5.5%)***	***0 (0.0%)***	***0.000***	***0.25***	***35 (32.7%)***	***0 (0.0%)***	***0.000***
*0.20*	***34 (6.3%)***	***0 (0.0%)***	***0.000***	***0.20***	***49 (45.8%)***	***0 (0.0%)***	***0.000***
*0.15*	***43 (7.9%)***	***0 (0.0%)***	***0.000***	***0.15***	***49 (45.8%)***	***0 (0.0%)***	***0.000***
*0.10*	***59 (10.9%)***	***0 (0.0%)***	***0.000***	***0.10***	***107 (100.0%)***	***0 (0.0%)***	***0.000***
*0.05*	***91 (16.8%)***	***0 (0.0%)***	***0.000***	***0.05***	***107 (100.0%)***	***0 (0.0%)***	***0.000***
*0.00*	***542 (100.0%)***	***0 (0.0%)***	***0.000***	***0.00***	***107 (100.0%)***	***0 (0.0%)***	***0.000***

Text in italic represents the prohibited choices for the marginal inclusion probability thresholds ${\hat{p}}_{\text{J}}^{C}\left({\tilde{p}}_{\text{min}}^{C}\right)$ and ${\hat{p}}_{\text{J}}^{B}\left({\tilde{p}}_{\text{min}}^{B}\right)$ (see equation (4)), and bold texts represent the choices ${\tilde{p}}_{\text{min}}^{C}=0.75$ and ${\tilde{p}}_{\text{min}}^{B}=0.75$.

In keeping with objective criterion (4), the marginal inclusion probability threshold in the CTNNBIP1 bait experiment was chosen to be ${\tilde{p}}_{\text{min}}^{B}=0.75$, and likewise for the control experiment: ${\tilde{p}}_{\text{min}}^{C}=0.75$ [Table 2 and Figure 3]. For these marginal inclusion probability thresholds, results show that a total of ${\hat{Q}}^{C}(0.75)=3$ and ${\hat{Q}}^{B}(0.75)=8$*Indicator Prey Proteins* could be identified out of a total of ${\hat{Q}}^{C}=542$ and ${\hat{Q}}^{B}=107$ uniquely identified *Indicator Prey Proteins* in the control and CTNNBIP1 bait experiments respectively. Correspondingly, ${\hat{L}}^{C}(0.75)=124$ and ${\hat{L}}^{B}(0.75)=5$*Reproducible Experimental Replicates* were uniquely identified in the control and bait experiment, in which the *Indicator Prey Proteins* jointly appear with a *joint* inclusion probability of ${\hat{p}}_{\text{J}}^{C}(0.75)=0.55$ and ${\hat{p}}_{\text{J}}^{B}(0.75)=0.54$ respectively [Figure 3 and Table 2]. For this combination of thresholds, we determined the subsets $\left\{R_{1}^{B},\ldots ,R_{R^{B}}^{B}\right\}$ and $\left\{R_{1}^{C},\ldots ,R_{R^{C}}^{C}\right\}$ of uniquely identified and most reproducible prey proteins that appear at least once in the sets of *Reproducible Experimental Replicates* in the bait and control experiment respectively. The corresponding cardinal sets were $R^{B}(0.75)=106$ for the CTNNBIP1 bait experiment and $R^{C}(0.75)=1893$ for the control.

We followed a similar identification procedure in all other AP-MS bait experiments and report the results in Tables 1 and 3 and Additional file 2: Figure S5 [Table 1 &3 & Additional file 2: Figure S5]. The lists of *Indicator Prey Proteins* and corresponding *Reproducible Experimental Replicates*, found in all bait experiments, are provided in Additional file 3: Table S1.

**Table 3:** ***ROCS *results for the number of *Indicator Prey Protein*s, *Reproducible Experimental Replicates*, and *joint *inclusion probability **${\hat{p}}_{\text{J}}$** at various experimental scales *K *in each AP-MS control and bait experiments: STK24, PPM1B, NME2, CTNNBIP1, VHL, and CONTROL**

**Bait Experiment with experimental ** **scale *K *(**$K^{C}$**or**$K^{B}$)	${\hat{Q}}^{B}$**or**${\hat{Q}}^{C}$ **# Total Indicator Prey Proteins**	${\tilde{p}}_{\min}^{B}$**or**${\tilde{p}}_{\min}^{C}$ **Bait Marginal Inclusion Probability Threshold**	${\hat{Q}}^{B}({\tilde{p}}_{\min}^{B})$ or ${\hat{Q}}^{C}({\tilde{p}}_{\min}^{C})$ **# Selected Indicator Prey Proteins** **(% of **${\hat{Q}}^{B}$**or**${\hat{Q}}^{C}$**)**	${\hat{L}}^{B}({\tilde{p}}_{\min}^{B})$** or **${\hat{L}}^{C}({\tilde{p}}_{\min}^{C})$ **# Reproducible Experimental Replicates** **(% of **$K^{C}$**or**$K^{B}$)	${\hat{p}}_{\text{J}}^{B}({\tilde{p}}_{\min}^{B})$**or**${\hat{p}}_{\text{J}}^{C}({\tilde{p}}_{\min}^{C})$ **Joint Inclusion Probability**
5 (STK24)	145	0.80	24 (16.6%)	3 ( 60.0%)	0.007
6 (PPM1B)	71	0.80	7 ( 9.9%)	3 ( 50.0%)	0.480
8 (NME2)	145	0.80	4 ( 2.8%)	8 (100.0%)	1.000
9 (CTNNBIP1)	107	0.75	11 (10.3%)	5 ( 55.5%)	0.540
33 (VHL)	597	0.75	15 ( 2.5%)	11 ( 33.3%)	0.070
200 (CONTROL)	542	0.75	4 ( 0.7%)	124 ( 62.0%)	0.550

Results are reported for the marginal inclusion probability thresholds ${\tilde{p}}_{\text{min}}^{C}$ and ${\tilde{p}}_{\text{min}}^{B}$ as determined in each AP-MS bait experiment.

### Confidence score and specific prey proteins in bait experiments

We report here the results for the identification of *Specific Prey Proteins* for instance in the CTNNBIP1 bait experiment. This determination was made at the procedural stage “*S*”, which calls for specifying marginal inclusion probability thresholds ${\tilde{p}}_{\text{min}}^{B}$ and ${\tilde{p}}_{\text{min}}^{C}$ in both control and bait experiments as well as automatic estimation of the *Confidence Score* cutoff.

**Figure 4:**
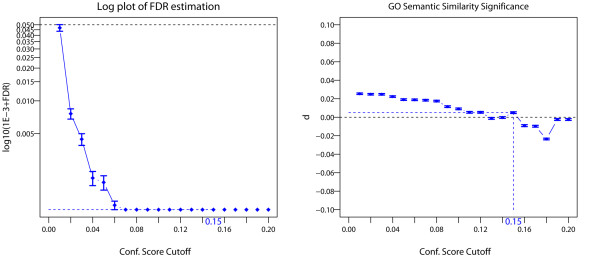
***FDR *and *GO *semantic similarity analyses in the CTNNBIP1 AP-MS bait experiment.** Left: Log-plot of *FDR* estimates for the bait-prey PPIs are plotted against *Confidence Score* cutoffs [see also Additional file 2: Figure S7. Right: plot of distance *d* estimates of bait-prey *GO* semantic similarity measures (see Methods section) are plotted against *Confidence Score* cutoffs [see also Additional file 2: Figure S7. Estimates of log _10_(10^−3^ + *FDR*) and *d* are reported with standard errors in both cases. Horizontal black dotted lines correspond to thresholds of significance levels for *FDR* (*θ* = 0.05) and *GO* (*d* > 0). For the computation of *d*, approximate 95% Confidence Interval of median bait-prey semantic similarities were carried out in the initial set of bait experiments (“*N*” stage) and the set of bait *Reproducible Experimental Replicates* (“*S*” stage) for the Molecular Function (MF) ontology as described in method section. Here *B* = 1024 Monte-Carlo replicates were performed, and a coefficient *c* = 1.386 was chosen for the 95% CI since group sample sizes and group standard deviations were similar [32]. Results are reported for the positive range of *Confidence Score* cutoff *Ĉ*_*S*_^*cutoff*^ ∈ (0, 0.2] and for the marginal inclusion probability threshold ${\tilde{p}}_{\text{min}}^{B}=0.75$.

A first step in the selection of the set of *Specific Prey Proteins* is to automatically estimate the *Confidence Score* cutoff from the data. To look for the *Confidence Score* cutoff achieving simultaneously the lowest estimated *FDR* and the largest *GO* semantic similarity distance $\left(d>0\right)$ (see Methods section), we analyzed the *FDR* and bait-prey *GO* semantic similarity as a function of the *Confidence Score* cutoff over the positive range ${\hat{C}}_{S}^{\mathrm{cutoff}}\in \left(0,0.2\right]$. We noticed that this range was large enough to find the ${\hat{C}}_{S}^{\mathrm{cutoff}}$ estimates in all our bait datasets tested [Figure 4 and Additional file 2: Figure S7]. Also, we computed approximate 95% confidence intervals of semantic similarity for the Molecular Function (MF) ontology and the distance (*d*) of significance between these intervals as described in the Methods section [Figure 4 and Additional file 2: Figure S7]. As can be seen in the CTNNBIP1 bait experiment, an estimated *Confidence Score* cutoff of ${\hat{C}}_{S}^{\mathrm{cutoff}}=0.15$ simultaneously satisfies both objective criteria with a minimal $F\hat{D}R\approx 0\%$ and a positive *GO* semantic similarity distance $\hat{d}=0.005>0$. Higher ${\hat{C}}_{S}^{\mathrm{cutoff}}$ values yield better specificity but lower sensitivity, and vice-versa [Figure 4]. Similar results were obtained for the other four bait experiments [Additional file 2: Figure S7]. Our experience is that the *FDR* and inferences are relatively robust to the choice of the marginal inclusion probability threshold ${\tilde{p}}_{\text{min}}^{B}$ as long as it is within the recommended boundaries (equation (4)) and as long as the *Confidence Score* cutoff (next) is kept strictly positive. This was the case in all our bait experiments - see Tables 1, 2 and 3 and Additional file 2: Figures S6 and S7.

From the previous estimation of GO- and FDR-controlled *Confidence Score* cutoff ${\hat{C}}_{S}^{\mathrm{cutoff}}$ in each bait experiment, we selected the corresponding *Specific Prey Proteins.* In the case of the CTNNBIP1 bait experiment, for the given combination of marginal inclusion probability thresholds (${\tilde{p}}_{\text{min}}^{B}=0.75$, ${\tilde{p}}_{\text{min}}^{C}=0.75$) and *Confidence Score* cutoff (${\hat{C}}_{S}^{\mathrm{cutoff}}=0.15$), the number of *Specific Prey Proteins* was $S^{B}(0.75,0.75,0.15)=69$ (out of a total of $R^{B}(0.75)=106$*Reproducible Prey Proteins*). We followed a similar identification procedure of *Specific Prey Proteins* in all other bait experiments and report the corresponding results in Table 1.

### ROCS performance on prey protein specificity and experimental variability

**Figure 5:**
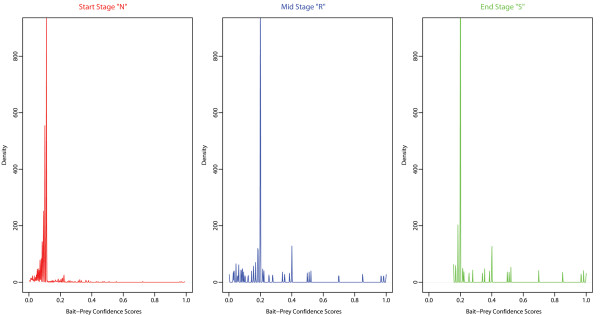
**Density distribution plots of bait-prey *Confidence Scores *at procedural stages “*N*”, “*R*” and “*S*” in the CTNNBIP1 AP-MS bait Experiment.** Note the identical density scales and the re-distribution of *Specific Prey Proteins* (*Confidence Score* → 1) as the method progresses through the procedural stages: from the initial “Naïve” stage (“*N*”), to the “Reproducible” stage (“*R*”), and to the final “Specific” stage (“*S*”). Results are reported for the entire positive range *Ĉ*_*S*_^*cutoff*^ ∈ [0, 1] of *Confidence Score* cutoff and the marginal inclusion probability thresholds ${\tilde{p}}_{\text{min}}^{C}=0.75$ and ${\tilde{p}}_{\text{min}}^{B}=0.75$.

For the given combination of marginal inclusion probability thresholds and *Confidence Score* cutoff, we report the distribution of the bait-prey *Confidence Scores* from the initial “Naïve” stage (“*N*”), the “Reproducible” stage (“*R*”), and the final “Specific” stage (“*S*”) in the bait experiments. Note the accumulation of *Specific Prey Proteins* with higher *Confidence Scores* as the method progresses through the procedural stages: [Figure 5 and Additional file 2: Figure S8].

**Figure 6:**
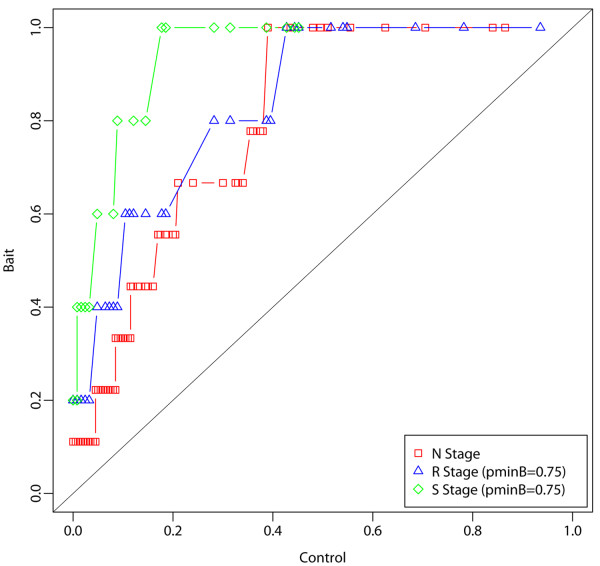
**Quantile-Quantile plot of bait vs. control *marginal *inclusion probabilities in the CTNNBIP1 AP-MS bait Experiment.** Note the increase in quantiles in the bait compared to the control experiment as the method progresses through the procedural stages, denoted by the initial “Naïve” stage (“*N*”), the “Reproducible” stage (“*R*”), and the final “Specific” stage (“*S*”). This corresponds to a separation of bait versus control distributions with an accumulation towards 1 in the bait $\left({{\hat{p}}^{\prime }}_{\text{M}}^{B}(j,0.75)\to 1\right)$ vs. towards 0 in the control $\left({{\hat{p}}^{\prime }}_{\text{M}}^{C}(j,0.75)\to 0\right)$. Results are reported for $C_{S}>{\hat{C}}_{S}^{\mathrm{cutoff}}=0.15$ and the marginal inclusion probability thresholds ${\tilde{p}}_{\text{min}}^{C}=0.75$ and ${\tilde{p}}_{\text{min}}^{B}=0.75$.

We also looked at the distributions of *marginal* inclusion probabilities of all prey proteins in the bait *versus* control experiments through the procedural stages: from the initial “Naïve” stage (“*N*”), to the “Reproducible” stage (“*R*”), and to the final “Specific” stage (“*S*”). Observe the increase in quantiles in the bait compared to the control experiment as the method progresses through the procedural stages in all bait experiments [Figure 6 and Additional file 2: Figure S9]. This corresponds to a clear separation of bait from control distributions and to the accumulations of bait and control *marginal* inclusion probabilities towards 1 and 0 respectively. Overall, these plots pinpoint to an increased segregation of *Specific Prey Proteins* vs. *non-Specific Prey Proteins* by our *ROCS* method as it proceeds through the procedural stages.

Generally, we also observed in all bait experiments that the correlation between protein *MASCOT* identification scores with our measure of experimental reproducibility increases as the method progresses through the procedural stages: from the initial “Naïve” stage (“*N*”), to the “Reproducible” stage (“*R*”), and to the final “Specific” stage (“*S*”) [Additional file 2: Figure S10]. This appears to be due to a reduction in the proportion of protein with the lowest reproducibility (yet highest *MASCOT* identification scores) and indicates a specific reduction of non-reproducible prey proteins as the method progresses through the procedural stages [Additional file 2: Figure S10].

Further, to check the reproducibility in all bait experiments, we compared the overall Coefficient of Variations (CV) of the mean marginal inclusion probabilities of prey proteins across *Experimental Replicates* [Supp. Methods]. This was carried from the initial “Naïve” stage (“*N*”), to the “Reproducible” stage (“*R*”), and to the final “Specific” stage (“*S*”), i.e. with datasets restricted to the set of uniquely identified proteins from either (i) the entire dataset (*N*^*B*^), (ii) the reproducible dataset (*R*^*B*^), (iii) or the specific dataset (*S*^*B*^). Results are reported in Additional file 2: Figure S11 for all AP-MS bait experiments. The coefficient of variation in any bait experiment should not increase significantly (i.e. within the range of sampling variability) with the set of prey proteins (i.e. *metric space*) that is being used: whether one considers the entire *Prey Protein* space (naive initial state “*N*”) vs. a subspace of it, such as the *Reproducible Prey Protein* space (stage “*R*”), or the final *Specific Prey Protein* space (stage “*S*”). Indeed, notice the stability or the decrease in all cases that is achieved from procedural stage “*N*” onward (with the exception of NME2 bait experiment which is discussed below) [Additional file 2: Figure S11].

### Evaluation of ROCS scoring in comparison to the literature

To test the validity of the *ROCS* scoring in all bait experiments, the corresponding lists of *Specific Prey Proteins* were matched against the *BioGRID* references database (version 3.1 - http://thebiogrid.org/) [Additional file 4: Table S2]. The table gives the reciprocal matching of the two lists against each other, i.e. the matching of *BioGRID* database references into the *ROCS* list as well as the reciprocal matching of the *ROCS* list into the *BioGRID* references. Altogether, results for all bait experiments are indicative that when a protein bait interaction has been characterized and published, it is largely confirmed by our *ROCS* scoring [Additional file 4: Table S2].

To quantify the overlap and biological coherence between *ROCS Specific Prey Proteins* and those found in the literature (*BioGRID*), we first looked at the *GO* semantic similarity measure between the two protein lists (denoted *c*_1_ and *c*_2_) of uniquely identified *Specific Prey Proteins* being compared (*ROCS* vs *BioGRID*) for all gene ontologies (Biological Process (BP), Molecular Function (MF), and Cellular Component (CC)), and in all bait experiments. This was done by computing Resnik’s semantic similarity between the two protein lists (denoted *sim*_Re*s*_(*c*_1_, *c*_2_)) according to Wang’s algorithm [38]. Results are reported in Table 4. They show a very strong to maximal (1) semantic similarity measure between the two lists for all bait experiments, indicating a strong biological coherence between *ROCS Specific Prey Proteins* and those found in the literature (*BioGRID*) [Table 4.

**Table 4:** **Overlap and semantic similarity analyses between the *ROCS *and *BioGRID *lists of uniquely identified *Specific Prey Proteins *in all bait experiments**

**Bait Experiment**	***ROCS *Unique *Prey Proteins*** (***N***^***B***^)	***ROCS *Unique *Specific Prey Proteins ***(***S***^***B***^)	***BioGRID *Unique *Specific Prey Proteins ***(***B***)	***BioGRID *Intersection **(***X***)	***BioGRID *Intersection Significance **(***p*-value**)	***BioGRID *Semantic Similarity **(***sim***_**Re***s*_(***c***_**1**_, ***c***_**2**_))		
						**BP**	**MF**	**CC**
STK24	824	112	40	16	1.47041e-05	1.000	0.789	0.853
PPM1B	965	76	20	4	4.92866e-02	0.939	0.730	0.810
NME2	1349	260	26	18	2.58102e-08	0.939	1.000	0.937
CTNNBIP1	1229	69	13	6	3.00192e-05	0.939	1.000	1.000
VHL	5398	43	181	18	3.46865e-16	0.878	0.873	0.874

Hypergeometric test rejection probabilities (*p*-values) are given as well as protein lists semantic similarities for each gene ontology (Biological Process (BP), Molecular Function (MF), and Cellular Component (CC)). *ROCS* lists are reported for *C* − score *C*_*S*_ > *Ĉ*_*S*_^*cutoff*^and the marginal inclusion probability thresholds ${\tilde{p}}_{\text{min}}^{C}$ and ${\tilde{p}}_{\text{min}}^{B}$ as determined in each AP-MS experiment.

Second, we determined the statistical significance of the overlap between the two lists of *Specific Prey Proteins* (*ROCS* vs *BioGRID*). Under the null hypothesis that the two lists are unrelated or that any intersection is due to chance alone (i.e. *ROCS* lists are randomly sampled under the null without replacement), the random variable of the number of common (intersecting) proteins between the two lists, denoted *X*, follows a hypergeometric distribution with parameters *N*^*B*^, *S*^*B*^, and *B*: *X* ∼ *P*(*X* = *x*|*N*^*B*^, *S*^*B*^, *B*), where *P* is given by $P\left(X=x|N^{B},S^{B},B\right)=\left(\begin{array}{l} S^{B}\\ x \end{array}\right)\left(\begin{array}{l} N^{B}-S^{B}\\ B-x \end{array}\right)/\left(\begin{array}{l} N^{B}\\ B \end{array}\right)$, and where the number of unique *ROCS Specific Prey Proteins* and *BioGRID* proteins are denoted by *S*^*B*^ and *B* respectively. The overlap analysis results with corresponding rejection probabilities (*p*-values) are reported in Table 4. They show statistical significance in *all* bait experiments at the *α* = 0.05 significance level, meaning that we can reject the null hypothesis that the intersections found with the literature is due to chance alone, or that the *ROCS* lists are drawn at random [Table 4].

### Comparisons of ROCS scoring to other scoring techniques

To further assess *ROCS*'s effectiveness as a stand-alone method for finding specific protein-protein interaction (PPIs), we also compared its performance to similar methods such as *SAINT*[14] and *ComPASS*[13]. We compared *ROCS’s* lists of *Specific Prey Proteins* in all bait experiments with those obtained by *SAINT* and *ComPASS.* These lists are given in Additional file 5: Table S3 [Additional file 5: Table S3. They are ranked by decreasing significance of protein-protein interactions (PPI) according to each scoring method. Additional file 5: Table S3 also gives the reciprocal matching of *SAINT* and *ComPASS* lists into the *ROCS* list [Additional file 5: Table S3. Using a similar overlap analysis by means of the hypergeometric distribution test and *GO* semantic similarity as above, we determined the significance of the overlap between (i) *ROCS* and *SAINT*, (ii) *ROCS* and *ComPASS* for all gene ontologies (Biological Process (BP), Molecular Function (MF), and Cellular Component (CC)) and in all bait experiments. Overlap analysis and *GO* semantic similarity results in Table 5 [Table 5 show that *ROCS* identified *Specific Prey Proteins* compares very similarly to these methods and that the overlap with *SAINT* and *ComPASS* is statistically significant in all bait experiments tested.

**Table 5:** **Overlap and semantic similarity analyses between the *ROCS’s *lists of *Specific Prey Proteins *with those obtained by *SAINT *and *ComPASS *in all bait experiments** (see also Additional file **5**: **Table S3)**

**Bait Experiment**	***ROCS *Unique *Prey Proteins ***(***N***^***B***^)	***ROCS *Unique *Specific Prey Proteins ***(***S***^***B***^)	***SAINT *Unique *Specific Prey Proteins ***(***B***)	***SAINT *Intersection **(***X***)	***SAINT *Intersection Significance **(***p*-value**)	***SAINT *Semantic Similarity** (***sim***_**Re***s*_(***c***_**1**_, ***c***_**2**_))		
						**BP**	**MF**	**CC**
STK24	824	112	94	26	1.49146e-05	1.000	1.000	0.853
PPM1B	965	76	86	17	4.62432e-05	0.939	0.936	0.823
NME2	1349	260	210	47	1.25292e-2	1.000	1.000	0.937
CTNNBIP1	1229	69	161	8	1.45195e-1	1.000	0.873	0.937
VHL	5398	43	2	2	5.08701e-05	0.661	0.442	0.307
**Bait Experiment**	***ROCS *Unique *Prey Proteins ***(***N***^***B***^)	***ROCS *Unique *Specific Prey Proteins ***(***S***^***B***^)	***ComPASS *Unique *Specific Prey Proteins ***(***B***)	***ComPASS *Intersection **(***X***)	***ComPASS *Intersection Significance **(***p*-value**)	***ComPASS *Semantic Similarity ** (***sim***_**Re***s*_(***c***_**1**_, ***c***_**2**_))		
						**BP**	**MF**	**CC**
STK24	824	112	169	90	4.34990e-57	1.000	1.000	1.000
PPM1B	965	76	159	54	1.07087e-31	1.000	1.000	1.000
NME2	1349	260	242	162	2.50264e-87	1.000	1.000	0.937
CTNNBIP1	1229	69	146	44	2.00480e-27	1.000	1.000	1.000
VHL	5398	43	740	31	2.01445e-20	0.878	0.873	0.823

Using a similar overlap analysis by means of the hypergeometric distribution test and *GO* semantic similarity as in Table 4, we determined the statistical significance of the overlap between: (Top ) *ROCS* vs. *SAINT*, and (Bottom) *ROCS* vs. *ComPASS* for all gene ontologies (Biological Process (BP), Molecular Function (MF), and Cellular Component (CC)) and in all bait experiments. *SAINT* and *ComPASS* lists are reported for *P*_*SAINT*_ > 0 and *D* − score > 0 respectively. *ROCS* lists are reported for *C* − score *C*_*S*_ > *Ĉ*_*S*_^*cutoff*^and the marginal inclusion probability thresholds ${\tilde{p}}_{\text{min}}^{C}$ and ${\tilde{p}}_{\text{min}}^{B}$ as determined in each AP-MS experiment.

### Effect of ROCS improvement in combination with another scoring technique

Next, we combined our method with an existing procedure for analysis of AP-MS data in the following way. Although there are multiple methods for evaluation of AP-MS data (see Introduction), we use the probabilistic scoring approach *SAINT* developed by Choi *et al*. [14] since it allows for multiple replicated experiments and is scalable to AP-MS datasets of different size. In *SAINT*, we tested the datasets of all bait experiments at every procedural stage of our method, while keeping the same set of control experiments. *SAINT* works indeed with a *small* set of control experiments, which were chosen as the five most replicated ones from the initially pool of *K*^*C*^ = 200 control *Experimental Replicates* (unfiltered dataset). Also, in the latest implementation of *SAINT* (version 2.2.3, http://saint-apms.sourceforge.net/Main.html; Dr. Hyungwon Choi, *pers. comm*.) we were able to use the *MASCOT* scores directly as quantitative input rather than spectral counts as used in earlier versions.

An objective way to assess the performance of our *ROCS* method is to do a side-by-side comparison of *SAINT* protein-protein interaction scoring (ranked by *SAINT* posterior probability *P*_*SAINT*_) between *SAINT*-only and *SAINT* applied in conjunction with *ROCS* at different procedural stages, namely, the initial “Naïve” stage (“*N*”), the “Reproducible” stage (“*R*”), and especially the final “Specific” stage (“*S*”). The comparison is given in Additional file 6: Table S4, where we show the results of the pairwise Resnik semantic similarity measure *sim*(*c*_B_, *c*_P_) between a Gene Ontology (*GO*) term from the bait protein (denoted *c*_B_) and for each *Specific Prey Protein* (denoted *c*_P_) as described in the methods section. This was computed for all Gene Ontologies (Biological Process (BP), Molecular Function (MF), and Cellular Component (CC)) and all bait experiments [Additional file 6: Table S4].

**Figure 7:**
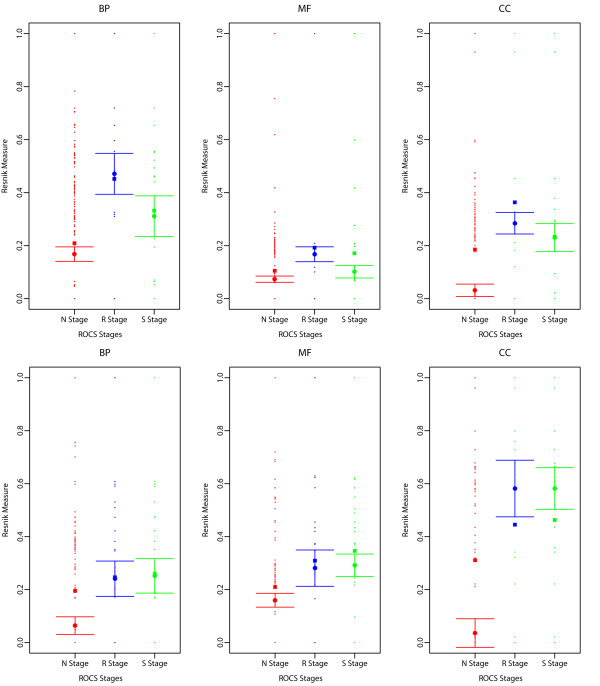
**Confidence Intervals (95% CIs) of the median bait-prey semantic similarity for *SAINT*-only and *SAINT *in conjunction with *ROCS *at the different *ROCS *procedural stages “*N*” (*SAINT*-only), “*R*”, and “*S*” (*ROCS*-*SAINT*) in the CTNNBIP1 (top) and STK24 (bottom) AP-MS bait experiments.** Note especially the increase in median bait-prey semantic similarity between the naïve stage “*N*” (*SAINT*-only) and final stage “*S*” (*ROCS*-*SAINT*). The pairwise Resnik semantic similarity measure $sim\left(c_{\text{B}},c_{\text{P}}\right)$ is given between a Gene Ontology (*GO*) term from the bait protein (denoted *c*_B_) and for each *Specific Prey Protein* (denoted *c*_P_) as described in the methods section for all Gene Ontologies (Biological Process (BP), Molecular Function (MF), and Cellular Component (CC)) - see also Additional file 6: Table S4. Results are reported here for a conservative coefficient of c=1.960 for the 95% CI of the median since group sample sizes and group standard deviations were *not* similar (see Methods section), and for $C_{S}>{\hat{C}}_{S}^{\mathrm{cutoff}}$ and the marginal inclusion probability thresholds ${\tilde{p}}_{\text{min}}^{C}$ and ${\tilde{p}}_{\text{min}}^{B}$ as determined in each AP-MS experiment (see Tables 2 and 3).

We next plotted the 95% confidence interval (CI) of the median bait-prey semantic similarity as described in the methods section. The pairwise Resnik semantic similarity measure *sim*(*c*_B_, *c*_P_) is given between a Gene Ontology (*GO*) term from the bait protein (denoted *c*_B_) and for each *Specific Prey Protein* (denoted *c*_P_) as described in the methods section. This was computed for all Gene Ontologies (Biological Process (BP), Molecular Function (MF), and Cellular Component (CC)). Results show a statistically significant increase in the *GO* bait-prey semantic similarity as *ROCS* is applied in conjunction to *SAINT*, i.e. between *SAINT*-only *(ROCS* procedural stage “*N*”), and *ROCS*-*SAINT* (*ROCS* procedural stage “*S*”) [Figure 7 & Additional file 2: Figure S12].

**Figure 8:**
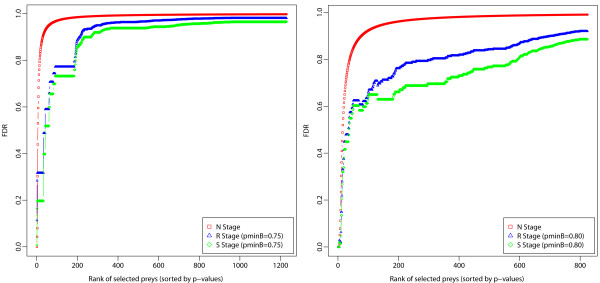
***FDR *computed from *SAINT *posterior probability output (**$P_{\mathrm{SAINT}}$**) as a function of procedural stages: from the initial “Naïve” stage (“*N*” – *SAINT*-only), to the “Reproducible” stage (“*R*”), and to the final “Specific” stage (“*S*” – *ROCS-SAINT*) in the CTNNBIP1 (left) and STK24 (right) AP-MS bait experiment.** Results are reported for $C_{S}>{\hat{C}}_{S}^{\mathrm{cutoff}}$ and the marginal inclusion probability thresholds ${\tilde{p}}_{\text{min}}^{C}$ and ${\tilde{p}}_{\text{min}}^{B}$ as determined in each AP-MS experiment (see Tables 1 and 3).

An alternative way to empirically assess the performance of our *ROCS* method in any bait experiment is to compare the behavior of the False Discovery Rates (*FDR*) between *ROCS* procedural stages, independently or in combination with another scoring method. Here, we used the posterior probability *P*_*SAINT*_ of true protein-protein interaction from the *SAINT* output to get 1 − *P*_*SAINT,*_ which serves as the *local* False Discovery Rate [39] (denoted *lFDR*). Aggregate *FDR* up to the chosen rank is the integral of local *FDR* across the selection, or, in practice, the average of 1 − *P*_*SAINT*_ across the selection. The latter is closer to what's called Bayesian *FDR*, and conceptually to *lFDR*/*FDR*. Results show that when *ROCS* is used in conjunction with *SAINT*, one substantially reduces the *FDR* [Figure 8 and Additional file 22: Figure S13.

### Testing stability on multiscale sets of experimental replicates

**Figure 9:**
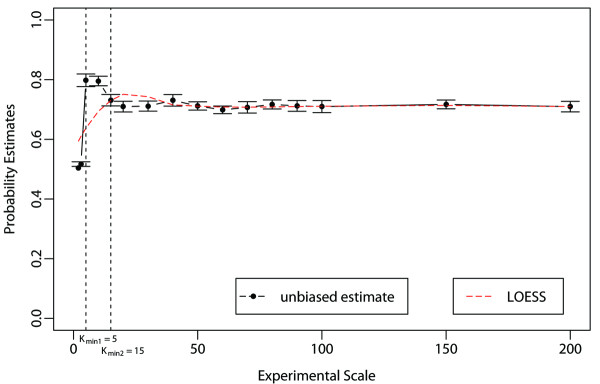
**Stability diagnostic plot of the identification procedure on multiscale sets of experiments. ***Multiscale unbiased joint inclusion probability* estimates is plotted against a range of experimental scale values $K\in \left[2,200\right]$ for an arbitrary marginal inclusion probability threshold of ${\tilde{p}}_{\text{min}}=0.75$. The plot also displays probability estimate standard errors, the change point ${\hat{K}}_{\text{min}}$ (${\hat{K}}_{\text{min}}\in \left[5,15\right]$) and the scatterplot smoothing curves fitted to the data (by LOESS local polynomial regression procedure). These curves are approximately horizontal-flat (i.e. show no trend) for K > Kmin.

To demonstrate the usability of our identification procedure, we tested its performance on several AP-MS bait datasets with varying numbers of *Experimental Replicates*: VHL (*K*^*B*^ = 33), CTNNBIP1 (*K*^*B*^ = 9), NME2 (*K*^*B*^ = 8), PPM1B (*K*^*B*^ = 6), STK24 (*K*^*B*^ = 5). Table 3 and Figure 9 report the results [Table 3, Figure 9].

To test the stability of our method on a larger set of replicated AP-MS experiments, we make use of a set of 200 replicated control AP-MS experiments. Figure 9 shows a stability plot for AP-MS datasets with different numbers of *Experimental Replicates k* ∈ [2, *K*] for fixed *B*_1_ = 10, *B*_2_ = 128, *K* = 200 (see Methods section), an arbitrary *α* = _0.5_*th*-quantile of peptide probabilities, and an arbitrary marginal inclusion probability threshold of e.g. ${\tilde{p}}_{\text{min}}=0.75$ [Figure 9]. As demonstrated by the stability diagnostic plot, *unbiased* “multiscale” joint inclusion probability estimates are very stable across a wide range of experimental scale values. Only the unbiased *multiscale unbiased joint inclusion probability* estimate should be trusted. In fact, although regular (biased) bootstrap estimates show a similar behavior over the range ${\hat{K}}_{\text{min}}\in \left[5,15\right]$, there is a clear inflation of the *joint inclusion probability* estimate for lower experimental scale values (data not shown). As expected, the stability of the unbiased estimate is lost for the smallest *k* values [Figure 9]. The plot shows how to successfully identify the smallest scale values where the stability drops significantly. In our case, the sought-after ${\hat{K}}_{\text{min}}$ value, below which the identification procedure is not reliable anymore, was determined to be ${\hat{K}}_{\text{min}}\in \left[5,15\right]$ [Figure 9].

## Conclusions

The described method identifies and selects reproducible AP-MS experiments as well as bait specific preys when experimental replicates and control experiments are provided. The method is able to identify a subset of *Indicator Prey Proteins*, which enables identification of the most *Reproducible Experimental Replicates* from a larger dataset. Importantly, we show that the method uniformly scales up and down, making it quite versatile to accommodate realistic studies with a range of numbers of *Experimental Replicates*. The identification of subsets of reproducible AP-MS experiments significantly improves the ability to distinguish specific from non-specific prey proteins. In the future, this approach may be used as a general selection method for quality control purposes in proteomics studies and proteomics databases, where experimental reproducibility issues arise.

We show that at least on larger AP-MS datasets, the frequency of occurrence of prey proteins provides good sensitivity for discriminating specific from non-specific prey proteins. In our study we use the prey protein frequency as a surrogate measure for more specific protein confidence metrics such as search-engine scores [9], spectral counts [12,15] or peptide and protein probabilities [20]. Our approach may be most useful in AP-MS experimental designs incorporating sufficient number of replicates per bait protein, rather than in studies seeking to maximize the number of bait proteins analyzed at the expense of replicate AP-MS experiments.

We were also able to estimate the number of required replicate AP-MS experiments. From our analysis of stability on multiscale sets of *Experimental Replicates* [Figure 8], this number was determined to be *at least* greater than the ${\hat{K}}_{\text{min}}$ value (${\hat{K}}_{\text{min}}\in \left(5,15\right]$), at least on the data in-hand. In practice, however, the required number would best be determined by a statistical power analysis. So, this approach is useful to large replicated experiments, and especially whenever one is interested in making new discovery, where large sample size (*Experimental Replicates*) are always needed to increase statistical power and reduce false discovery rates. Lastly, since frequency is a standardized metric, our method may be useful when attempting to compare different AP-MS datasets.

The concept of *Indicator Proteins* and the related *Reproducible Experimental Replicates* rely on an objective measure of *peptide reliability*, which we chose to be the *MASCOT* peptide identification score [10]. However, clearly any other probability-based identification statistics such as e.g. the peptide probability from the peptide *PROPHET*[22] might be used. Finally, the use of *Indicator Proteins* may be applied beyond AP-MS experiments to other types of mass-spectrometry based proteomics.

## Competing interests

The authors declare that they have no competing interests.

## Authors’ contributions

J-ED and SS developed the statistical methodology and J-ED implemented the method as an R package. J-ED lead the statistical aspects of this paper, and RME lead the experimental and biological aspects. All authors formulated the problem, wrote and approved the manuscript.

## Supplementary Material

Additional file 1: Supplemental Methods Data set and Database Search. Determination of Protein Spectral Counts, Protein MASCOT Scores, and Protein Marginal Inclusion Probabilities. Raw Input Dataset Structure. Initial Pre-filtering. Derivation of Marginal and Joint Inclusion Probabilities of Indicator Prey Proteins. Identification of Reproducible Experimental Replicates and Reproducible Prey Proteins. Confidence Score and Identification of Specific Prey Proteins. Automatic Estimation of an Optimal Confidence Score Cutoff Derivation of Coefficient of Variations Formulas. Testing Stability on Multi-scale Sets of Experimental Replicates. Click here for file

Additional file 2 **Figure S1.** Scatter plots of protein spectral counts vs. protein *MASCOT* scores (left-hand-side) and protein *MASCOT* scores vs. protein marginal inclusion probabilities (right-hand-side) in all AP-MS control and bait experiments. **Figure S2**: Scatter plot of peptide *PROPHET* probabilities (*Prob*) onto the peptide *MASCOT* scores (*Score*) in all AP-MS control and bait experiments. **Figure S3**: Empirical Probability Density Function (PDF - left) and Cumulative Density Function (CDF - right) plots of peptide scores (top) and peptide probabilities (bottom) in all AP-MS control and bait experiments. **Figure S4**: Optimizing the determination of the peptide *MASCOT* score threshold in all AP-MS bait experiments. **Figure S5**: Number of *Indicator Prey Proteins* and *Reproducible Experimental Replicates* and the *joint inclusion probability* for the protein-based analysis in all AP-MS bait experiments. **Figure S6**: *FDR* sensitivity as a function of *Confidence Score* cutoff and marginal inclusion probability threshold in all AP-MS bait experiments. **Figure S7**: *FDR* and *GO* semantic similarity analyses in all AP-MS bait experiments. **Figure S8**: Density distribution plots of bait-prey *Confidence Scores* at procedural stages “*N*”, “*R*” and “*S*” in all AP-MS bait experiments. **Figure S9**: Quantile-Quantile plots of bait vs. control *marginal* inclusion probabilities in all AP-MS bait experiments. **Figure S10**: Correlation and regression relationships between protein *MASCOT* scores and protein marginal inclusion probabilities at different procedural stages from the initial “Naïve” stage (“*N*”), to the “Reproducible” stage (“*R*”), and to the final “Specific” stage (“*S*”) in all AP-MS bait experiments. **Figure S11**: Stability of the Coefficient of Variation (CV) of the mean marginal inclusion probability as a function of procedural stages from the initial “Naïve” stage (“*N*”), to the “Reproducible” stage (“*R*”), and to the final “Specific” stage (“*S*”) in all AP-MS bait experiments. **Figure S12**: Confidence Intervals (95% CIs) of the median bait-prey semantic similarity for *SAINT*-only and *SAINT* in conjunction with *ROCS* at the different *ROCS* procedural stages “*N*” (*SAINT*-only), “*R*”, and “*S*” (*ROCSSAINT*) in all AP-MS bait experiments. **Figure S13**: *FDR* computed from *SAINT* posterior probability output (*P*_*SAINT*_) as a function of procedural stages: from the initial “Naïve” stage (“*N*” – *SAINT*-only), to the “Reproducible” stage (“*R*”), and to the final “Specific” stage (“*S*” – *ROCS-SAINT*) in all AP-MS bait experiments [20,28,32,35-37]. Click here for file

Additional file 3: Table S1 *ROCS* lists of *Indicator Prey Proteins* (IPI) and *Reproducible Experimental Replicates* (RER) in all AP-MS control and bait experiments (each on a single Excel tab-sheet in a single file). Click here for file

Additional file 4: Table S2 Biological validation of ROCS protein-protein interaction (PPI) scoring results for the *Specific Prey Proteins* between *SAINT* (Posterior Probability *P*_*SAINT*_), *ComPASS* (*D* − score) and our method *ROCS* (*C* − score) in all AP-MS bait experiments (each on a separate Excel tab-sheet in a single file). Click here for file

Additional file 5: Table S3 Comparison of protein-protein interaction (PPI) scoring for the *Specific Prey Proteins* between *SAINT* (Posterior Probability *P*_*SAINT*_), *ComPASS* (*D* − score) and our method *ROCS* (*C* − score) in all AP-MS bait experiments (each on a separate Excel tab-sheet in a single file). Click here for file

Additional file 6: Table S4 Comparison of *SAINT* protein-protein interaction (PPI) scoring for the *Specific Prey Proteins* at the different *ROCS* procedural stages “*N*” (*SAINT*-only), “*R*”, and “*S*” (*ROCS*-*SAINT*) in all AP-MS bait experiments (each on a separate Excel tab-sheet in a single file). Click here for file
