# Supplementary material for: ROCS: a Reproducibility Index and Confidence Score for Interaction Proteomics Studies
Source: BMC Bioinformatics. 2012 Jun 8;13:128. doi: 10.1186/1471-2105-13-128 (PMC3568013; doi:10.1186/1471-2105-13-128)
Supplement: Additional file 1: Supplemental Methods — Data set and Database Search. Determination of Protein Spectral Counts, Protein MASCOT Scores, and Protein Marginal Inclusion Probabilities. Raw Input Dataset Structure. Initial Pre-filtering. Derivation of Marginal and Joint Inclusion Probabilities of Indicator Prey Proteins. Identification of Reproducible Experimental Replicates and Reproducible Prey Proteins. Confidence Score and Identification of Specific Prey Proteins. Automatic Estimation of an Optimal Confidence Score Cutoff Derivation of Coefficient of Variations Formulas. Testing Stability on Multi-scale Sets of Experimental Replicates. [file 1471-2105-13-128-S1.pdf]

ROCS: a Reproducibility Index and Confidence Score for Interaction Proteomics Studies

Supplemental Information

Additional file 1: Supplemental Methods

Jean-Eudes Dazard[1*£], Sudipto Saha[1*], Rob M. Ewing[1£]

* Authors with equal contribution, [£] to whom correspondence should be addressed

[1]Division of Bioinformatics, Center for Proteomics and Bioinformatics. Case Western Reserve University, 10900 Euclid Avenue, Cleveland, OH 44106, USA. Phone 216.368.3157[*£], Fax 216.368.6846[*£]

Jean-Eudes Dazard: jxd101@case.edu

Sudipto Saha: sxs881@case.edu

Rob M. Ewing: rme14@case.edu

Data set and Database Search

The five recombinant FLAG-tagged bait proteins (CTNNBIP1, STK24, VHL, NME2, PPM1B) were first expressed in human embryonic kidney 293 (HEK293) cells, the bait protein and associated proteins were then retrieved using an antibody to the FLAG epitope [20]. The extract preparations were resolved by SDS-PAGE, followed by identification of prey proteins using Ion trap mass-spectrometers (LCQ Deca, Thermo Finnigan). All spectra were re-searched against an IPI human protein sequence database (version 3.31) using the *MASCOT* mass-spectrometry search engine (version 1.9; Matrix Science, www.matrixscience.com).

Determination of Protein Spectral Counts, Protein MASCOT Scores, and Protein Marginal Inclusion Probabilities

Here we describe how spectral counts were computed for peptide and protein abundance estimations in an experimental replicate. Peptide spectral count in an experimental replicate is defined as the number of observed peptides with peptide *MASCOT* scores greater than 20. The spectral count for a protein in an experimental replicate is obtained by taking the sum of all the spectral counts of the peptides matching to that protein. Protein *MASCOT* scores are computed by taking the median across all *K Experimental Replicates* of the sums of peptide *MASCOT* scores in each experimental replicate. Protein marginal inclusion probabilities are computed by taking the frequency of occurrence of the peptides matching to that protein across all *K Experimental Replicates*, that is: $\frac{1}{K}\sum_{k=1}^{K} \mathrm{I}\left(N_j \in E_k\right)$ for $j \in \{1,\ldots,N\}$, where $N_j \in E_k$ denotes the occurrence of peptide N_j in *Experimental Replicate* E_k for $k \in \{1,\ldots,K\}$.

Raw Input Dataset Structure

Below is an example of the top 30 rows of the initial input file from the CTNNBIP1 bait AP-MS dataset. Rows are ordered by 1) Peptide Sequence, 2) IPI accession number, and 3) Experiment number (experimental replicate):

Experiment	IPI	Gene.Symbol	Peptide.Sequence	Peptide.Score	Peptide.Probability	Protein.Score	Protein.Spectral.Count
02JN07-04	IPI00385789	-	-.EDSQPMCYSNCXDGQSTAK.T	15.06	0.226	15.06	0
02JN21-07	IPI00101923	SPG11	-.M'AAEEGVASAASAGGSWGTAAMGR.V	13.34	0.1908	13.34	0
02JN27-07	IPI00654869	FLJ10324	-.M'ADLVPDLQPILFWMSNSIELLYFIQQK.C	9.88	0.1359	9.88	0
02MY30-56	IPI00178359	PMF1	-.M'AEASSANLGSGCEEK.R	13.15	0.0879	13.15	0
02JN07-04	IPI00021552	B3GALNT1	-.M'ASALWTVLPSR.M	16.08	0.1991	16.08	0
02MY30-56	IPI00028501	LRRC27	-.M'DINTYNNQLHLQR.N	19.18	0.582	19.18	0
02JN27-07	IPI00006560	SERPINB13	-.M'DSLGAVSTRLGFDLFK.E	24.84	0.5042	24.84	1
02JN20-07	IPI00303696	OR5W2	-.M'DWENCSSLTDFFLLGITNNPEM'K.V	9.65	0.0729	9.65	0
02MY30-56	IPI00179405	ZNF713	-.M'EEEEM'NDGSQM'VR.S	8.17	0.0522	8.17	0
02JN06-04	IPI00382999	-	-.M'FHSSAM'VNSHR.K	26.54	0.8415	26.54	0
02JN21-07	IPI00335849	RASAL2	-.M'FPALESDSPLPPEDLDAVVPVSGAVAGGM'LDR.I	10.56	0.1341	10.56	0
02MY30-56	IPI00443011	-	-.M'GWRSSGLQEILAYK.E	19.35	0.0644	19.35	0
02JN21-07	IPI00739364	LOC642005;LOC648911	-.M'LIFQCDECGK.A	17.71	0.0978	17.71	0
02JL05-04	IPI00449718	CTDSP1	-.M'LPCFSAAK.L	17.17	0.1187	17.17	0
02JN28-07	IPI00784455	LOC132430	-.M'NVAAKYRM'ASLYVGDLHADVTEDLLFR.K	17.88	0.0663	17.88	0
02MY30-04	IPI00457184	MT1CP	-.M'QGQEWTPIPGKFCRAGIIAGTPPTAK.A	19.35	0.139	19.35	0
02JN21-07	IPI00060423	CTHRC1	-.M'RPQGPAASPQR.L	15.29	0.1332	15.29	0
02JN07-04	IPI00300407	SDC2	-.M'RRAWILLTLGLVACVSAESR.A	25.1	0.1159	25.1	1
02MY30-56	IPI00748575	-	-.M'SCCLSSR.V	14.7	0.0586	14.7	0
02JL05-04	IPI00298058	SUPT5H	-.M'SDSEDSNFSEEEDSER.S	15.62	0.1669	15.62	0
02JN27-07	IPI00375239	-	-.M'VELVGVPRPDSGARYR.V	16.46	0.0507	16.46	0
02JN06-04	IPI00552939	C1QL3	-.M'VLLLVILIPVLVSSAGTSAHYEMLGTCR.M	18.32	0.4977	18.32	0
02JL05-04	IPI00289690	IHPK3	-.M'VVQNSADAGDMR.A	17.71	0.1137	17.71	0
02JL05-04	IPI00026904	ADSL	-.MAAGGDHGSPDSYR.S	10.92	0.0542	12.98	0
02JL05-04	IPI00026904	ADSL	-.MAAGGDHGSPDSYR.S	12.98	0.1098	12.98	0
02JN20-07	IPI00026904	ADSL	-.MAAGGDHGSPDSYR.S	13.14	0.2228	13.14	0
02JN21-07	IPI00003925	PDHB	-.MAAVSGLVR.R	13.83	0.1771	13.83	0
02MY30-56	IPI00639866	ZNF382	-.MAKPDMIRK.L	16.42	0.2666	16.42	0
02JN20-07	IPI00171599	EFHC2	-.MALPLLPGNSFNR.N	13.52	0.2369	13.52	0

A pre-processing was applied to "compile" the dataset by removing duplicated readings of the experiment entries (rows) with same experiment number (experimental replicate), same protein IPI, and same peptide sequence. Ties are broken by taking the experiment entry with peptide having the highest probability score. In this example dataset, the initial number of experiment entries was 2639, with 1734 unique prey peptide sequences, and $N^B = 1229$ uniquely identified corresponding prey proteins.

Initial Pre-filtering

One may initially remove the family of keratin proteins from the datasets if these proteins are not expressed in the experimental parent cell line (e.g. in HEK293), since in this case these proteins are merely the result of human contamination at the experimental level. After cleaning-up the datasets, we regressed the peptide probabilities (abbreviated *Prob*), onto the peptide *MASCOT* scores (abbreviated *Score*), by using a non-linear (cubic smoothing B-splines) quantile regression approach. We first determine the peptide score threshold, termed *MASCOT Score Threshold* (*MST*), corresponding to the αth-quantile of peptide probabilities, termed *Peptide Probability Threshold*, and denoted $Prob^{(\alpha)}$ (or α, since $Prob^{(\alpha)} = \alpha$ by definition) from the estimated median regression function, formally: $MST = f_{MR}^{-1}\left(Prob^{(\alpha)}\right) = f_{MR}^{-1}(\alpha)$ where $f_{MR}^{-1}(.)$ denotes the inverse of the Median Regression (*MR*) function of the B-spline model. Let's consider the subset

of uniquely identified *Prey Proteins* termed *Prefiltered Prey Proteins* for which their corresponding peptide scores are greater than the *MASCOT Score Threshold*. We denote it by $\left\{P_1,\ldots,P_P\right\}=\left\{N_j,\, j\in\left\{1,\ldots,N\right\}: Score(N_j)\geq MST\right\}$, and its cardinal set by $P=\left|\left\{P_1,\ldots,P_P\right\}\right|$.

Derivation of Marginal and Joint Inclusion Probabilities of Indicator Prey Proteins

The subset of uniquely identified *Indicator Prey Proteins* is by definition given by $\left\{Q_1,\ldots,Q_Q\right\}=\left\{P_j,\, j\in\left\{1,\ldots,P\right\}: Score(P_j)\geq RIT\geq MST\right\}$, of cardinal set $Q=\left|\left\{Q_1,\ldots,Q_Q\right\}\right|$. We define for each *Indicator Prey Protein* its *marginal* inclusion probability across all *Experimental Replicates* as $p_{\mathrm{M}}(j)=\Pr\left(Q_j\in\left\{E_1,\ldots,E_K\right\}\right)$ for $j\in\left\{1,\ldots,Q\right\}$, where $Q_j\in\left\{E_1,\ldots,E_K\right\}$ denotes the occurrence of protein Q_j in any *Experimental Replicate* E_k, for $k\in\left\{1,\ldots,K\right\}$. This probability is estimated by the *marginal* frequency of occurrence: $\hat{p}_{\mathrm{M}}(j)=\dfrac{1}{K}\sum_{k=1}^{K}\mathrm{I}\left(Q_j\in E_k\right)$ for $j\in\left\{1,\ldots,Q\right\}$, where $Q_j\in E_k$ denotes the occurrence of protein Q_j in *Experimental Replicate* E_k for $k\in\left\{1,\ldots,K\right\}$. For any given marginal inclusion probability threshold $\tilde{p}_{\min}$, one may define a subset of *Indicator Prey Proteins* for which their *marginal* inclusion probability is greater than $\tilde{p}_{\min}$. Hereafter, since the cardinal set of such prey *Indicator Prey Proteins* depends on $\tilde{p}_{\min}$, we denote this subset by $\left\{Q_1,\ldots,Q_{Q(\tilde{p}_{\min})}\right\}$ and its cardinal by $Q(\tilde{p}_{\min})=\left|\left\{Q_1,\ldots,Q_{Q(\tilde{p}_{\min})}\right\}\right|$. Then, one may define the *joint* inclusion probability of *Indicator Prey Proteins* $\left\{Q_1,\ldots,Q_{Q(\tilde{p}_{\min})}\right\}$ across all *Experimental Replicates* $\left\{E_1,\ldots,E_K\right\}$ as $p_{\mathrm{J}}(\tilde{p}_{\min})=\Pr\left(\bigcap_{j=1}^{Q(\tilde{p}_{\min})}\left(Q_j\in\left\{E_1,\ldots,E_K\right\}\right)\right)$. The latter is estimated, assuming independence, by the *joint* frequency of occurrences of all these *Indicator Prey Proteins* $\left\{Q_1,\ldots,Q_{Q(\tilde{p}_{\min})}\right\}$ across all *Experimental Replicates* $\left\{E_1,\ldots,E_K\right\}$:

$$\hat{p}_{\mathrm{J}}(\tilde{p}_{\min})=\frac{1}{K^{Q(\tilde{p}_{\min})}}\prod_{j=1}^{Q(\tilde{p}_{\min})}\sum_{k=1}^{K}\mathrm{I}\left(Q_j\in E_k\right)\qquad\text{for }\tilde{p}_{\min}\in\left[0,1\right]$$

Therefore, by fixing a marginal inclusion probability threshold $\tilde{p}_{\min}$, a subset of highly reproducible *Indicator Prey Proteins* can be identified for which their *marginal* inclusion probability is greater than the $\tilde{p}_{\min}$ threshold and their *joint* inclusion probability is high.

Identification of Reproducible Experimental Replicates and Reproducible Prey Proteins

Since the subset of *Reproducible Experimental Replicates* depends on the marginal inclusion probability threshold $\tilde{p}_{\min}$, it is fully denoted by $\left\{F_1,\ldots,F_{L(\tilde{p}_{\min})}\right\}$, and its cardinal set by $L(\tilde{p}_{\min}) = \left|\left\{F_1,\ldots,F_{L(\tilde{p}_{\min})}\right\}\right|$, where obviously $\left\{F_1,\ldots,F_{L(\tilde{p}_{\min})}\right\} \subseteq \{E_1,\ldots,E_K\}$ and $L(\tilde{p}_{\min}) \le K$. For a given $\tilde{p}_{\min}$, this cardinal can be estimated as:

$$\hat{L}(\tilde{p}_{\min}) = \sum_{k=1}^{K} \mathrm{I}\left(\left\{Q_1,\ldots,Q_{Q(\tilde{p}_{\min})}\right\} \in E_k\right) \qquad \text{for } \tilde{p}_{\min} \in [0,1]$$

Likewise, the reduced set of *Reproducible Experimental Replicates* is fully denoted by $\left\{F_1,\ldots,F_{L(\tilde{p}_{\min})}\right\}$, and the corresponding set of uniquely identified *Reproducible Prey Proteins* by $\left\{R_1,\ldots,R_{R(\tilde{p}_{\min})}\right\} = \left\{P_j,\, j \in \{1,\ldots,P\} : P_j \in \left\{F_1,\ldots,F_{L(\tilde{p}_{\min})}\right\}\right\}$, of cardinal set $R(\tilde{p}_{\min}) = \left|\left\{R_1,\ldots,R_{R(\tilde{p}_{\min})}\right\}\right|$.

Confidence Score and Identification of Specific Prey Proteins

Using previous notations, the *marginal* inclusion probability for each *Reproducible Prey Protein* is fully denoted in both bait and control experiments as $p'^{B}_{\mathrm{M}}(j, \tilde{p}^{B}_{\min}) = \Pr\left(R^{B}_{j} \in \left\{F^{B}_{1},\ldots,F^{B}_{L^{B}(\tilde{p}^{B}_{\min})}\right\}\right)$ for $j \in \left\{1,\ldots,R^{B}(\tilde{p}^{B}_{\min})\right\}$ and $p'^{C}_{\mathrm{M}}(j, \tilde{p}^{C}_{\min}) = \Pr\left(R^{C}_{j} \in \left\{F^{C}_{1},\ldots,F^{C}_{L^{C}(\tilde{p}^{C}_{\min})}\right\}\right)$ for $j \in \left\{1,\ldots,R^{C}(\tilde{p}^{C}_{\min})\right\}$. They are estimated by the *marginal* frequencies of occurrences of *Reproducible Prey Proteins* $\left\{R^{B}_{1},\ldots,R^{B}_{R^{B}(\tilde{p}^{B}_{\min})}\right\}$ and $\left\{R^{C}_{1},\ldots,R^{C}_{R^{C}(\tilde{p}^{C}_{\min})}\right\}$ across all $L^{B}(p^{B}_{\min})$ and $L^{C}(p^{C}_{\min})$ *Reproducible Experimental Replicates*:

$$\begin{cases} \hat{p}'^{B}_{\mathrm{M}}(j, \tilde{p}^{B}_{\min}) = \dfrac{1}{L^{B}(\tilde{p}^{B}_{\min})} \displaystyle\sum_{k=1}^{L^{B}(\tilde{p}^{B}_{\min})} \mathrm{I}\left(R^{B}_{j} \in F^{B}_{k}\right) \\ \hat{p}'^{C}_{\mathrm{M}}(j, \tilde{p}^{C}_{\min}) = \dfrac{1}{L^{C}(\tilde{p}^{C}_{\min})} \displaystyle\sum_{k=1}^{L^{C}(\tilde{p}^{C}_{\min})} \mathrm{I}\left(R^{C}_{j} \in F^{C}_{k}\right) \end{cases} \qquad \text{for } \begin{cases} j \in \left\{1,\ldots,R^{B}(\tilde{p}^{B}_{\min})\right\} & \tilde{p}^{B}_{\min} \in [0,1] \\ j \in \left\{1,\ldots,R^{C}(\tilde{p}^{C}_{\min})\right\} & \tilde{p}^{C}_{\min} \in [0,1] \end{cases}$$

Also, the *Confidence Score* for the *j*-th prey protein in $\left\{R_1^B,\ldots,R_{R^B}^B\right\}$, and for fixed $\tilde{p}_{\min}^B$ and $\tilde{p}_{\min}^C$, is fully denoted as follows:

$$C_S(j,\tilde{p}_{\min}^B,\tilde{p}_{\min}^C)=\frac{\hat{p}_{\mathrm{M}}'^B(j,\tilde{p}_{\min}^B)-\hat{p}_{\mathrm{M}}'^C(j,\tilde{p}_{\min}^C)}{\hat{p}_{\mathrm{M}}'^B(j,\tilde{p}_{\min}^B)+\hat{p}_{\mathrm{M}}'^C(j,\tilde{p}_{\min}^C)}\cdot\hat{p}_{\mathrm{M}}'^B(j,\tilde{p}_{\min}^B)\qquad \text{for } j\in\left\{1,\ldots,R^B(\tilde{p}_{\min}^B)\right\}$$

Automatic Estimation of an Optimal Confidence Score Cutoff

Regarding the estimation of the optimal *Confidence Score* cutoff $\left(C_S^{cutoff}\right)$, since both of the False Positive $\hat{FP}\left(C_S^{cutoff}\right)$ and True Positive $\hat{TP}\left(C_S^{cutoff}\right)$ estimates of the number of identified bait-prey Protein-Protein-Interaction (PPI) depend on it, the estimated *FDR* is fully notated with a dependency to it, that is: $F\hat{D}R\left(C_S^{cutoff}\right)$. Likewise, the corresponding subset of *Specific Prey Proteins*, which depends on C_S^{cutoff} as well as the marginal inclusion probability thresholds $\tilde{p}_{\min}^B$ and $\tilde{p}_{\min}^C$, is fully denoted with respect to dependencies C_S^{cutoff}, $\tilde{p}_{\min}^B$ and $\tilde{p}_{\min}^C$ by $\left\{S_1^B,\ldots,S_{S^B(\tilde{p}_{\min}^B,\tilde{p}_{\min}^C,C_S^{cutoff})}^B\right\}$ of cardinal set $S^B(\tilde{p}_{\min}^B,\tilde{p}_{\min}^C,C_S^{cutoff})=\left|\left\{S_1^B,\ldots,S_{S^B(\tilde{p}_{\min}^B,\tilde{p}_{\min}^C,C_S^{cutoff})}^B\right\}\right|$, and defined as:

$$\left\{S_1^B,\ldots,S_{S^B(\tilde{p}_{\min}^B,\tilde{p}_{\min}^C,C_S^{cutoff})}^B\right\}=\left\{R_j^B,\ j\in\left\{1,\ldots,R^B(\tilde{p}_{\min}^B)\right\}:C_S(j,\tilde{p}_{\min}^B,\tilde{p}_{\min}^C)\geq C_S^{cutoff}\right\}$$

The $\hat{FP}$ estimate of the number of identified bait-prey Protein-Protein-Interaction (PPI) is computed by applying the entire ROCS identification procedure to B_1 repeated random samples (without replacement) of size N^B of prey proteins identified from the (stage "*N*") of control experiments. The $\hat{FP}$ estimate is computed as the average number of identified bait-prey PPI above the *Confidence Score* cutoff $\left(C_S^{cutoff}\right)$ expected in the Monte-Carlo replicates: $\hat{FP}\left(C_S^{cutoff}\right)=\frac{1}{B_1}\sum_{b=1}^{B_1}\hat{S}^{B(*b)}(\tilde{p}_{\min}^B,\tilde{p}_{\min}^C,C_S^{cutoff})$ where each $\hat{S}^{B(*b)}(\tilde{p}_{\min}^B,\tilde{p}_{\min}^C,C_S^{cutoff})$ denotes a Monte-Carlo cardinal set of control *Specific Prey Proteins* for $b\in\left\{1,\ldots,B_1\right\}$. Finally, the estimated *FDR* can be computed as:

$$F\hat{D}R\left(C_S^{cutoff}\right)=\frac{\frac{1}{B_1}\sum_{b=1}^{B_1}\hat{S}^{B(*b)}(\tilde{p}_{\min}^B,\tilde{p}_{\min}^C,C_S^{cutoff})}{S^B(\tilde{p}_{\min}^B,\tilde{p}_{\min}^C,C_S^{cutoff})}\qquad \text{for } C_S^{cutoff}\in\left(0,1\right]$$

Finally, the pairwise "Resnik" measure of semantic similarity computed between two *GO* terms within a given ontology [28] also depends on the *Confidence Score* cutoff $\left(C_S^{cutoff}\right)$. Therefore, the distance between the Confidence Intervals (CIs) of the medians, computed as the difference between the lower bound of the $100(1-\theta)\%$ CI from the "*R*" stage and the upper bound of the $100(1-\theta)\%$ CI from the "*N*" stage is then as a function of the *Confidence Score* cutoff $\left(C_S^{cutoff}\right)$, and is fully denoted by $d\left(C_S^{cutoff}\right)=LB\left[sim_{C_S^{cutoff}}^R\left(c_{\mathrm{B}},c_{\mathrm{P}}\right)\right]-UB\left[sim_{C_S^{cutoff}}^N\left(c_{\mathrm{B}},c_{\mathrm{P}}\right)\right]$

Derivation of the Coefficient of Variations Formulas

For the comparison of performances between procedural stages ("Naïve" stage ("*N*"), "Reproducible" stage ("*R*"), and final "Specific" stage ("*S*")), we computed the *marginal* inclusion probability for each selected prey protein from each of these subsets across the corresponding number of *Experimental Replicates*, similarly to (6):

$$\begin{cases}\hat{p}_{\mathrm{M}}^B(j)=\frac{1}{K^B}\sum_{k=1}^{K^B}\mathrm{I}\left(N_j^B\in E_k^B\right)\\ \hat{p}_{\mathrm{M}}'^B(j,\tilde{p}_{\min}^B)=\frac{1}{L^B(\tilde{p}_{\min}^B)}\sum_{k=1}^{L^B(\tilde{p}_{\min}^B)}\mathrm{I}\left(R_j^B\in F_k^B\right)\\ \hat{p}_{\mathrm{M}}''^B(j,\tilde{p}_{\min}^B,\tilde{p}_{\min}^C,C_S^{cutoff})=\frac{1}{L^B(\tilde{p}_{\min}^B)}\sum_{k=1}^{L^B(\tilde{p}_{\min}^B)}\mathrm{I}\left(R_j^B\in F_k^B\right)\end{cases}$$

$$\text{for}\quad\begin{cases}j\in\left\{1,\ldots,N^B\right\}\\ j\in\left\{1,\ldots,R^B(\tilde{p}_{\min}^B)\right\} & \tilde{p}_{\min}^B\in\left[0,1\right]\\ j\in\left\{1,\ldots,S^B(\tilde{p}_{\min}^B,\tilde{p}_{\min}^C,C_S^{cutoff})\right\} & \tilde{p}_{\min}^B\in\left[0,1\right],\tilde{p}_{\min}^C\in\left[0,1\right],C_S^{cutoff}\in\left[-1,1\right]\end{cases}$$

Then, to assess reproducibility in a bait experiment, we compared the overall Coefficient of Variations (CV) of the average number of *marginal* inclusion probabilities of the selected prey proteins across *Experimental Replicates*. This was carried out from the "Naïve" stage ("*N*"), to the "Reproducible" stage ("*R*"), and to the final "Specific" stage ("*S*") as follows:

$$\begin{cases} \overline{p}_{\mathrm{M}}^{B} = \dfrac{1}{N^{B}} \displaystyle\sum_{j=1}^{N^{B}} \hat{p}_{\mathrm{M}}^{B}(j) \\ \overline{p}_{\mathrm{M}}'^{B}(\tilde{p}_{\min}^{B}) = \dfrac{1}{R^{B}(\tilde{p}_{\min}^{B})} \displaystyle\sum_{j=1}^{R^{B}(\tilde{p}_{\min}^{B})} \hat{p}_{\mathrm{M}}'^{B}(j, \tilde{p}_{\min}^{B}) \\ \overline{p}_{\mathrm{M}}''^{B}(\tilde{p}_{\min}^{B}, \tilde{p}_{\min}^{C}, C_{S}^{cutoff}) = \dfrac{1}{S^{B}(\tilde{p}_{\min}^{B}, \tilde{p}_{\min}^{C}, C_{S}^{cutoff})} \displaystyle\sum_{j=1}^{S^{B}(\tilde{p}_{\min}^{B}, \tilde{p}_{\min}^{C}, C_{S}^{cutoff})} \hat{p}_{\mathrm{M}}''^{B}(j, \tilde{p}_{\min}^{B}, \tilde{p}_{\min}^{C}, C_{S}^{cutoff}) \end{cases}$$

And unbiased estimates of the standard deviations are given by:

$$\begin{cases} \hat{S}D^{B} = \dfrac{1}{N^{B}-1} \displaystyle\sum_{j=1}^{N^{B}} \left(\hat{p}_{\mathrm{M}}^{B}(j) - \overline{p}_{\mathrm{M}}^{B} \right)^{2} \\ \hat{S}D'^{B}(\tilde{p}_{\min}^{B}) = \dfrac{1}{R^{B}(\tilde{p}_{\min}^{B})-1} \displaystyle\sum_{j=1}^{R^{B}(\tilde{p}_{\min}^{B})} \left(\hat{p}_{\mathrm{M}}'^{B}(j, \tilde{p}_{\min}^{B}) - \overline{p}_{\mathrm{M}}'^{B}(\tilde{p}_{\min}^{B}) \right)^{2} \\ \hat{S}D''^{B}(\tilde{p}_{\min}^{B}, \tilde{p}_{\min}^{C}, C_{S}^{cutoff}) = \dfrac{\displaystyle\sum_{j=1}^{S^{B}(\tilde{p}_{\min}^{B}, \tilde{p}_{\min}^{C}, C_{S}^{cutoff})} \left(\hat{p}_{\mathrm{M}}''^{B}(j, \tilde{p}_{\min}^{B}, \tilde{p}_{\min}^{C}, C_{S}^{cutoff}) - \overline{p}_{\mathrm{M}}''^{B}(\tilde{p}_{\min}^{B}, \tilde{p}_{\min}^{C}, C_{S}^{cutoff}) \right)^{2}}{S^{B}(\tilde{p}_{\min}^{B}, \tilde{p}_{\min}^{C}, C_{S}^{cutoff})-1} \end{cases}$$

Finally, the Coefficients of variations are:

$$\begin{cases} \hat{C}V^{B} = \dfrac{\hat{S}D^{B}}{\overline{p}_{\mathrm{M}}^{B}} \\ \hat{C}V'^{B}(\tilde{p}_{\min}^{B}) = \dfrac{\hat{S}D'^{B}(\tilde{p}_{\min}^{B})}{\overline{p}_{\mathrm{M}}'^{B}(\tilde{p}_{\min}^{B})} \\ \hat{C}V''^{B}(\tilde{p}_{\min}^{B}, \tilde{p}_{\min}^{C}, C_{S}^{cutoff}) = \dfrac{\hat{S}D''^{B}(\tilde{p}_{\min}^{B}, \tilde{p}_{\min}^{C}, C_{S}^{cutoff})}{\overline{p}_{\mathrm{M}}''^{B}(\tilde{p}_{\min}^{B}, \tilde{p}_{\min}^{C}, C_{S}^{cutoff})} \end{cases}$$

$$\text{for} \quad \begin{cases} j \in \left\{1, \ldots, N^{B}\right\} & \\ j \in \left\{1, \ldots, R^{B}(\tilde{p}_{\min}^{B})\right\} & \tilde{p}_{\min}^{B} \in [0,1] \\ j \in \left\{1, \ldots, S^{B}(\tilde{p}_{\min}^{B}, \tilde{p}_{\min}^{C}, C_{S}^{cutoff})\right\} & \tilde{p}_{\min}^{B} \in [0,1], \tilde{p}_{\min}^{C} \in [0,1], C_{S}^{cutoff} \in [-1,1] \end{cases}$$

Testing Stability on Multi-scale Sets of Experimental Replicates

The goal is to get the *joint inclusion probability* $p_{\mathrm{J}}(k, \tilde{p}_{\min})$ of *Indicator Prey Proteins* (for which their *marginal* inclusion probability is greater than a given threshold $\tilde{p}_{\min}$), computed across all *Experimental Replicates* $\{E_1, \ldots, E_k\}$, where $k \in [3, K]$ is the experimental scale. In the following, the maximum experimental scale (K) and the marginal inclusion probability threshold ($\tilde{p}_{\min}$) are supposed to be fixed, so we further dropped their dependencies throughout the following formal definitions. We first randomly subset $k \in [3, K]$ *Experimental Replicates* $\{E_1^*, \ldots, E_k^*\}$ from the original data $\{E_1, \ldots, E_K\}$ by sampling *without replacement* for each subset $\{E_1^*, \ldots, E_k^*\}$ (where $k \in [3, K]$), we generated B_1 *multiscale* bootstrapped subsets of *Experimental Replicates* by randomly sampling B_1 times *with replacement* from the initial subset $\{E_1^*, \ldots, E_k^*\}$ of *Experimental Replicates*. We denote these by $\{E_1^{*1}, \ldots, E_{k^{*1}}^{*1}\}, \cdots, \{E_1^{*b}, \ldots, E_{k^{*b}}^{*b}\}, \cdots, \{E_1^{*B_1}, \ldots, E_{k^{*B_1}}^{*B_1}\}$. Then, the entire identification procedure is applied to each bootstrapped subset $\{E_1^{*b}, \ldots, E_{k^{*b}}^{*b}\}$, giving for each $b \in \{1, \ldots, B_1\}$ the number of bootstrapped *Reproducible Experimental Replicates* $\hat{L}^{*b}(k)$ and the corresponding bootstrapped joint inclusion probability $\hat{p}_{\mathrm{J}}^{*b}(k)$ for each $k \in [3, K]$.

There are two types of so-called "*multiscale*" estimates that can be derived from these quantities to appropriately measure the stability of the performance of the procedure as a function of the experimental scale k. One is a so-called *multiscale mean joint inclusion probability* estimated by $\hat{p}_{\mathrm{MJ}}(k) = \frac{1}{B_1} \sum_{b=1}^{B_1} \hat{p}_{\mathrm{J}}^{*b}(k)$. However, since this estimate was shown to be biased [35-37], we derived a so-called *multiscale unbiased joint inclusion probability* estimate from the multiscale bootstrapping procedure mentioned above. Specifically, we looked at changes in the $\hat{z}^{*b}(k^{*b}) = -\Phi^{-1}\left(\hat{p}_J^{*b}(k^{*b})\right)$ values for $b \in \{1, \ldots, B_1\}$, where $\Phi^{-1}(.)$ denotes the inverse cumulative distribution function of the standard normal distribution. We denote by $r^{*b}(k^{*b}) = \sqrt{k^{*b}/k}$ a normalized measure of the experimental scale ratio, then the theoretical

curve $\hat{z}^{*b}(k^{*b}) = \nu \cdot r(k^{*b}) + \lambda \cdot \frac{1}{r(k^{*b})}$ is fitted using nonlinear least-squares estimation to the observed values, and the coefficients are estimated, denoted $\left\{\hat{\nu}(k^{*b}), \hat{\lambda}(k^{*b})\right\}$, for each value of k^{*b}, $b \in \{1, \ldots, B_1\}$. The *multiscale unbiased joint inclusion probability* is then given by $\hat{p}_{\mathrm{UJ}}(k) = \Phi\left(\hat{\lambda}(k^{*b}) - \hat{\nu}(k^{*b})\right)$. Finally, the entire procedure is repeated B_2 times to get the corresponding mean and standard error estimates $\overline{p}_{\mathrm{MJ}}(k)$ and $se(\overline{p}_{\mathrm{MJ}})(k)$, as well as $\overline{p}_{\mathrm{UJ}}(k)$ and $se(\overline{p}_{\mathrm{UJ}})(k)$, simply by taking the average over the B_2 replicates.
