# Supplementary material for: ROCS: a Reproducibility Index and Confidence Score for Interaction Proteomics Studies
Source: BMC Bioinformatics. 2012 Jun 8;13:128. doi: 10.1186/1471-2105-13-128 (PMC3568013; doi:10.1186/1471-2105-13-128)
Supplement: Additional file 2 — Figure S1. Scatter plots of protein spectral counts vs. protein MASCOT scores (left-hand-side) and protein MASCOT scores vs. protein marginal inclusion probabilities (right-hand-side) in all AP-MS control and bait experiments. Figure S2: Scatter plot of peptide PROPHET probabilities (Prob) onto the peptide MASCOT scores (Score) in all AP-MS control and bait experiments. Figure S3: Empirical Probability Density Function (PDF - left) and Cumulative Density Function (CDF - right) plots of peptide scores (top) and peptide probabilities (bottom) in all AP-MS control and bait experiments. Figure S4: Optimizing the determination of the peptide MASCOT score threshold in all AP-MS bait experiments. Figure S5: Number of Indicator Prey Proteins and Reproducible Experimental Replicates and the joint inclusion probability for the protein-based analysis in all AP-MS bait experiments. Figure S6: FDR sensitivity as a function of Confidence Score cutoff and marginal inclusion probability threshold in all AP-MS bait experiments. Figure S7: FDR and GO semantic similarity analyses in all AP-MS bait experiments. Figure S8: Density distribution plots of bait-prey Confidence Scores at procedural stages “N”, “R” and “S” in all AP-MS bait experiments. Figure S9: Quantile-Quantile plots of bait vs. control marginal inclusion probabilities in all AP-MS bait experiments. Figure S10: Correlation and regression relationships between protein MASCOT scores and protein marginal inclusion probabilities at different procedural stages from the initial “Naïve” stage (“N”), to the “Reproducible” stage (“R”), and to the final “Specific” stage (“S”) in all AP-MS bait experiments. Figure S11: Stability of the Coefficient of Variation (CV) of the mean marginal inclusion probability as a function of procedural stages from the initial “Naïve” stage (“N”), to the “Reproducible” stage (“R”), and to the final “Specific” stage (“S”) in all AP-MS bait experiments. Figure S12: Confidence Intervals (95% CIs) of the [file 1471-2105-13-128-S2.pdf]

# *ROCS*: a Reproducibility Index and Confidence Score for Interaction Proteomics Studies

# Supplemental Information

## Additional file 2: Supplemental Figures and Tables


Jean-Eudes Dazard[1*£], Sudipto Saha[1*], Rob M. Ewing[1£]

* Authors with equal contribution, [£] to whom correspondence should be addressed

[1]Division of Bioinformatics, Center for Proteomics and Bioinformatics. Case Western Reserve University, 10900 Euclid Avenue, Cleveland, OH 44106, USA. Phone 216.368.3157[*£], Fax 216.368.6846[*£]

Jean-Eudes Dazard: jxd101@case.edu

Sudipto Saha: sxs881@case.edu

Rob M. Ewing: rme14@case.edu

**Additional file 2: Figure S1:** Scatter plots of protein spectral counts vs. protein *MASCOT* scores (left-hand-side) and protein *MASCOT* scores vs. protein marginal inclusion probabilities (right-hand-side) in the AP-MS control (CONTROL) and all bait experiments (CTNNBIP1, STK24, NME2, PPM1B, and VHL). See above Additional file 1 Supplemental Methods for more details on how protein spectral counts, protein *MASCOT* scores and protein marginal inclusion probabilities were computed [Additional file 1: Supplemental Methods]. Note how the coefficients of determination and of correlation are systematically lower in the scatter plots of protein spectral counts vs. protein *MASCOT* scores (left-hand-side) than in the scatter plots protein *MASCOT* scores vs. protein marginal inclusion probabilities (right-hand-side). Also, note in the right-hand-side scatter plots how many proteins with low marginal inclusion probabilities have relatively high *MASCOT* scores.

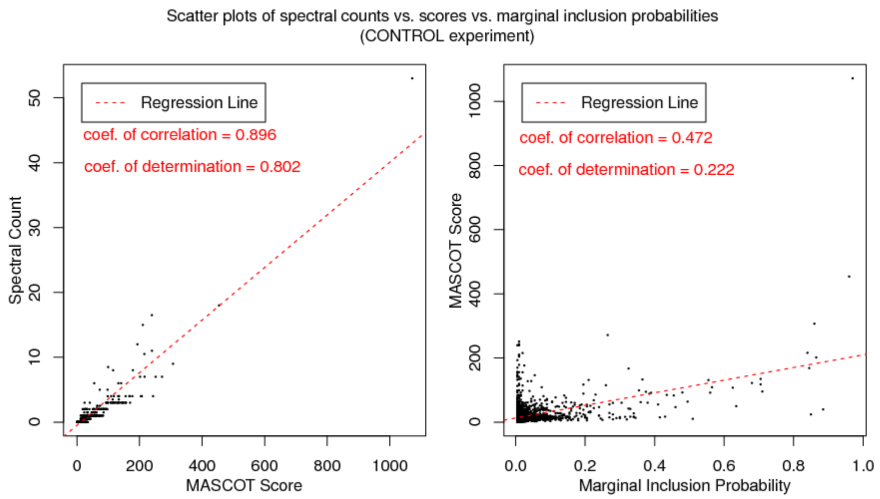

Scatter plots of spectral counts vs. scores vs. marginal inclusion probabilities
(CTNNBIP1 experiment)

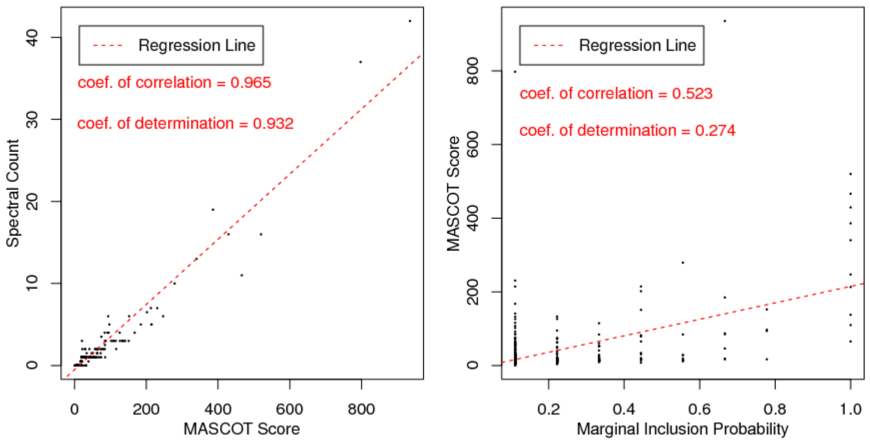


Scatter plots of spectral counts vs. scores vs. marginal inclusion probabilities
(STK24 experiment)

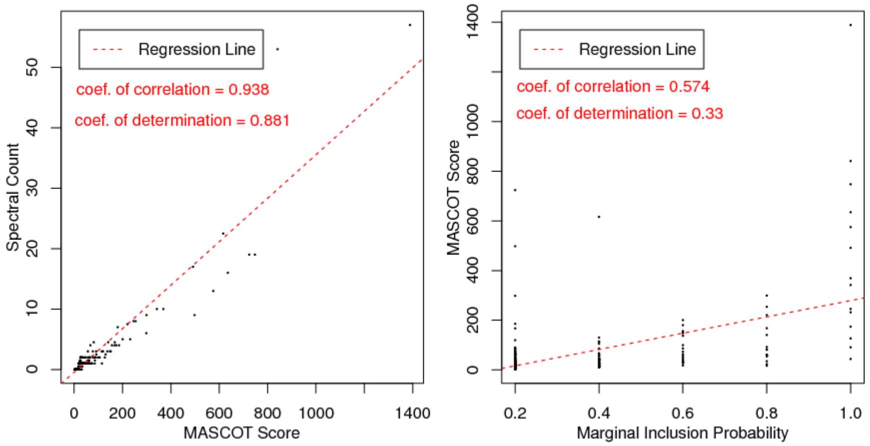


Scatter plots of spectral counts vs. scores vs. marginal inclusion probabilities
(NME2 experiment)

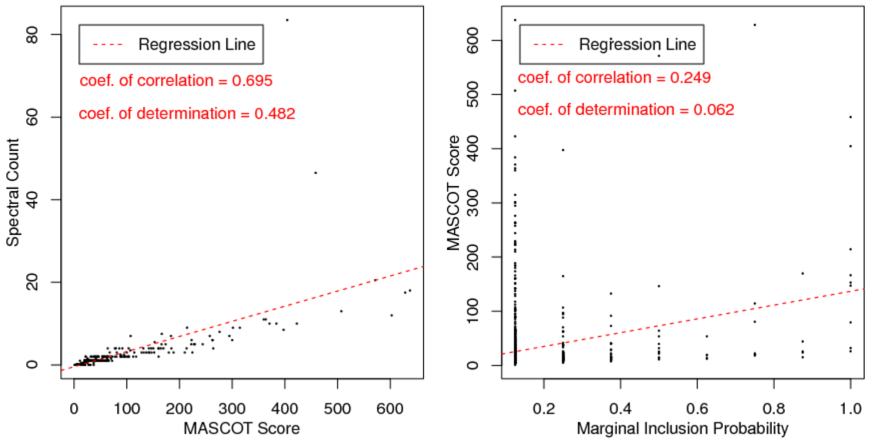

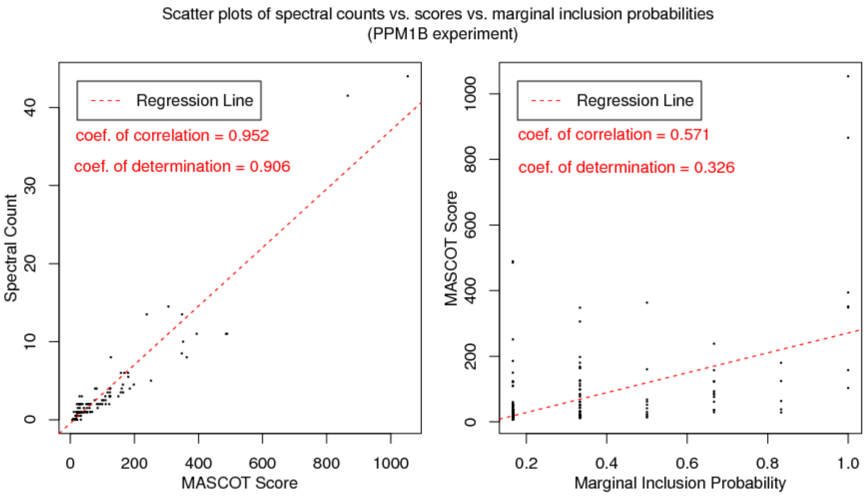
Scatter plots of spectral counts vs. scores vs. marginal inclusion probabilities
(PPM1B experiment)
Regression Line
coef. of correlation = 0.952
coef. of determination = 0.906
Spectral Count
MASCOT Score
Regression Line
coef. of correlation = 0.571
coef. of determination = 0.326
MASCOT Score
Marginal Inclusion Probability

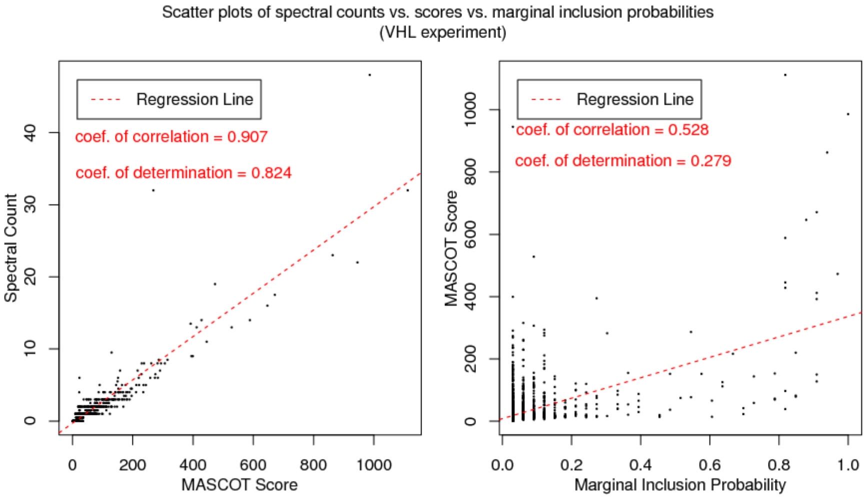
Scatter plots of spectral counts vs. scores vs. marginal inclusion probabilities
(VHL experiment)
Regression Line
coef. of correlation = 0.907
coef. of determination = 0.824
Spectral Count
MASCOT Score
Regression Line
coef. of correlation = 0.528
coef. of determination = 0.279
MASCOT Score
Marginal Inclusion Probability

**Additional file 2: Figure S2**: Scatter plot of peptide *PROPHET* probabilities (*Prob*) onto the peptide *MASCOT* scores (*Score*) in the AP-MS control (CONTROL) and all bait experiments (CTNNBIP1, STK24, NME2, PPM1B, and VHL). Non Linear Quartile Regression lines are shown with *MASCOT Score Threshold* (*MST*), and corresponding *Peptide Probability Threshold* $\left(Prob^{(0.5)} = 0.5\right)$.

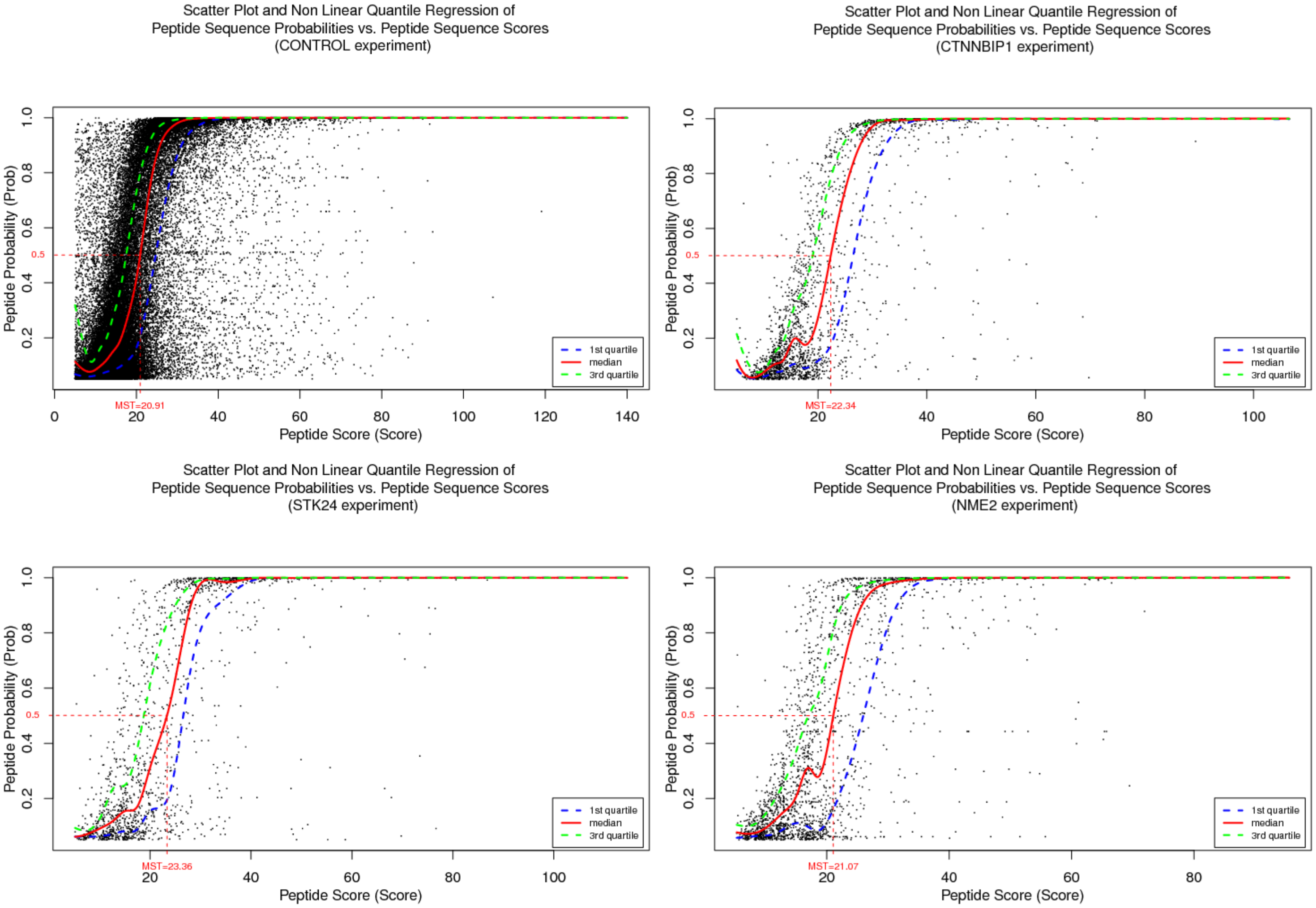

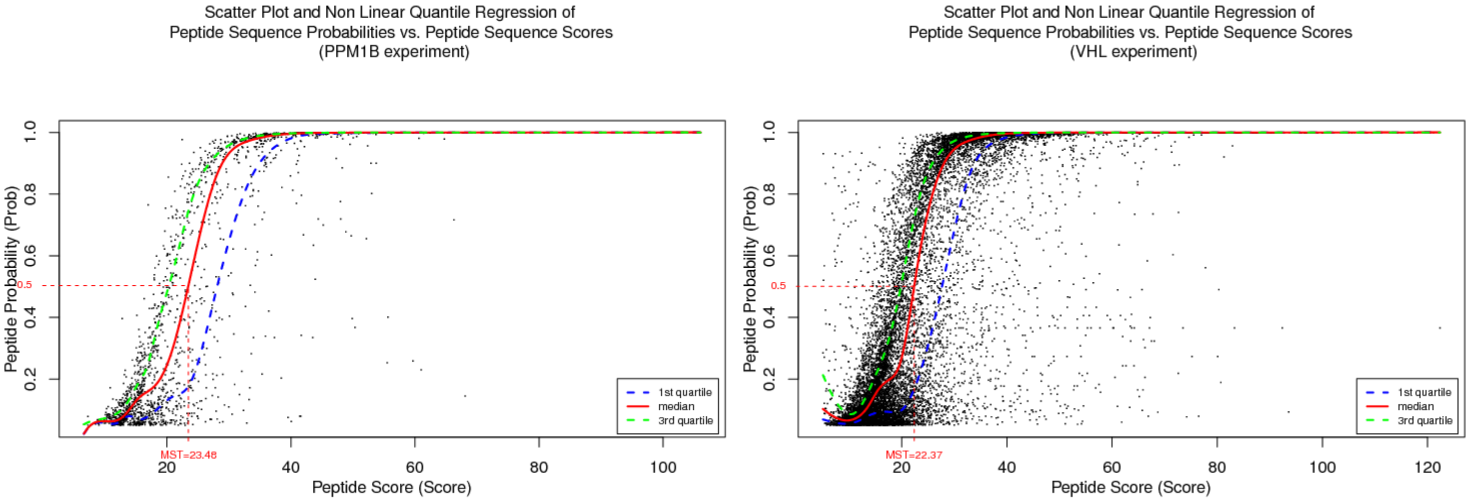

Scatter Plot and Non Linear Quantile Regression of
Peptide Sequence Probabilities vs. Peptide Sequence Scores
(PPM1B experiment)
Peptide Probability (Prob)
Peptide Score (Score)
0.5
MST=23.48
1st quartile
median
3rd quartile
Scatter Plot and Non Linear Quantile Regression of
Peptide Sequence Probabilities vs. Peptide Sequence Scores
(VHL experiment)
Peptide Probability (Prob)
Peptide Score (Score)
0.5
MST=22.37
1st quartile
median
3rd quartile

**Additional file 2: Figure S3**: Empirical Probability Density Function (PDF - left) and Cumulative Density Function (CDF - right) plots of peptide scores (top) and peptide probabilities (bottom) in the AP-MS control (CONTROL) and all bait experiments (CTNNBIP1, STK24, NME2, PPM1B, and VHL). The *MASCOT Score Threshold* (*MST*) is shown in red with its corresponding *Peptide Probability Threshold* $\alpha = 0.5$.

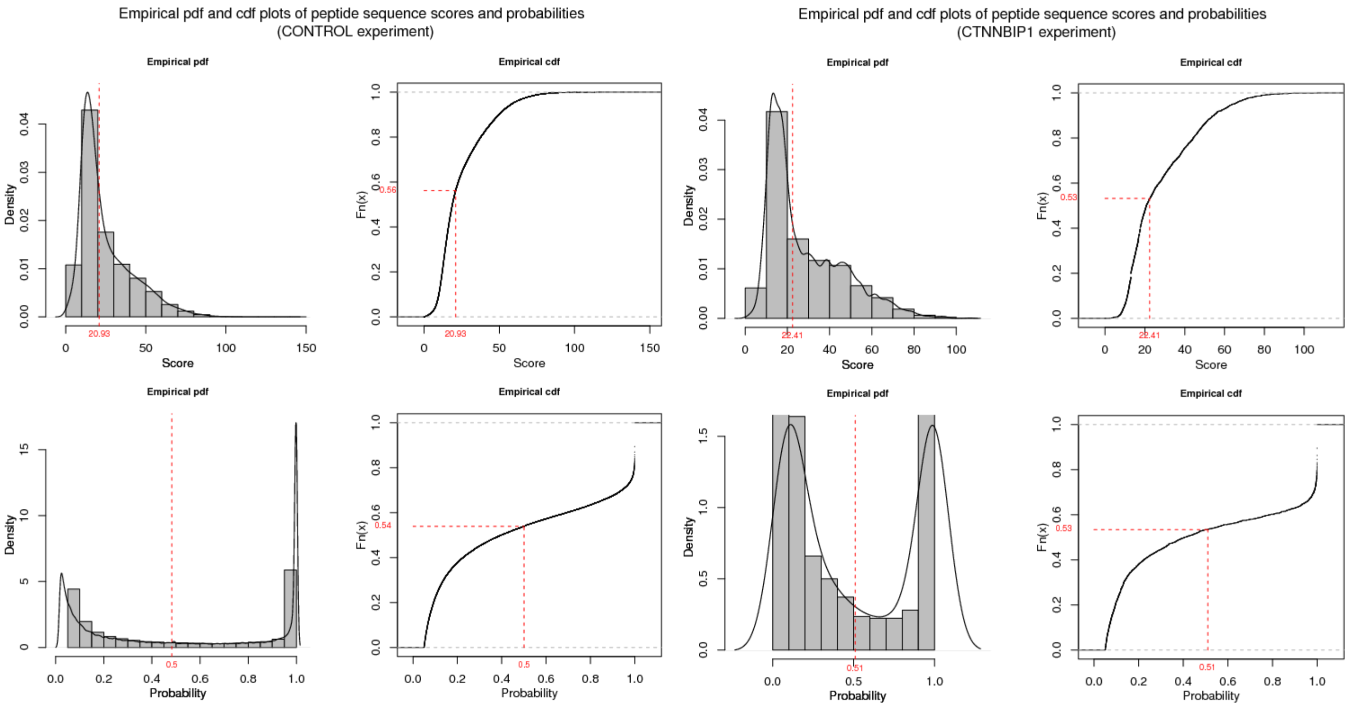

Empirical pdf and cdf plots of peptide sequence scores and probabilities
(STK24 experiment)

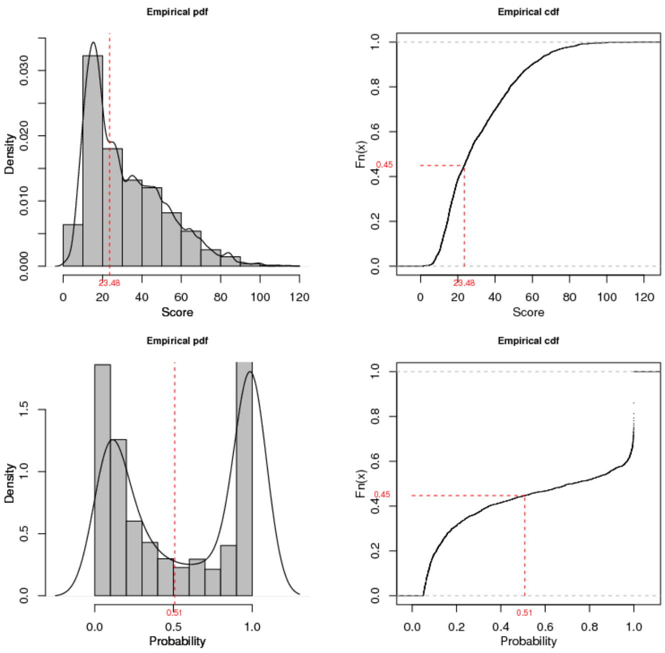


Empirical pdf and cdf plots of peptide sequence scores and probabilities
(NME2 experiment)

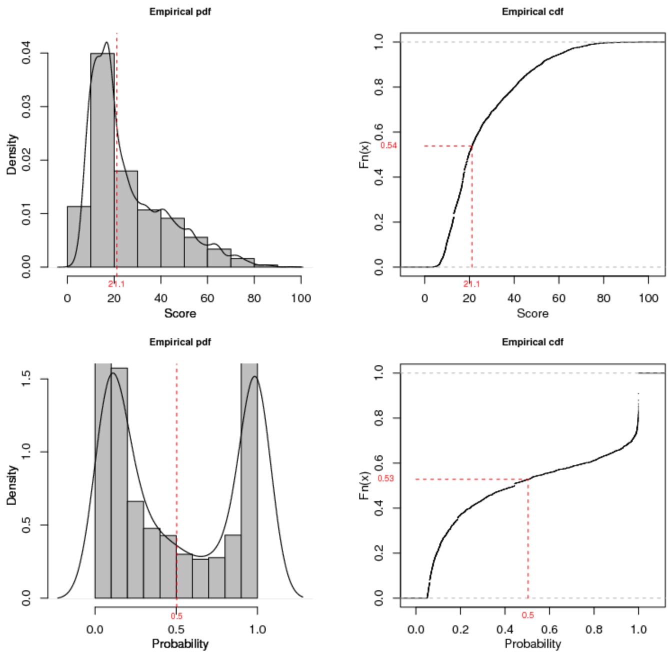


Empirical pdf and cdf plots of peptide sequence scores and probabilities
(PPM1B experiment)

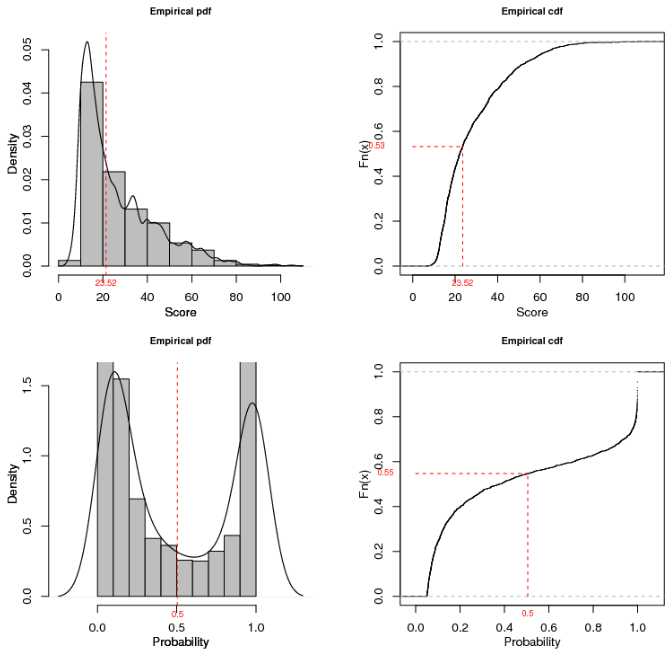


Empirical pdf and cdf plots of peptide sequence scores and probabilities
(VHL experiment)

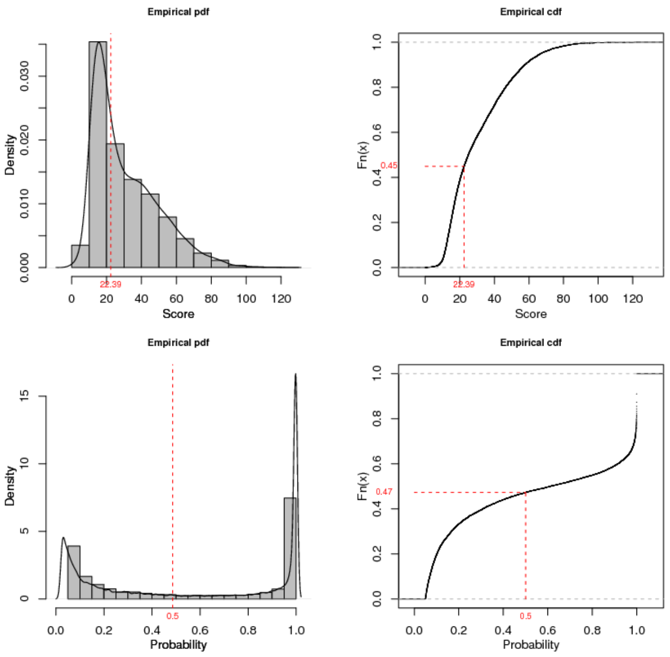

**Additional file 2: Figure S4**: Optimizing the determination of the peptide *MASCOT* score threshold in all AP-MS bait experiments (CTNNBIP1, STK24, NME2, PPM1B, and VHL). The optimal peptide *MASCOT Score Threshold* (*MST*) is shown as well as the *Reproducibility Index Threshold* (*RIT*) with corresponding *Reproducibility Index* (*RI*).

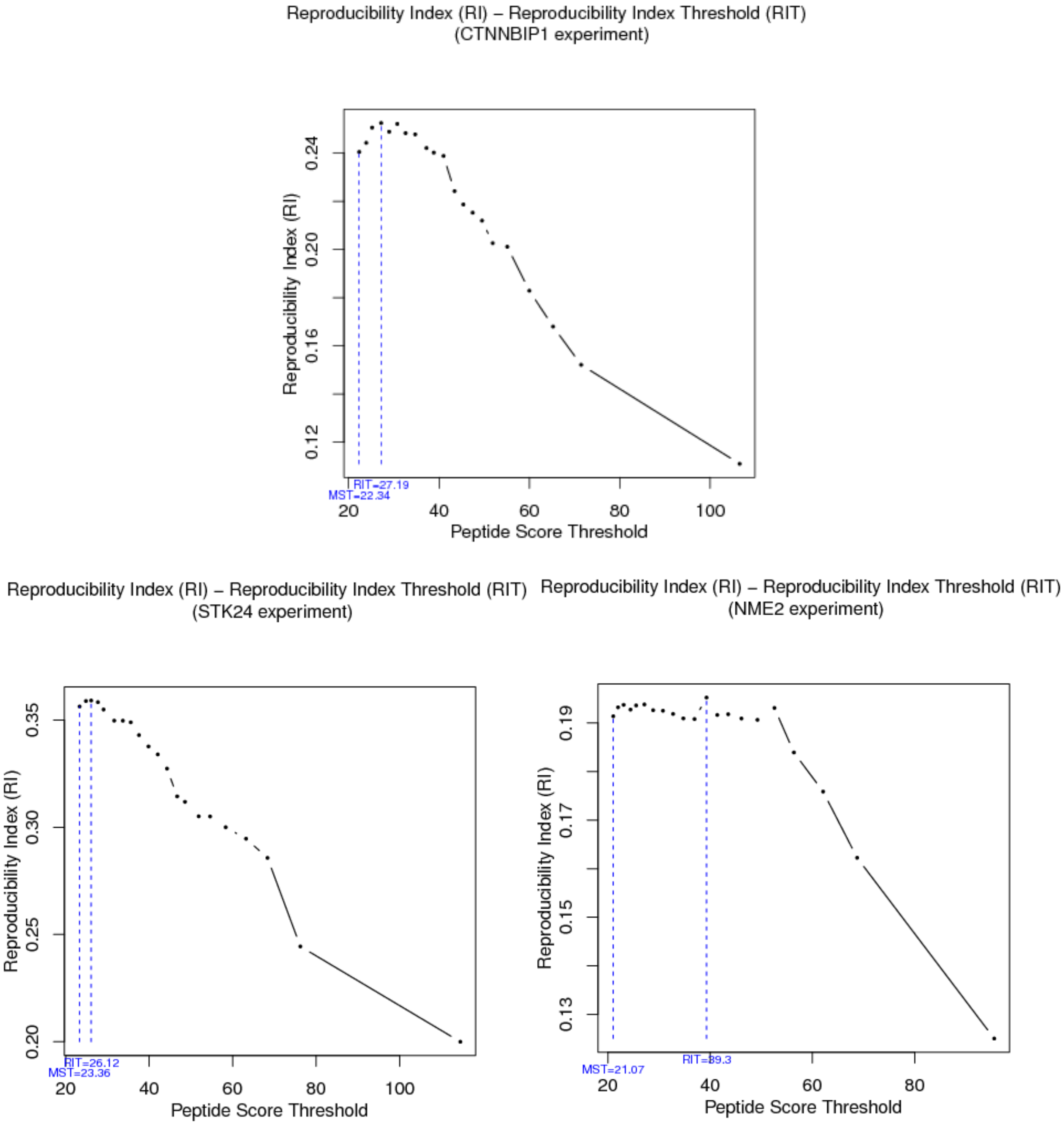

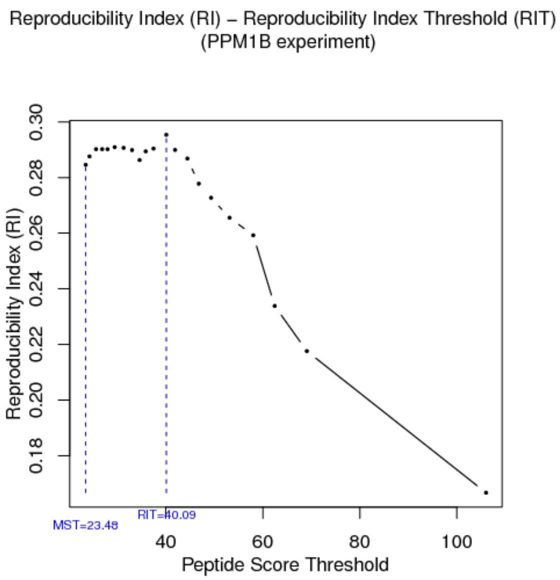
Reproducibility Index (RI) – Reproducibility Index Threshold (RIT)
(PPM1B experiment)
Reproducibility Index (RI)
0.18
0.20
0.22
0.24
0.26
0.28
0.30
MST=23.48
RIT=40.09
40
60
80
100
Peptide Score Threshold

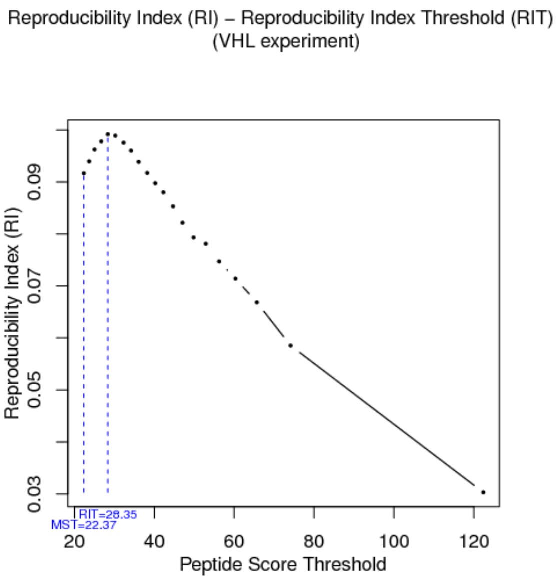
Reproducibility Index (RI) – Reproducibility Index Threshold (RIT)
(VHL experiment)
Reproducibility Index (RI)
0.03
0.05
0.07
0.09
RIT=28.35
MST=22.37
20
40
60
80
100
120
Peptide Score Threshold

**Additional file 2: Figure S5**: Number of *Indicator Prey Proteins* $\hat{Q}^B(\tilde{p}^B_{\min})$ and *Reproducible Experimental Replicates* $\hat{L}^B(\tilde{p}^B_{\min})$ (left) and the *joint inclusion probability* $\hat{p}^B_J(\tilde{p}^B_{\min})$ (right) for the protein-based analysis in all AP-MS bait experiments (CTNNBIP1, STK24, NME2, PPM1B, and VHL). The number of *Indicator Prey Proteins*, *Reproducible Experimental Replicates*, and *joint inclusion probabilities* are indicated for the marginal inclusion probability threshold $\tilde{p}^B_{\min}$ as determined for each individual bait experiment (see Tables 1 and 3).

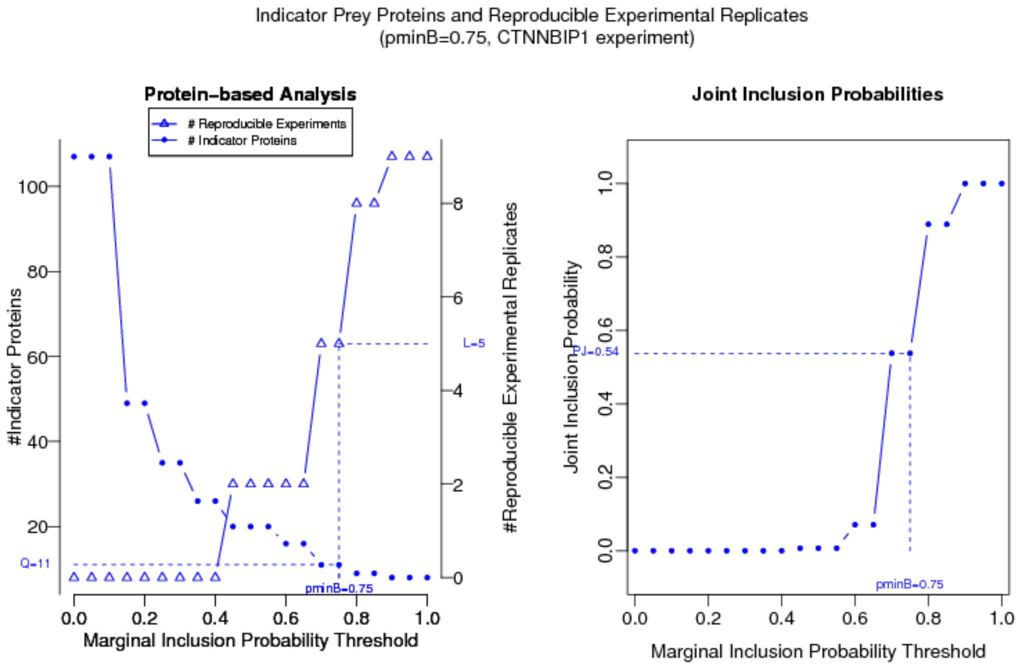

Indicator Prey Proteins and Reproducible Experimental Replicates
(pminB=0.80, STK24 experiment)

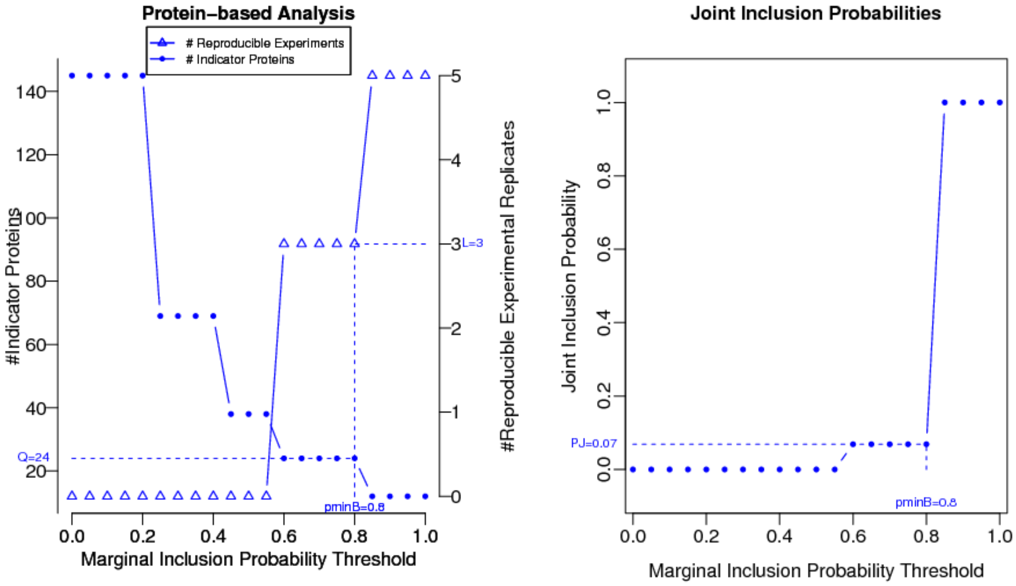


Indicator Prey Proteins and Reproducible Experimental Replicates
(pminB=0.80, NME2 experiment)

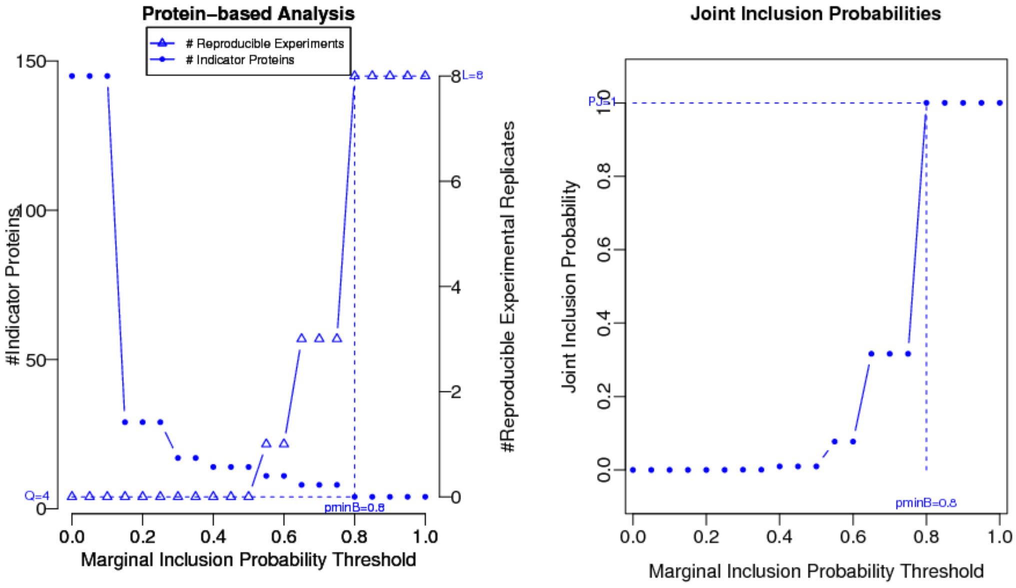

Indicator Prey Proteins and Reproducible Experimental Replicates
(pminB=0.80, PPM1B experiment)

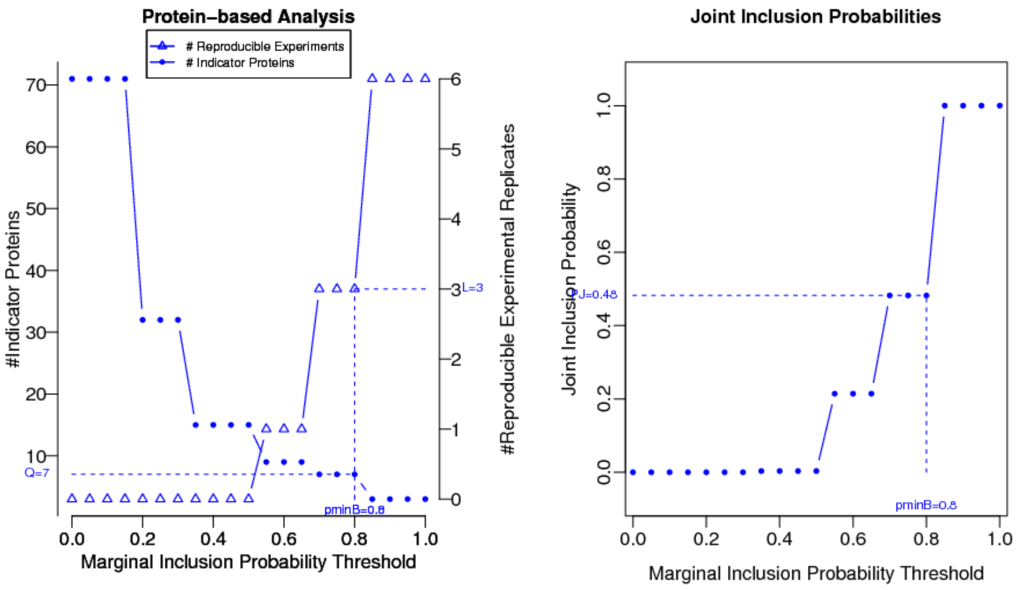


Indicator Prey Proteins and Reproducible Experimental Replicates
(pminB=0.75, VHL experiment)

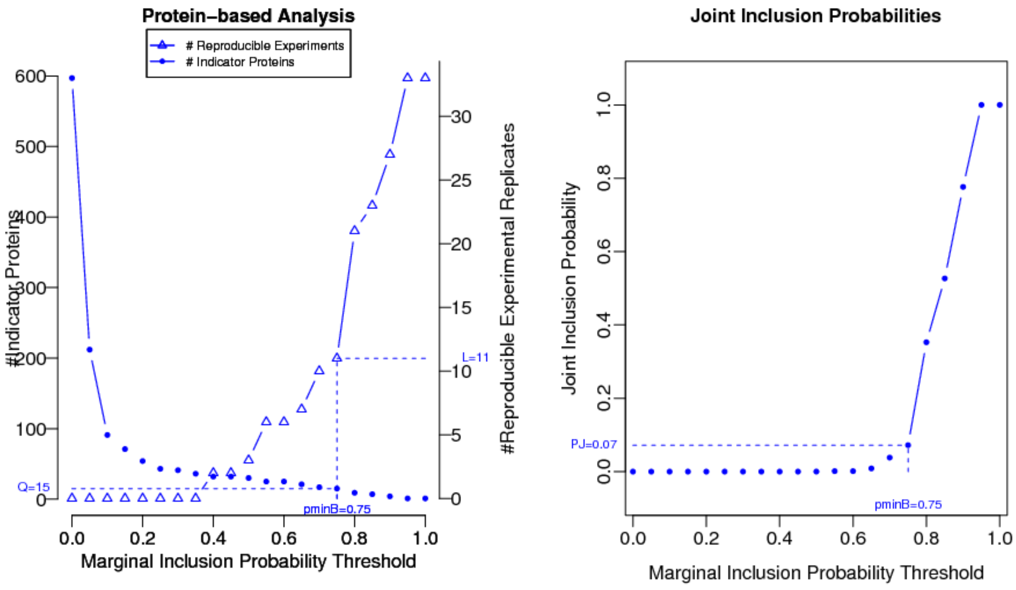

**Additional file 2: Figure S6**: *FDR* sensitivity as a function of *Confidence Score* cutoff and marginal inclusion probability threshold in the CTNNBIP1 AP-MS bait experiment. *FDR* estimates of bait-prey PPI are reported with standard errors. Horizontal black dotted lines correspond to thresholds of *FDR* level $(\theta = 0.05)$. Results are reported for the range of the *Confidence Score* cutoffs $\hat{C}_S^{cutoff} \in (0, 0.2]$ and $p_{\min}^B \in [0.45, 1.00]$ of the marginal inclusion probability threshold.

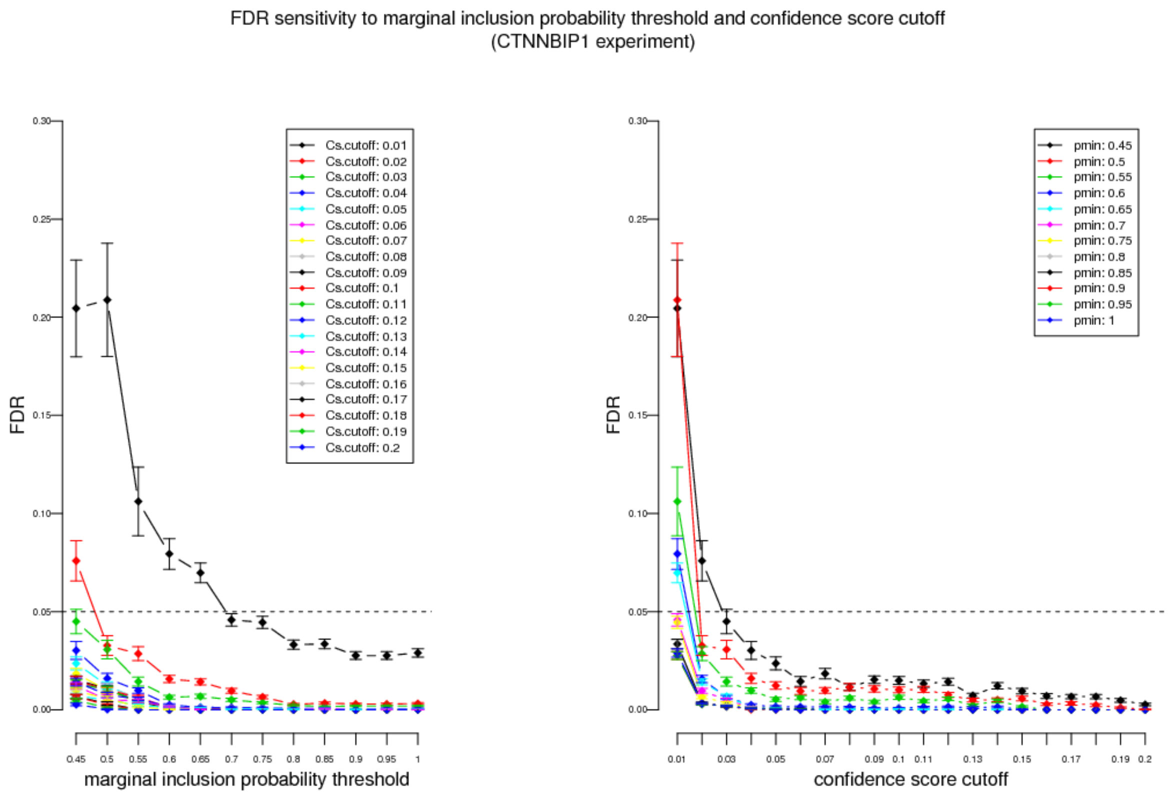

**Additional file 2: Figure S7**: *FDR* and *GO* semantic similarity analyses in all AP-MS bait experiments (CTNNBIP1, STK24, NME2, PPM1B, and VHL). Left: Log-plot of *FDR* for the bait-prey PPIs are plotted against *Confidence Score* cutoffs $C_S^{cutoff}$. Estimates of $\log_{10}(10^{-3}+FDR)$ are reported with standard errors. The horizontal black dotted line corresponds to the threshold of *FDR* significance level $(\theta = 0.05)$. Right: estimates of distance $d\left(C_S^{cutoff}\right)$ (see Methods section) are plotted with standard errors against *Confidence Score* cutoffs $C_S^{cutoff}$. The horizontal dotted line corresponds to the threshold of *GO* significance $(d > 0)$. For the computation of *d*, approximate 95% Confidence Interval of median bait-prey semantic similarities were carried out in the initial set of bait experiments ("*N*" stage) and the set of bait *Reproducible Experimental Replicates* ("*S*" stage) for the Molecular Function (MF) ontology as described in method section. Here *B* = 1024 Monte-Carlo replicates were performed, and a coefficient $c = 1.386$ was chosen for the 95% CI since group sample sizes and group standard deviations were similar [32]. Results are reported for the range $\hat{C}_S^{cutoff} \in (0, 0.2]$ of *Confidence Score* cutoff and for the marginal inclusion probability threshold $\tilde{p}_{\min}^{B}$ as determined in each individual bait experiment (see Tables 1 and 3).

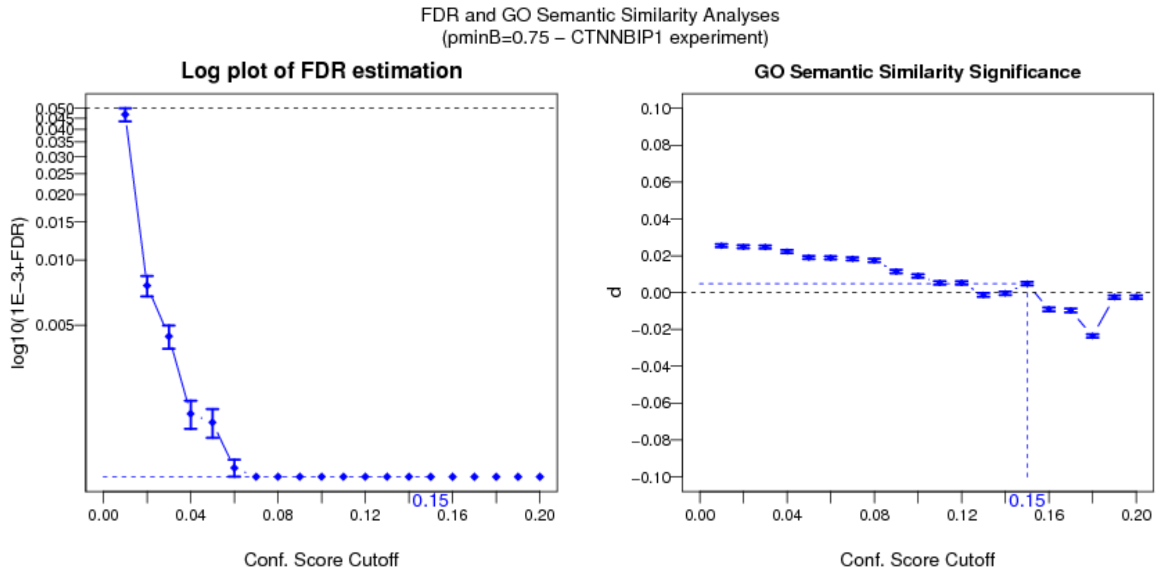

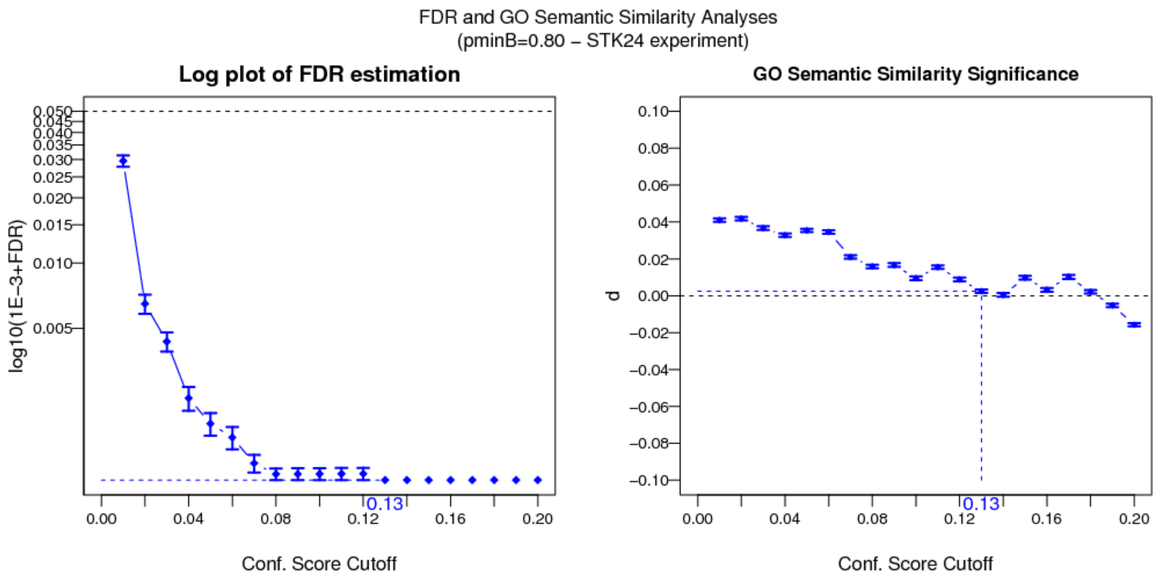
FDR and GO Semantic Similarity Analyses
(pminB=0.80 – STK24 experiment)
Log plot of FDR estimation
log10(1E−3+FDR)
0.050
0.045
0.040
0.035
0.030
0.025
0.020
0.015
0.010
0.005
0.13
0.00
0.04
0.08
0.12
0.16
0.20
Conf. Score Cutoff
GO Semantic Similarity Significance
d
0.10
0.08
0.06
0.04
0.02
0.00
−0.02
−0.04
−0.06
−0.08
−0.10
0.13
0.00
0.04
0.08
0.12
0.16
0.20
Conf. Score Cutoff

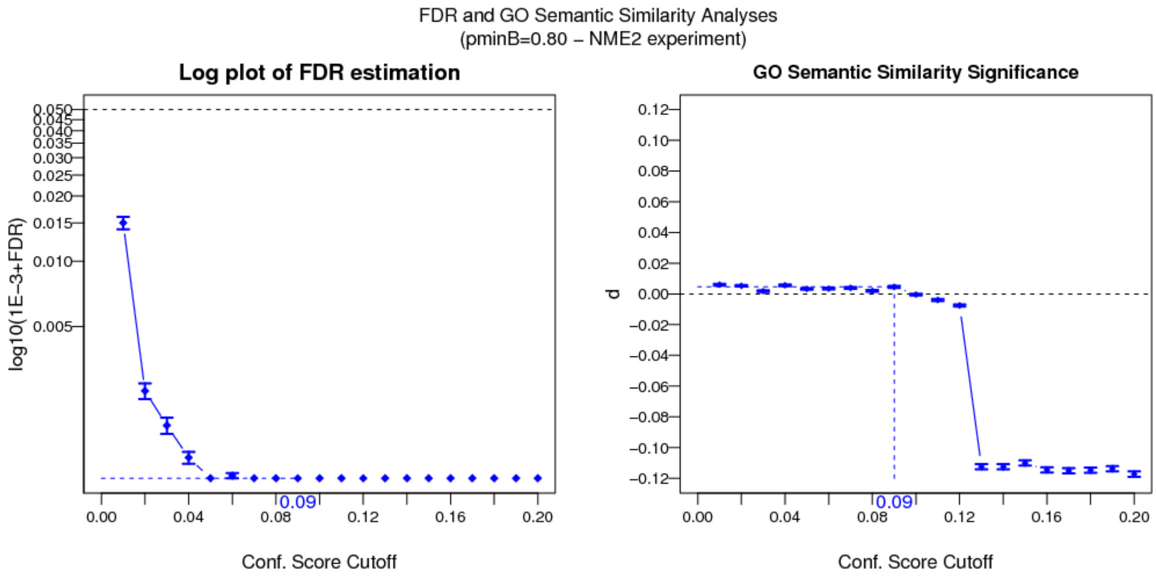
FDR and GO Semantic Similarity Analyses
(pminB=0.80 – NME2 experiment)
Log plot of FDR estimation
log10(1E−3+FDR)
0.050
0.045
0.040
0.035
0.030
0.025
0.020
0.015
0.010
0.005
0.09
0.00
0.04
0.08
0.12
0.16
0.20
Conf. Score Cutoff
GO Semantic Similarity Significance
d
0.12
0.10
0.08
0.06
0.04
0.02
0.00
−0.02
−0.04
−0.06
−0.08
−0.10
−0.12
0.09
0.00
0.04
0.08
0.12
0.16
0.20
Conf. Score Cutoff

FDR and GO Semantic Similarity Analyses
(pminB=0.80 – PPM1B experiment)

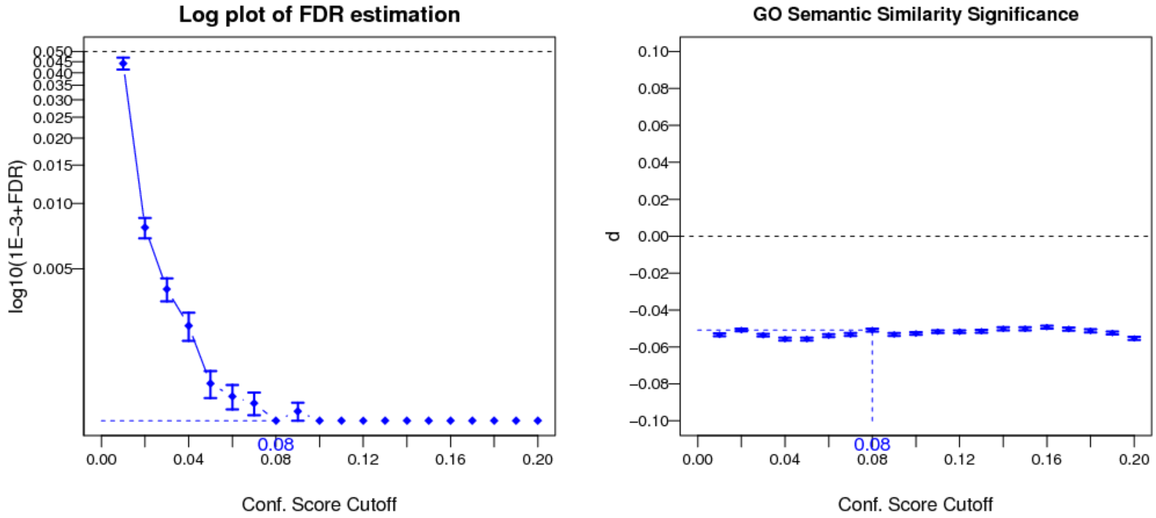


FDR and GO Semantic Similarity Analyses
(pminB=0.75 – VHL experiment)

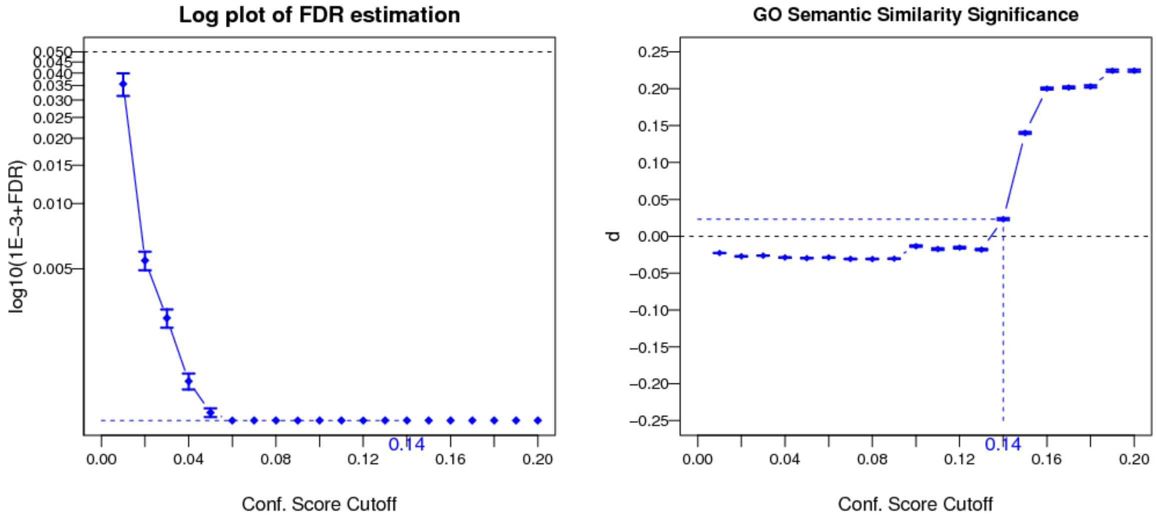

**Additional file 2: Figure S8**: Density distribution plots of bait-prey *Confidence Scores* at procedural stages "*N*", "*R*" and "*S*" in all AP-MS bait experiments (CTNNBIP1, STK24, NME2, PPM1B, and VHL). Note the identical density scales and the re-distribution of *Specific Prey Proteins* (*Confidence Score* → 1) as the method progresses through the procedural stages: from the initial "Naïve" stage ("*N*"), to the "Reproducible" stage ("*R*"), and to the final "Specific" stage ("*S*"). Results are reported for the entire positive range $\hat{C}_S^{cutoff} \in [0,1]$ of *Confidence Score* cutoff and the marginal inclusion probability thresholds $\tilde{p}_{\min}^{C}$ and $\tilde{p}_{\min}^{B}$ as determined in each AP-MS experiment (see Tables 1 and 3).

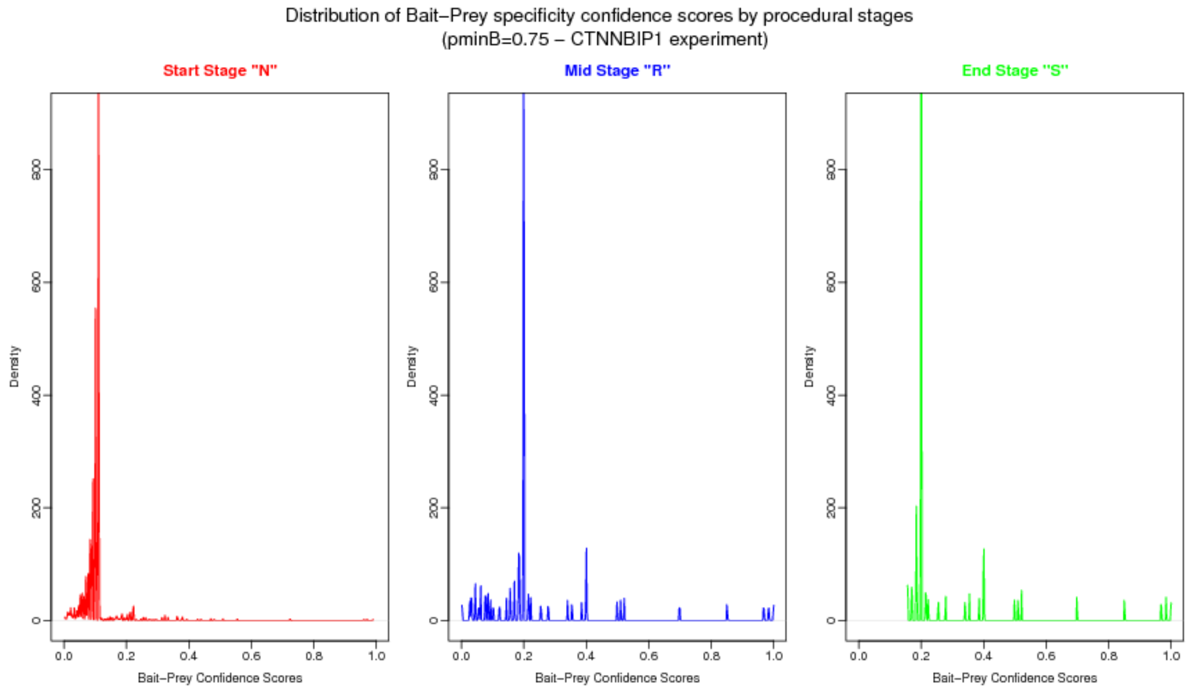

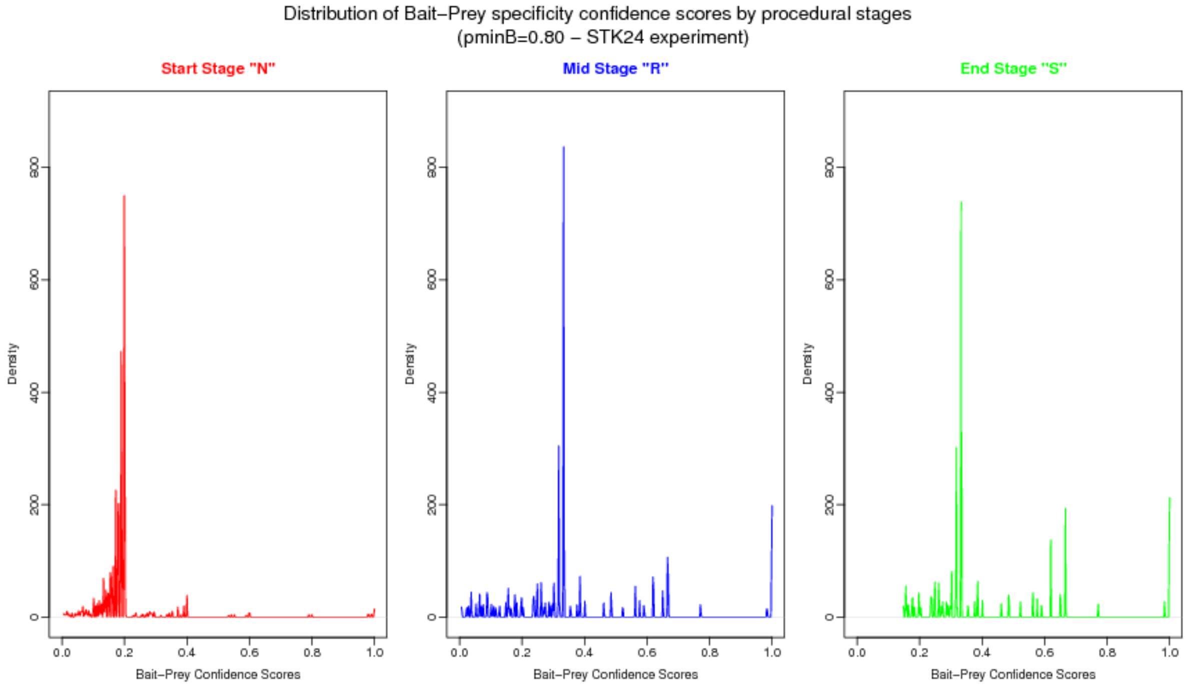
Distribution of Bait–Prey specificity confidence scores by procedural stages
(pminB=0.80 – STK24 experiment)
Start Stage "N"
Mid Stage "R"
End Stage "S"
Density
Bait–Prey Confidence Scores

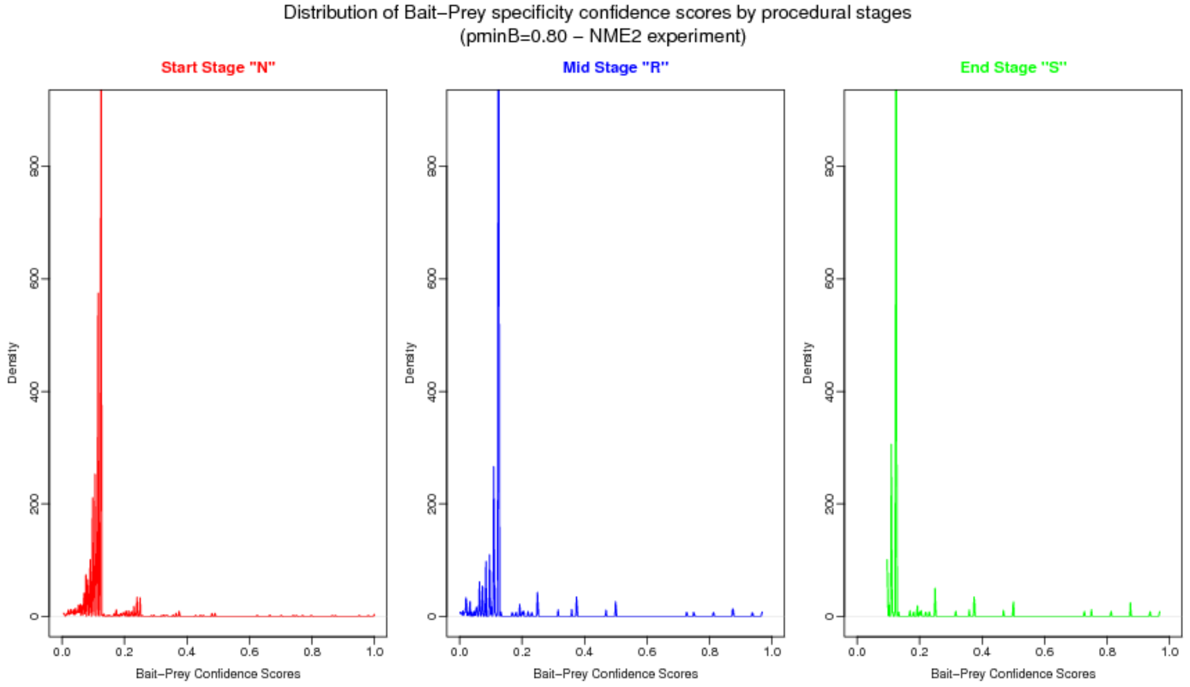
Distribution of Bait–Prey specificity confidence scores by procedural stages
(pminB=0.80 – NME2 experiment)
Start Stage "N"
Mid Stage "R"
End Stage "S"
Density
Bait–Prey Confidence Scores

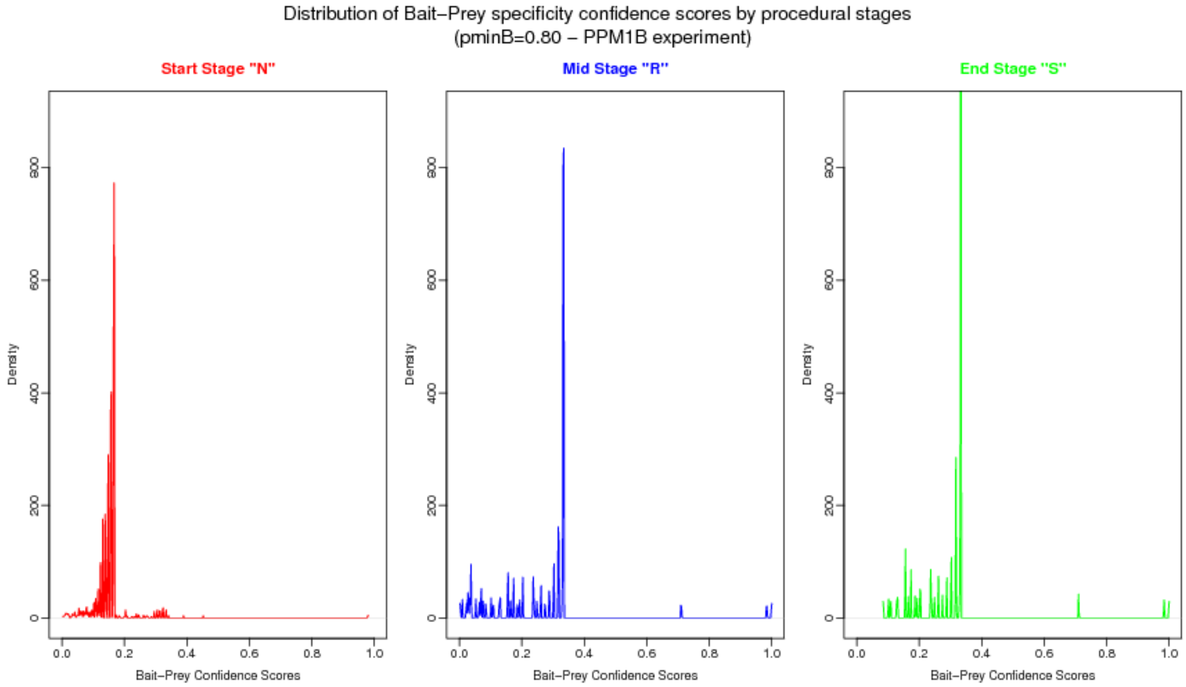
Distribution of Bait–Prey specificity confidence scores by procedural stages
(pminB=0.80 – PPM1B experiment)
Start Stage "N"
Mid Stage "R"
End Stage "S"
Density
Bait–Prey Confidence Scores

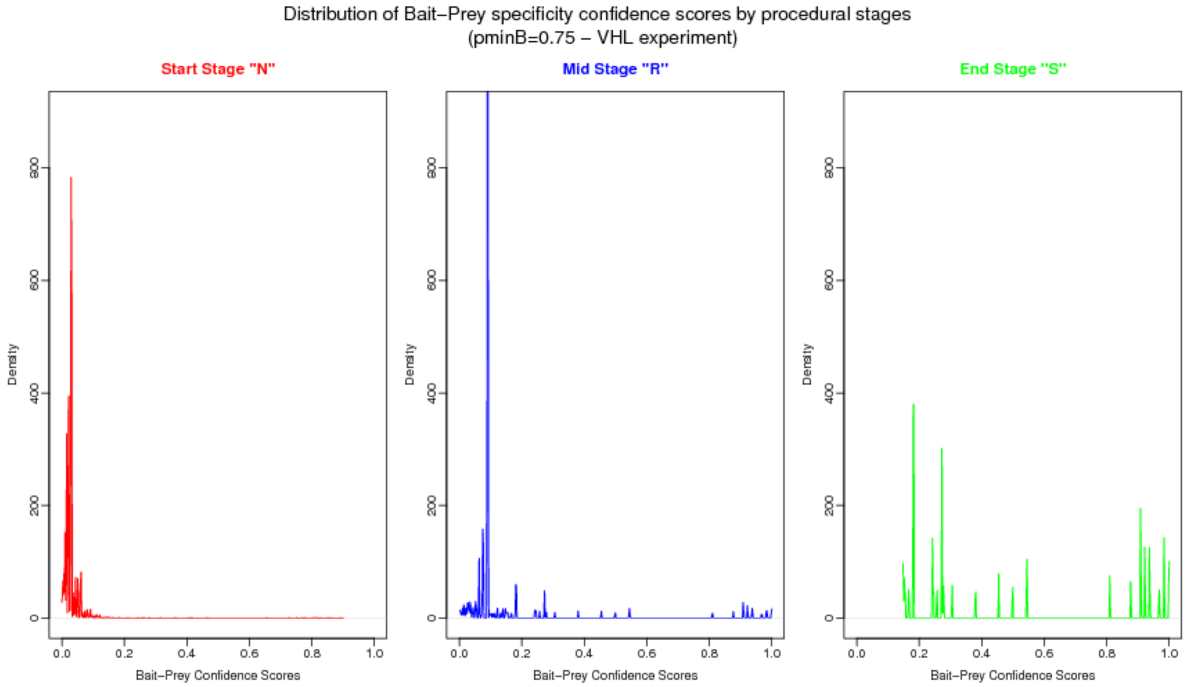
Distribution of Bait–Prey specificity confidence scores by procedural stages
(pminB=0.75 – VHL experiment)
Start Stage "N"
Mid Stage "R"
End Stage "S"
Density
Bait–Prey Confidence Scores

**Additional file 2: Figure S9**: Quantile-Quantile plots of bait vs. control *marginal* inclusion probabilities in all AP-MS bait experiments (CTNNBIP1, STK24, NME2, PPM1B, and VHL). Note the increase in quantiles in the bait compared to the control experiment as the method progresses through the procedural stages, denoted by the initial "Naïve" ("*N*"), "Reproducible" ("*R*"), and finally "Specific" ("*S*"). This corresponds to a separation of bait versus control distributions with an accumulation towards 1 in the bait $\left(\hat{p}'^{B}_{\mathrm{M}}(j,0.75)\rightarrow 1\right)$ vs. towards 0 in the control $\left(\hat{p}'^{C}_{\mathrm{M}}(j,0.75)\rightarrow 0\right)$. Results are reported for $C_S > \hat{C}_S^{cutoff}$ and the marginal inclusion probability thresholds $\tilde{p}^{C}_{\min}$ and $\tilde{p}^{B}_{\min}$ as determined in each AP-MS experiment (see Tables 1 and 3).

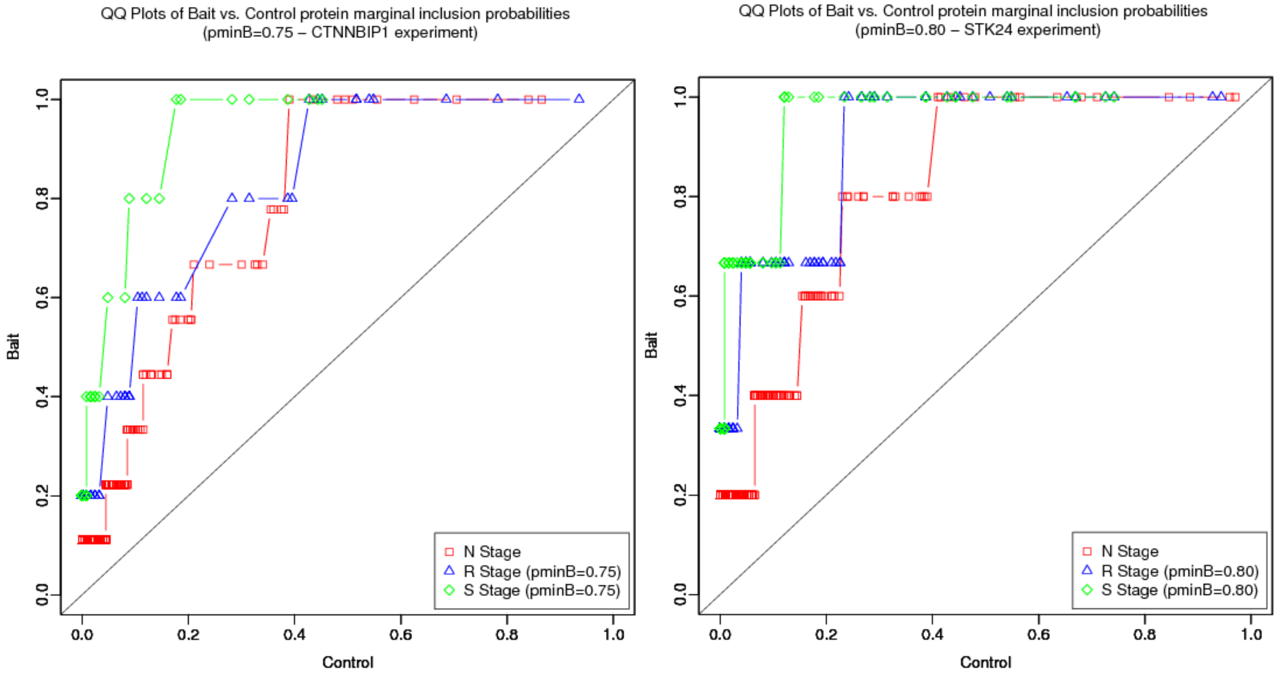

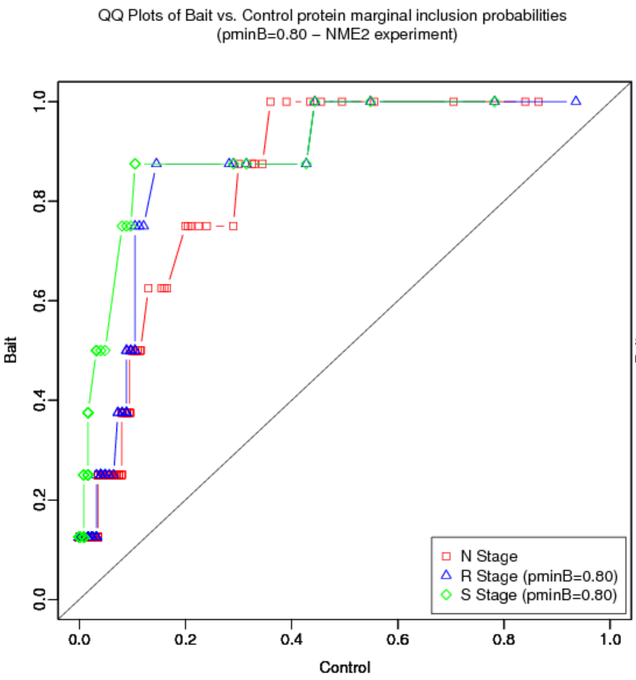
QQ Plots of Bait vs. Control protein marginal inclusion probabilities
(pminB=0.80 – NME2 experiment)
Bait
Control
N Stage
R Stage (pminB=0.80)
S Stage (pminB=0.80)

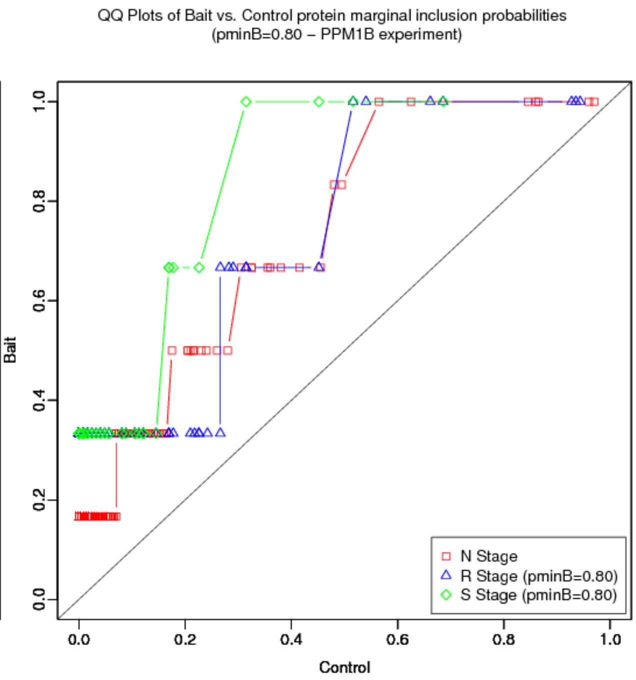
QQ Plots of Bait vs. Control protein marginal inclusion probabilities
(pminB=0.80 – PPM1B experiment)
Bait
Control
N Stage
R Stage (pminB=0.80)
S Stage (pminB=0.80)

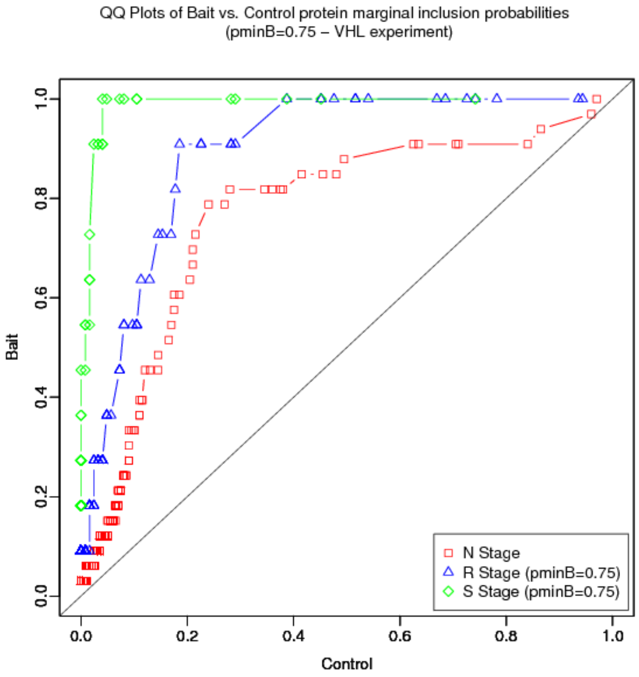
QQ Plots of Bait vs. Control protein marginal inclusion probabilities
(pminB=0.75 – VHL experiment)
Bait
Control
N Stage
R Stage (pminB=0.75)
S Stage (pminB=0.75)

**Additional file 2: Figure S10**: Correlation and regression relationships between protein *MASCOT* scores and protein marginal inclusion probabilities at different procedural stages from the initial "Naïve" stage ("*N*"), to the "Reproducible" stage ("*R*"), and to the final "Specific" stage ("*S*") in all AP-MS bait experiments (CTNNBIP1, STK24, NME2, PPM1B, and VHL). Results are reported for $C_S > \hat{C}_S^{cutoff}$ and the marginal inclusion probability thresholds $\tilde{p}^C_{\min}$ and $\tilde{p}^B_{\min}$ as determined in each AP-MS experiment (see Tables 1 and 3). See Additional file 1 Supplemental Methods for more details on how of protein *MASCOT* scores and protein marginal inclusion probabilities are computed [Additional file 1: Supplemental Methods].

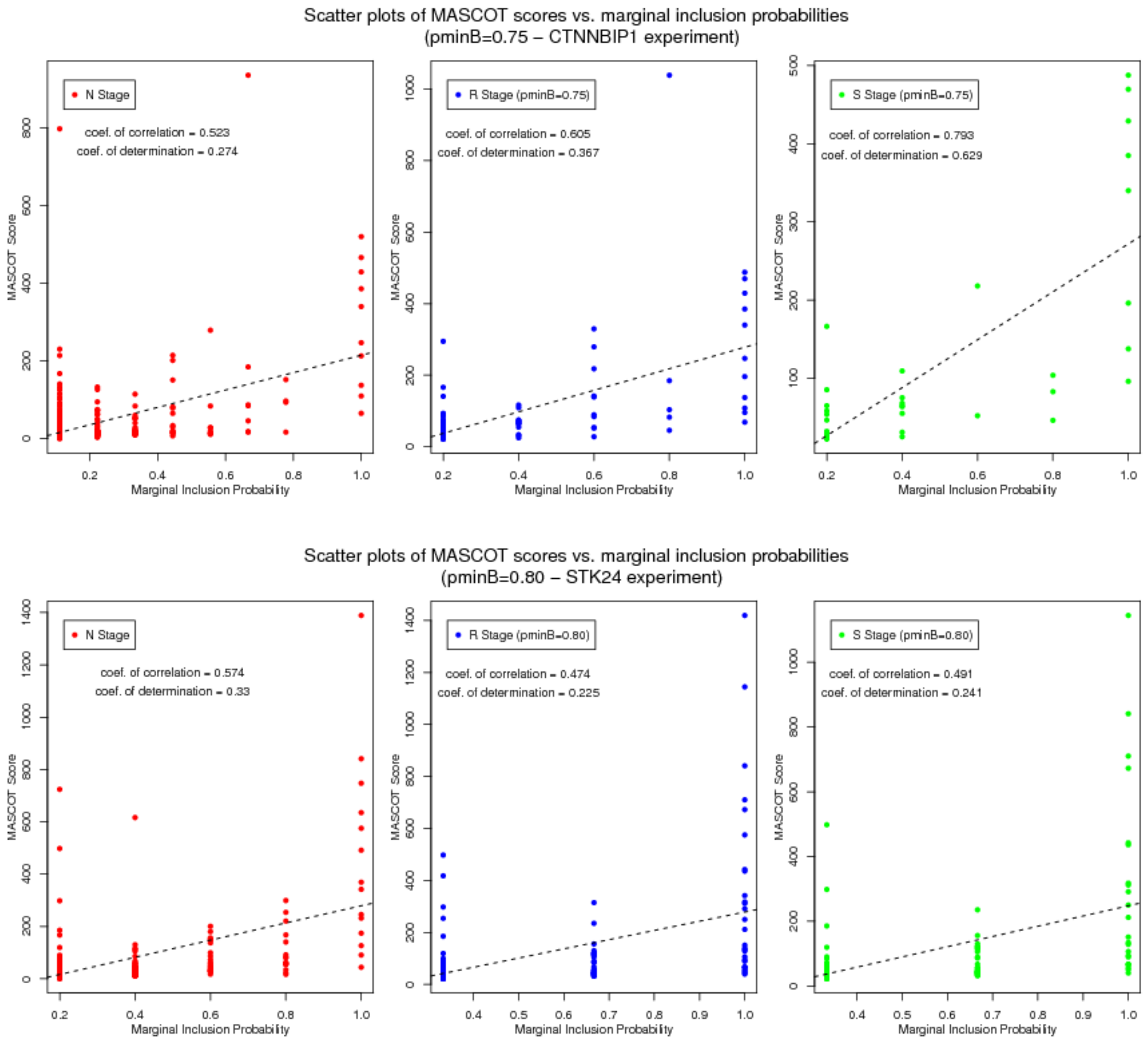

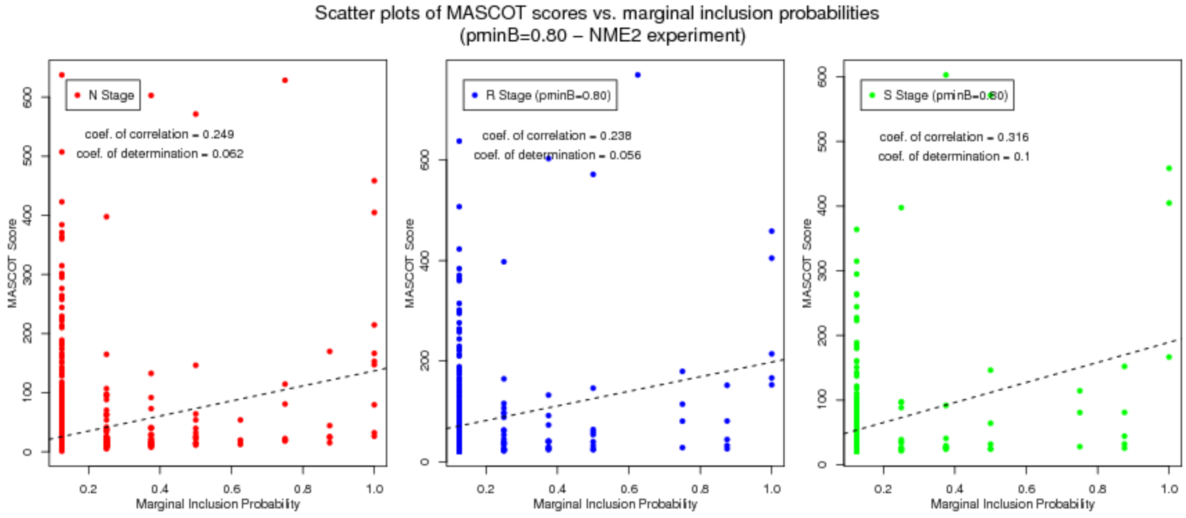
Scatter plots of MASCOT scores vs. marginal inclusion probabilities
(pminB=0.80 – NME2 experiment)
N Stage
coef. of correlation = 0.249
coef. of determination = 0.062
R Stage (pminB=0.80)
coef. of correlation = 0.238
coef. of determination = 0.056
S Stage (pminB=0.80)
coef. of correlation = 0.316
coef. of determination = 0.1
MASCOT Score
Marginal Inclusion Probability

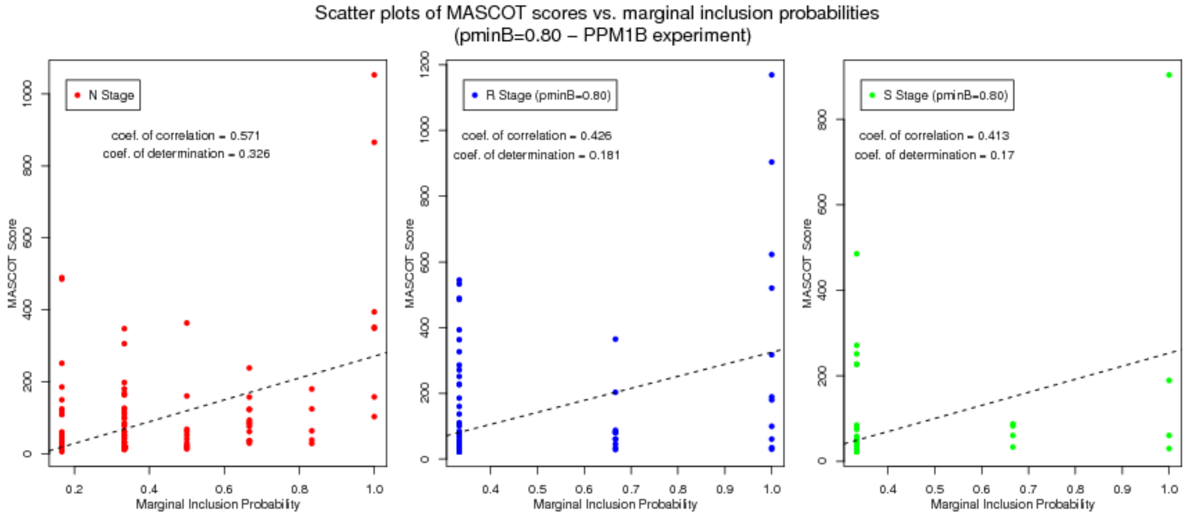
Scatter plots of MASCOT scores vs. marginal inclusion probabilities
(pminB=0.80 – PPM1B experiment)
N Stage
coef. of correlation = 0.571
coef. of determination = 0.326
R Stage (pminB=0.80)
coef. of correlation = 0.426
coef. of determination = 0.181
S Stage (pminB=0.80)
coef. of correlation = 0.413
coef. of determination = 0.17
MASCOT Score
Marginal Inclusion Probability

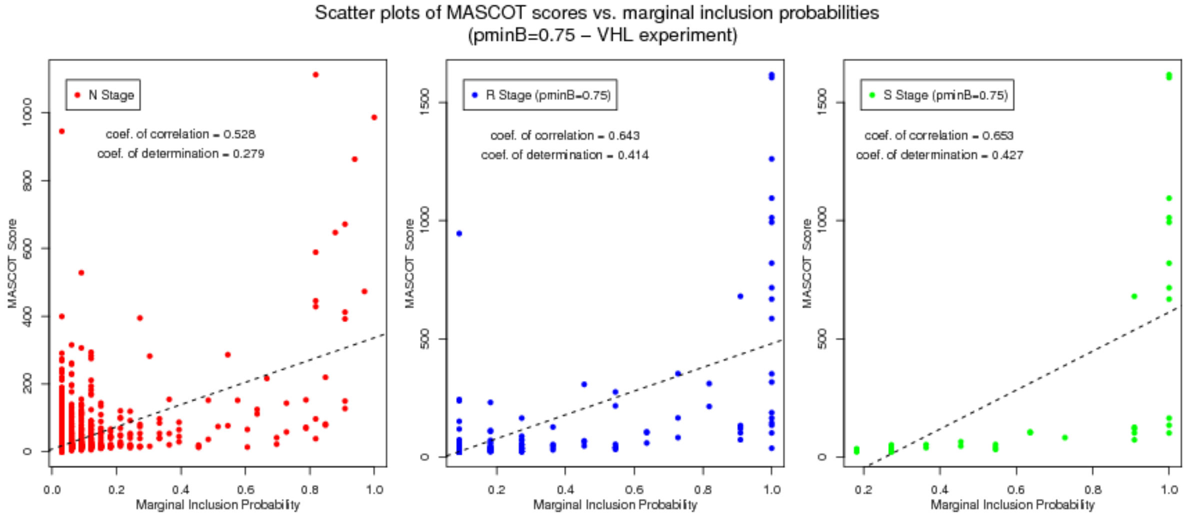
Scatter plots of MASCOT scores vs. marginal inclusion probabilities
(pminB=0.75 – VHL experiment)
N Stage
coef. of correlation = 0.528
coef. of determination = 0.279
R Stage (pminB=0.75)
coef. of correlation = 0.643
coef. of determination = 0.414
S Stage (pminB=0.75)
coef. of correlation = 0.653
coef. of determination = 0.427
MASCOT Score
Marginal Inclusion Probability

**Additional file 2: Figure S11**: Stability of the Coefficient of Variation (CV) of the mean marginal inclusion probability as a function of procedural stages from the initial “Naïve” stage (“*N*”), to the “Reproducible” stage (“*R*”), and to the final “Specific” stage (“*S*”) in all AP-MS bait experiments (CTNNBIP1, STK24, NME2, PPM1B, and VHL). Results are reported for $C_S > \hat{C}_S^{cutoff}$ and the marginal inclusion probability thresholds $\tilde{p}_{\min}^{C}$ and $\tilde{p}_{\min}^{B}$ as determined in each AP-MS experiment (see Tables 1 and 3).

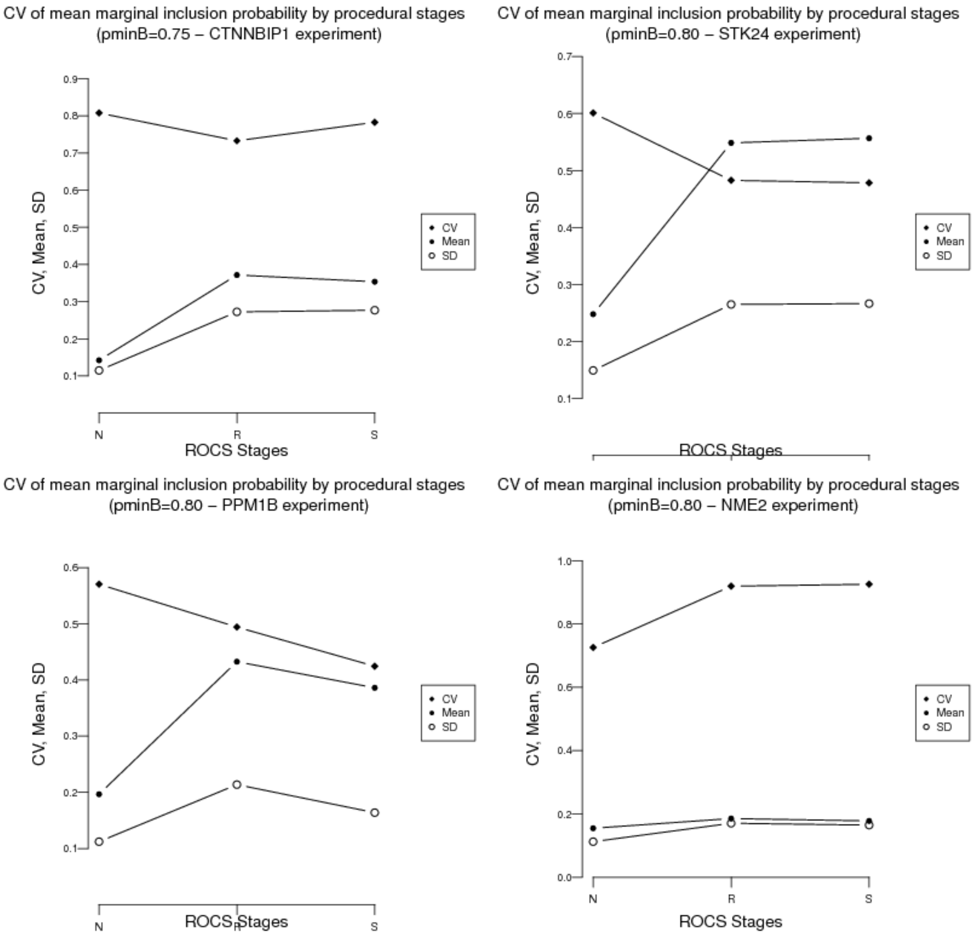

CV of mean marginal inclusion probability by procedural stages
(pminB=0.75 – VHL experiment)

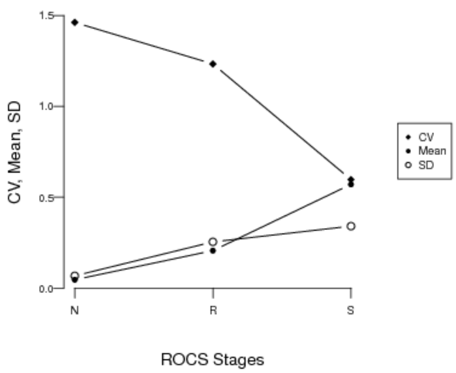

**Additional file 2: Figure S12**: Confidence Intervals (95% CIs) of the median bait-prey semantic similarity for *SAINT*-only and *SAINT* in conjunction with *ROCS* at the different *ROCS* procedural stages "*N*" (*SAINT*-only), "*R*", and "*S*" (*ROCS-SAINT*) in all AP-MS bait experiments (CTNNBIP1, STK24, NME2, PPM1B, and VHL). Note especially the increase in median bait-prey semantic similarity between the naïve stage "*N*" (*SAINT*-only) and final stage "*S*" (*ROCS-SAINT*). The pairwise Resnik semantic similarity measure $sim(c_B, c_P)$ is given between a Gene Ontology (*GO*) term from the bait protein (denoted $c_B$) and for each *Specific Prey Protein* (denoted $c_P$) as described in the methods section for all gene ontologies (Biological Process (BP), Molecular Function (MF), and Cellular Component (CC)) - see also Table S4 [Additional file 6: Table S4]. Note that the 95% CI of the median for the BP Gene Ontology of the VHL bait experiment could not be computed due to the very small output list (i.e. sample size $n = 2$) from *SAINT* – see Table S4 [Additional file 6: Table S4]. Results are reported here for a conservative coefficient of $c = 1.960$ for the 95% CI of the median since group sample sizes and group standard deviations were *not* similar (see methods section), and for $C_S > \hat{C}_S^{cutoff}$ and the marginal inclusion probability thresholds $\tilde{p}^C_{\min}$ and $\tilde{p}^B_{\min}$ as determined in each AP-MS experiment (see Tables 1 and 3).

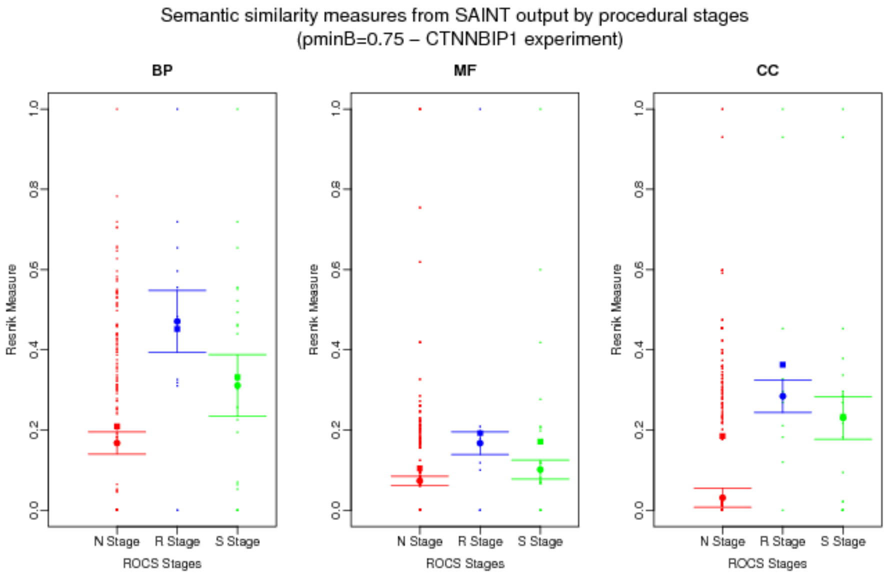

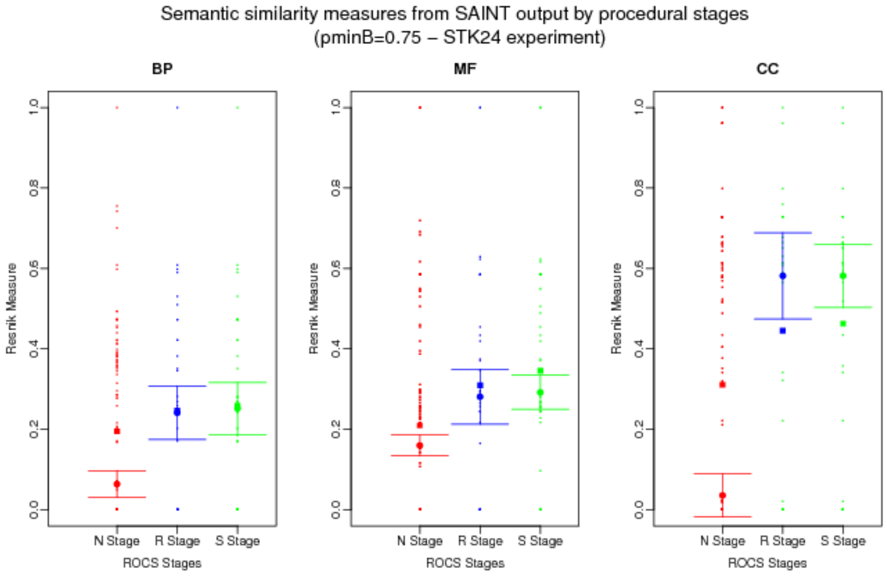
Semantic similarity measures from SAINT output by procedural stages
(pminB=0.75 – STK24 experiment)
BP
MF
CC
Resnik Measure
0.0
0.2
0.4
0.6
0.8
1.0
N Stage
R Stage
S Stage
ROCS Stages

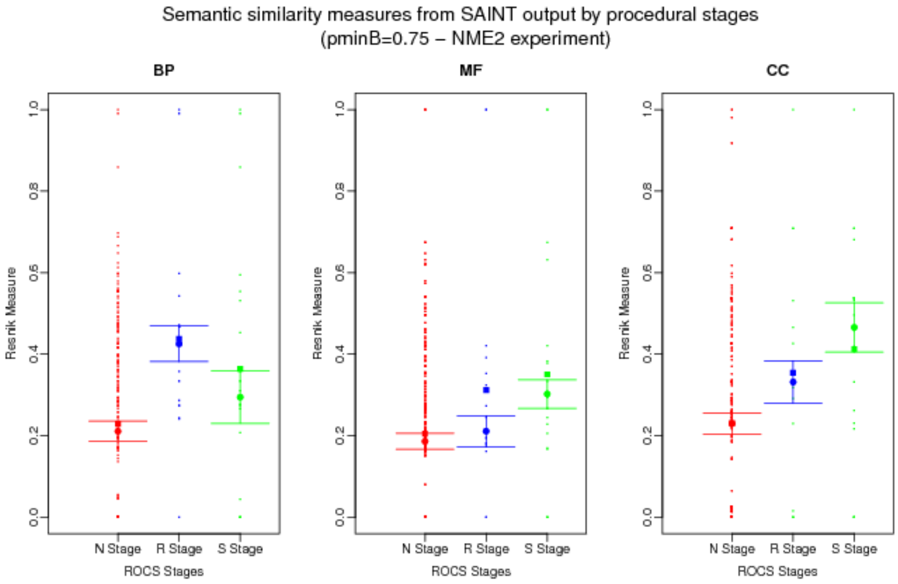
Semantic similarity measures from SAINT output by procedural stages
(pminB=0.75 – NME2 experiment)
BP
MF
CC
Resnik Measure
0.0
0.2
0.4
0.6
0.8
1.0
N Stage
R Stage
S Stage
ROCS Stages

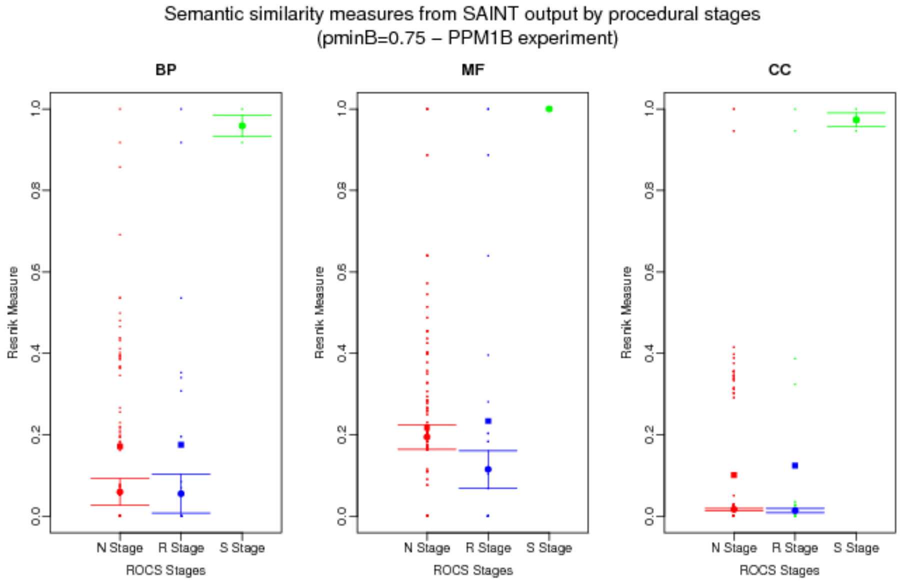
Semantic similarity measures from SAINT output by procedural stages
(pminB=0.75 – PPM1B experiment)
BP
MF
CC
Resnik Measure
0.0
0.2
0.4
0.6
0.8
1.0
N Stage
R Stage
S Stage
ROCS Stages

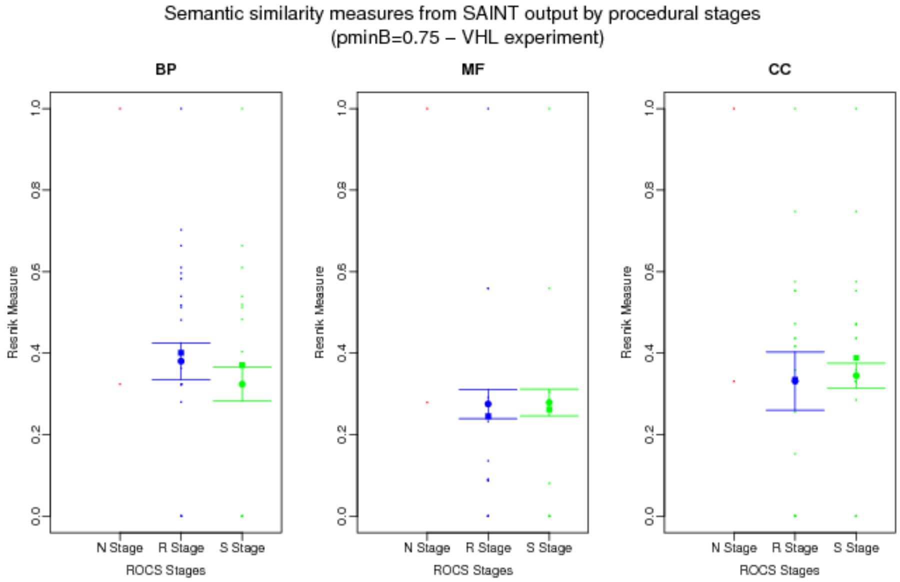
Semantic similarity measures from SAINT output by procedural stages
(pminB=0.75 – VHL experiment)
BP
MF
CC
Resnik Measure
0.0
0.2
0.4
0.6
0.8
1.0
N Stage
R Stage
S Stage
ROCS Stages

**Additional file 2: Figure S13**: *FDR* computed from *SAINT* posterior probability output ( $P_{SAINT}$ ) as a function of procedural stages: from the initial "Naïve" stage ("*N*" – *SAINT*-only), to the "Reproducible" stage ("*R*"), and to the final "Specific" stage ("*S*" – *ROCS-SAINT*) in all AP-MS bait experiments (CTNNBIP1, STK24, NME2, PPM1B, and VHL). Note especially the increase in median bait-prey semantic similarity between the naïve stage "*N*" (*SAINT*-only) and final stage "*S*" (*ROCS-SAINT*). Results are reported for $C_S > \hat{C}_S^{cutoff}$ and the marginal inclusion probability thresholds $\tilde{p}_{\min}^{C}$ and $\tilde{p}_{\min}^{B}$ as determined in each AP-MS experiment (see Tables 1 and 3).

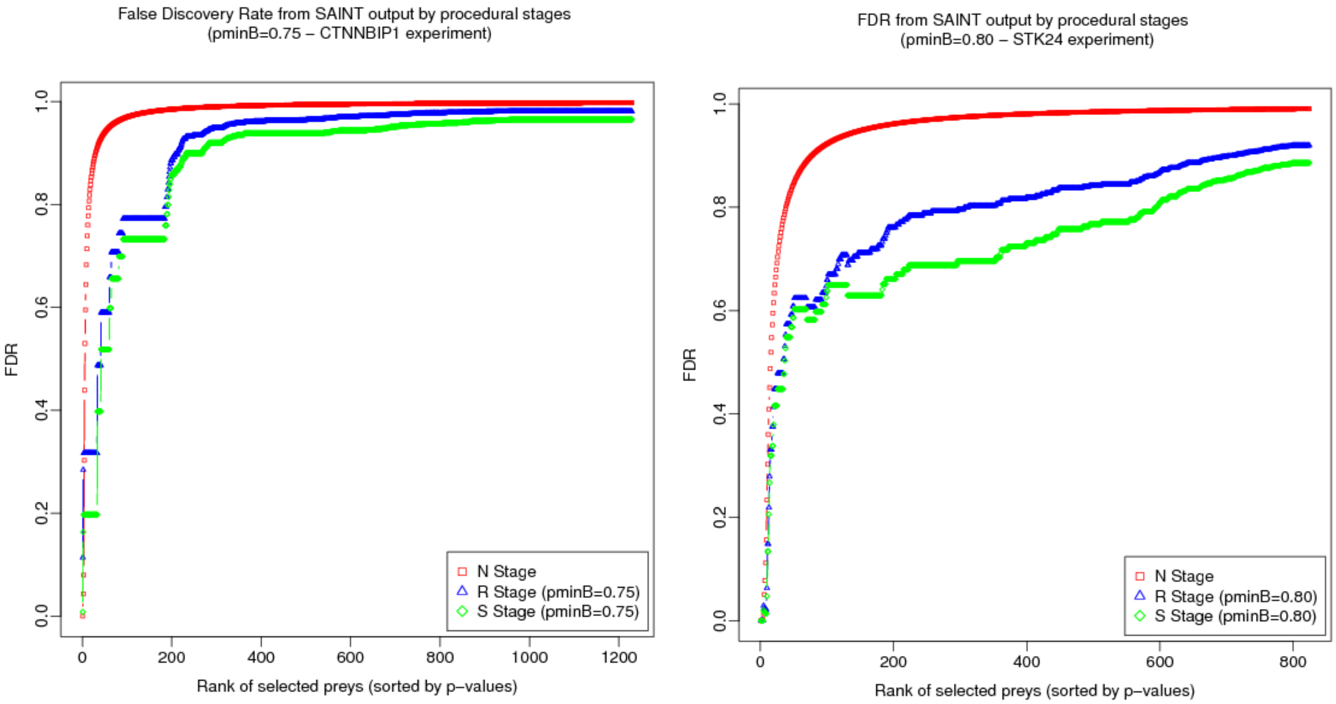

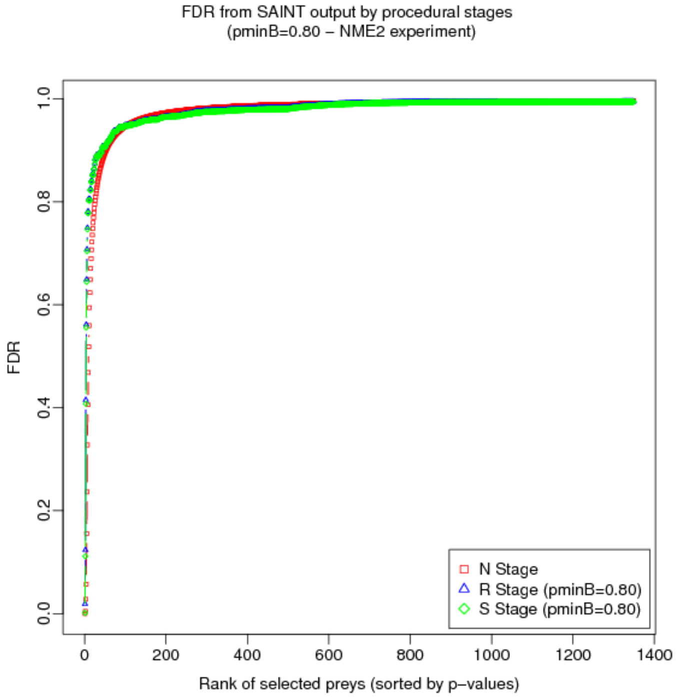
FDR from SAINT output by procedural stages
(pminB=0.80 – NME2 experiment)
FDR
0.0
0.2
0.4
0.6
0.8
1.0
0
200
400
600
800
1000
1200
1400
Rank of selected preys (sorted by p–values)
N Stage
R Stage (pminB=0.80)
S Stage (pminB=0.80)

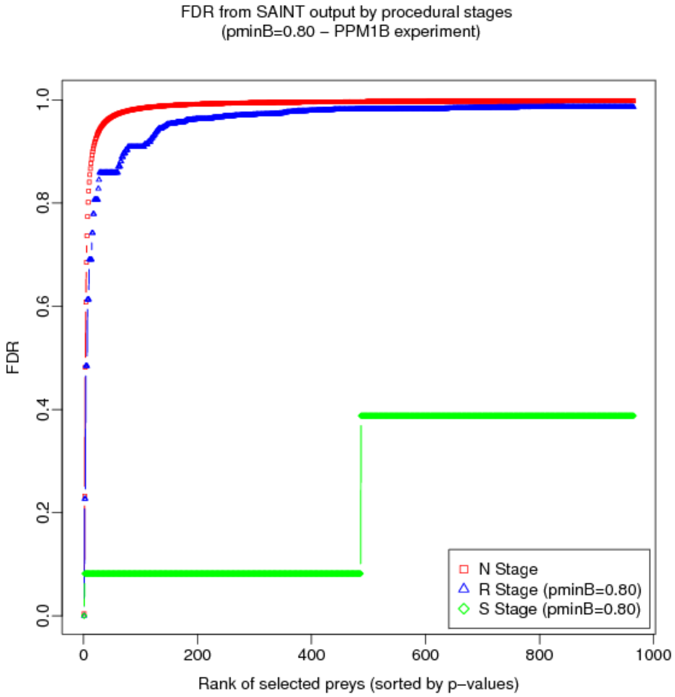
FDR from SAINT output by procedural stages
(pminB=0.80 – PPM1B experiment)
FDR
0.0
0.2
0.4
0.6
0.8
1.0
0
200
400
600
800
1000
Rank of selected preys (sorted by p–values)
N Stage
R Stage (pminB=0.80)
S Stage (pminB=0.80)

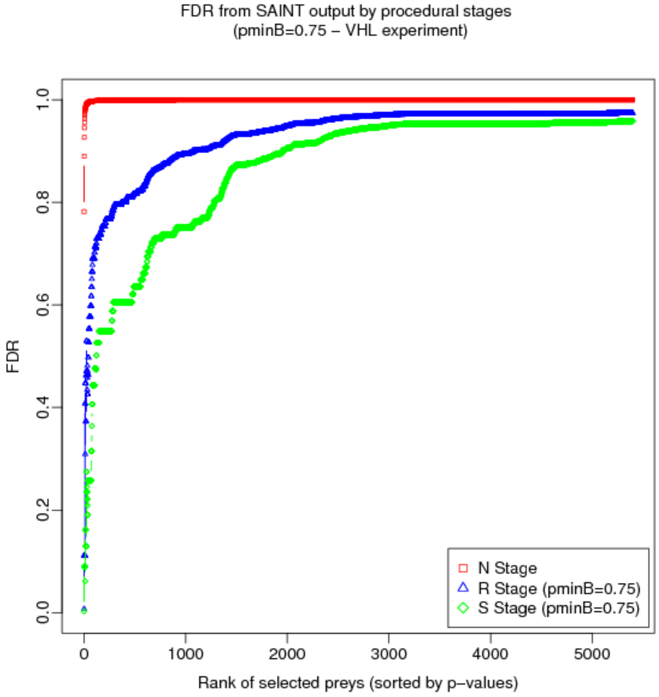
FDR from SAINT output by procedural stages
(pminB=0.75 – VHL experiment)
FDR
0.0
0.2
0.4
0.6
0.8
1.0
0
1000
2000
3000
4000
5000
Rank of selected preys (sorted by p–values)
N Stage
R Stage (pminB=0.75)
S Stage (pminB=0.75)

**Additional file 3: Table S1**: *ROCS* lists of *Indicator Prey Proteins* (IPI) and *Reproducible Experimental Replicates* (RER) in all AP-MS control and bait experiments.

**Additional file 4: Table S2**: Biological validation of *ROCS* protein-protein interaction (PPI) scoring results for the *Specific Prey Proteins*, ranked by *Confidence Score* $C_S$ in all AP-MS bait experiments (each on a separate Excel tab-sheet in a single file). The table on the left (green shaded) gives the matching of references from the *BioGRID* database (v. 3.1 - http://thebiogrid.org/) into the *ROCS* list. The number of publications, the Pubmed ID, and the experimental system used are reported. The table on the right (blue shaded) gives the reciprocal matching of the *ROCS* list into the *BioGRID* references. Results are reported for $C_S > \hat{C}_S^{cutoff}$ and the marginal inclusion probability thresholds $\tilde{p}_{\min}^{C}$ and $\tilde{p}_{\min}^{B}$ as determined in each AP-MS experiment (see Tables 1 and 3).

**Additional file 5: Table S3**: Comparison of protein-protein interaction (PPI) scoring for the *Specific Prey Proteins* between *SAINT* (Posterior Probability $P_{SAINT}$), *ComPASS* ($D-\text{score}$) and our method *ROCS* ($C-\text{score}$) in all AP-MS bait experiments (each on a separate Excel tab-sheet in a single file). In the table on the left (green shaded), all entries are ranked by decreasing significance of protein-protein interactions (PPI) according to each scoring method. The table on the right (blue shaded) gives the reciprocal matching of *SAINT* and *ComPASS* lists into the *ROCS* list. *SAINT* and *ComPASS* lists are reported for $P_{SAINT} > 0$ and $D-\text{score} > 0$ respectively. *ROCS* results are reported for $C-\text{score}$ $C_S > \hat{C}_S^{cutoff}$ and the marginal inclusion probability thresholds $\tilde{p}_{\min}^{C}$ and $\tilde{p}_{\min}^{B}$ as determined in each AP-MS experiment (see Tables 1 and 3).

**Additional file 6: Table S4**: Comparison of *SAINT* protein-protein interaction (PPI) scoring for the *Specific Prey Proteins* at the different *ROCS* procedural stages "*N*" (*SAINT*-only), "*R*", and "*S*" (*ROCS-SAINT*) in all AP-MS bait experiments (each on a separate Excel tab-sheet in a single file). Entries are ranked by decreasing significance of PPIs (Posterior Probability $P_{SAINT}$). The pairwise Resnik semantic similarity measure $sim(c_B, c_P)$ is given between a Gene Ontology (*GO*) term from the bait protein (denoted $c_B$) and for each *Specific Prey Protein* (denoted $c_P$) as

described in the methods section for all Gene Ontologies (Biological Process (BP), Molecular Function (MF), and Cellular Component (CC)) - see also Additional file 2: Figure S12. *SAINT* and *ComPASS* lists are reported for $P_{SAINT} > 0$ and $D-\text{score} > 0$ respectively. *ROCS* results are reported for $C-\text{score}$ $C_S > \hat{C}_S^{cutoff}$ and the marginal inclusion probability thresholds $\tilde{p}_{\min}^{C}$ and $\tilde{p}_{\min}^{B}$ as determined in each AP-MS experiment (see Tables 1 and 3).
